# Counterion influence on the N–I–N halogen bond†
†Electronic supplementary information (ESI) available: Experimental details of synthesis, compound characterisation, IPE NMR measurements, computational and crystallographic procedures, and crystal data for **1-Ag/I** to **7-Ag/I**, and **12-Ag**. CCDC 1045981–1045995. For ESI and crystallographic data in CIF or other electronic format see DOI: 10.1039/c5sc01053e



**DOI:** 10.1039/c5sc01053e

**Published:** 2015-04-20

**Authors:** Michele Bedin, Alavi Karim, Marcus Reitti, Anna-Carin C. Carlsson, Filip Topić, Mario Cetina, Fangfang Pan, Vaclav Havel, Fatima Al-Ameri, Vladimir Sindelar, Kari Rissanen, Jürgen Gräfenstein, Máté Erdélyi

**Affiliations:** a Department of Chemistry and Molecular Biology , University of Gothenburg , SE-412 96 Gothenburg , Sweden . Email: mate@chem.gu.se ; Tel: +46-31-786 9033; b University of Jyvaskyla , Department of Chemistry , Nanoscience Center , P.O. Box. 35, FI-40014 University of Jyvaskyla , Finland; c Department of Chemistry and RECETOX , Masaryk University , Kamenice 5 , 625 00 Brno , Czech Republic; d The Swedish NMR Centre , Medicinaregatan 5 , SE-413 90 Gothenburg , Sweden; e Department of Applied Chemistry , Faculty of Textile Technology , University of Zagreb , Prilaz baruna Filipovića 28a , HR-10000 Zagreb , Croatia

## Abstract

A detailed investigation of the influence of counterions on the [N–I–N]^+^ halogen bond in solution, in the solid state and *in silico* is presented. Translational diffusion coefficients indicate close attachment of counterions to the cationic, three-center halogen bond in dichloromethane solution. Isotopic perturbation of equilibrium NMR studies performed on isotopologue mixtures of regioselectively deuterated and nondeuterated analogues of the model system showed that the counterion is incapable of altering the symmetry of the [N–I–N]^+^ halogen bond. This symmetry remains even in the presence of an unfavorable geometric restraint. A high preference for the symmetric geometry was found also in the solid state by single crystal X-ray crystallography. Molecular systems encompassing weakly coordinating counterions behave similarly to the corresponding silver(i) centered coordination complexes. In contrast, systems possessing moderately or strongly coordinating anions show a distinctly different behavior. Such silver(i) complexes are converted into multi-coordinate geometries with strong Ag–O bonds, whereas the iodine centered systems remain linear and lack direct charge transfer interaction with the counterion, as verified by ^15^N NMR and DFT computation. This suggests that the [N–I–N]^+^ halogen bond may not be satisfactorily described in terms of a pure coordination bond typical of transition metal complexes, but as a secondary bond with a substantial charge-transfer character.

## Abstract 2

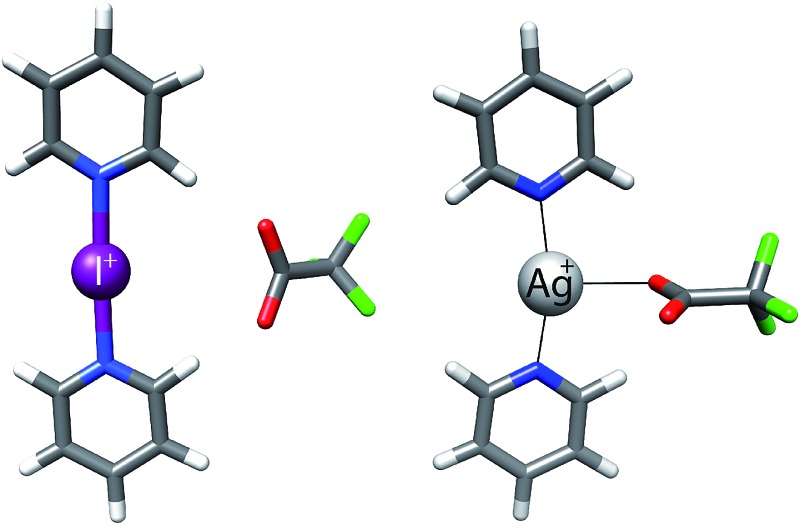
Counterions influence three-center halogen bonds differently than coordination bonds of transition metals.

## Introduction

Counterions play important roles in chemistry, for example in the modulation of organocatalysis,^1,2^ chemical reactions' energy profiles,^3,4^ metal cluster aromaticity,^5^ spectroscopic properties of organic compounds,^6^ surfactant micellization,^7^ membrane permeation,^8^ gelation,^9^ supramolecular assemblies,^10–12^ crystal structure,^13^ and chromatographic separation.^14^ A conceivable influence of counterions on the strength and geometry of halogen bonding has, however, so far barely received any attention.^15–17^ Halogen bonding has recently been defined,^18^ and has attracted vast interest due to its immense potential to provide a novel tool for supramolecular chemistry,^19,20^ crystal engineering,^21,22^ anion recognition,^23,24^ organic synthesis,^25^ structural biology,^26^ medicinal chemistry and chemical biology,^27^ for instance.

Three-center halogen bonds,^15^ sometimes also termed coordinative halogen bonds,^28,29^ are formed by the simultaneous interaction of an electrophilic halogen (X) with two electron donor functionalities (D). The three interacting atoms may be arranged in an asymmetric [D–X···D]^+^, or in a symmetric [D···X···D]^+^ geometry. Of these, the former is best described by a double well, whereas the latter by a single well energy potential.^30^ The asymmetric arrangement that encompasses a shorter and stronger covalent bond (D^*δ*+^–X), and a weaker and longer halogen bond (D^*δ*–^···X) is more polar than the one possessing a symmetric geometry and, accordingly, a symmetric charge distribution (D^*δ*–^···X^*δ*+^···^*δ*–^D). Strong, asymmetric coordination of a counterion may favor the formation of the asymmetric, more polar geometry over the symmetric, less polar one. The asymmetry of the analogous three-center [N–H–N]^+^ hydrogen bond^31^ has been partly attributed to the polarizing effect of the counterion.^32^ Limbach *et al.* reported the size and charge delocalization dependent effect of counterions on the symmetry of low barrier hydrogen bonds.^33^ Whether a counterion is capable of desymmetrizing the [N–I–N]^+^ halogen bond depends on the intrinsic energetic preference of the halogen bond for the symmetric geometry^30^ as well as on the properties of the counterion.

Early solid state ^14^N nuclear quadrupole resonance (NQR) and infrared studies suggested^34^ that the N–Br bonds of [bis(pyridine)bromine]^+^ are equivalent for its hexafluorophosphate salt, but nonequivalent for its perchlorate and tribromide salts. The loss of centrosymmetry in the latter two may be interpreted as a consequence of crystal packing forces in combination with the different coordination strength of various counterions. Alternatively, it may be explained by the highly comparable energies of the symmetric and the asymmetric geometries. However, both interpretations are debatable. Different N–Br bond lengths, 2.120(3) Å and 2.156(3) Å, were reported for the analogous [bis(quinuclidine)bromine]^+^ tetrafluoroborate complex in the solid state.^35^ For the analogous iodine centered pyridine salts no analogous counterion effects have yet been reported. Such [N–X–N]^+^ complexes are common synthetic reagents,^36–41^ whose reactivity is remarkably sensitive to minute structural^42^ and environmental changes.^41^ In contrast to the detailed knowledge collected on the influence of environmental factors on the closely related three-center hydrogen bonds,^32^ similar effects on halogen bonds have so far been assessed to a much lesser extent. As part of our ongoing investigation of the three-center halogen bond,^30,43^ the influence of the counterion on the strength and geometry of the interaction in solution and in the solid state is addressed herein.

## Results and discussion

**Fig. 1:**
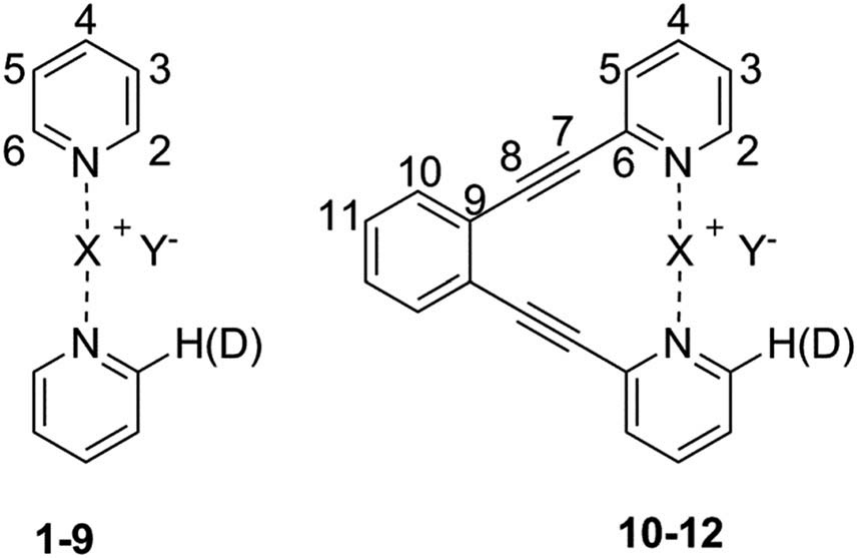
The structure of the model systems assessed for elucidation of the counterion (Y^–^) effect on the [N–I–N]^+^ halogen bond (X = I): [bis(pyridine)iodine]^+^ BF_4_^–^ (**1-I**), ClO_4_^–^ (**2-I**), PF_6_^–^ (**3-I**), SbF_6_^–^ (**4-I**), TfO^–^ (**5-I**), TsO^–^ (**6-I**), NO_3_^–^ (**7-I**), and CF_3_CO_2_^–^ (**8-I**), and their geometrically restrained [(1,2-bis(pyridin-2-ylethynyl)benzene)iodine]^+^ BF_4_^–^ (**10-I**), TfO^–^ (**11-I**) and NO_3_^–^ (**12-I**) analogues. The BF_4_^–^ of **9-I** was scavenged with dodecabenzylbambus[6]uril (Bn_12_BU[6]).^47^ The spectroscopic data of **1-I** to **8-I** were compared to those of the corresponding silver(i) complexes (X = Ag) **1-Ag** to **8-Ag**.

[Bis(pyridine)iodine]^+^ complexes^44^ and their structurally closely related, geometrically restrained 1,2-bis(pyridin-2-ylethynyl) benzene analogues provide versatile models for the investigation of the structure and properties of three-center halogen bonds,^15,30,38,39,43,45,46^ and were therefore utilized in this study. In order to evaluate the counterion's ability to modulate the geometry of the iodine centered, three-center halogen bond, a series of anions of varying size, charge distribution and coordination strength were explored. Besides the spherical, weakly coordinating anions^12^ BF_4_^–^, ClO_4_^–^, and PF_6_^–^ that have previously been suggested^34,35^ to influence the geometry of the related [bis(pyridine)bromine]^+^ complex, the weakly coordinating SbF_6_^–^, the moderately coordinating TfO^–^ and TsO^–^, and the small and strongly coordinating^10,12^ NO_3_^–^ and CF_3_CO_2_^–^ were assessed (Fig. 1). To ensure tight coordination,^45^ dichloromethane was chosen as solvent for the solution studies. By scavenging the counterion with the bambusuril Bn_12_BU[6],^47,48^ the “counterion-free” [N–I–N]^+^ complex was also investigated. As the three-center halogen bond was proposed to be essentially analogous to the coordination bond of silver(i) complexes,^28,29^ and as iodine(i) resembles silver(i) in its ionic radius (I^+^: 1.33 Å, Ag^+^: 1.29 Å) and in the linear geometry of its bis-coordination complex, we discuss the properties of the [N–I–N]^+^ complexes **1-I** to **8-I** in comparison to the corresponding [N–Ag–N]^+^ species **1-Ag** to **8-Ag** (Fig. 1).

### Synthesis

[Bis(pyridine)iodine]^+^ tetrafluoroborate (**1-I**), perchlorate (**2-I**), hexafluorophosphate (**3-I**), hexafluoroantimonate (**4-I**), triflate (**5-I**), tosylate (**6-I**), nitrate (**7-I**), and trifluoroacetate (**8-I**) complexes (Fig. 1), as well as their selectively deuterated analogues (**1-I-*d*** to **8-I-*d***), were synthesized following a previously published procedure^30,46^ from the corresponding silver(i) complexes (**1-Ag** to **8-Ag**, **1-Ag-*d*** to **8-Ag-*d***). Complex **9** was prepared by addition of 1.8 equivalents of the anion scavenger^47,48^ dodecabenzylbambus[6]uril (Bn_12_BU[6]) to the dichloromethane solution of **1-I**, to produce a “naked” [bis(pyridine)iodine]^+^ complex by trapping the BF_4_^–^ counterion. For the preparation of [(1,2-bis(pyridin-2-ylethynyl)benzene)iodine]^+^ tetrafluoroborate (**10**), triflate (**11**), and nitrate (**12**) and their mono-deuterated analogues (**10-*d*** to **12-*d***), published synthetic routes^30^ were followed. See ESI† for details.

### Counterion coordination in solution

Ion coordination in solution was evaluated by acquiring the translational diffusion coefficient of the positively and negatively charged species of complexes **1–9** (Table 1), using ^1^H and ^19^F NMR. Tight anion coordination to [bis(pyridine)iodine]^+^ was indicated by the comparable translational diffusion rates of the two ions in the same solution, for each studied system. A significant difference between the diffusion coefficients of the anionic and cationic species was detected for **9-I**. This indicates that [bis(pyridine)iodine]^+^ and BF_4_^–^ of **9-I** do not form a tight ion pair. In line with our expectations, the diffusion rate of the trapped BF_4_^–^ of **9-I** is lower than that of **1-I**, and matches the diffusion rate of the anion scavenging agent Bn_12_BU[6] (*D* = 5.8 × 10^–10^ m^2^ s^–1^) that was added to the solution. The slower diffusion rate of [bis(pyridine)iodine]^+^ of **9-I** than that of **1-I** is well explained by the increased solvation of charged species as compared to neutral ones in apolar solvents,^49^ and by an increased viscosity of the solution upon addition of Bn_12_BU[6]. Diffusion NMR reveals strong coordination of the counterions of the silver centered complexes **1-Ag** to **8-Ag** as well.

**Table 1:** Translational diffusion coefficients, measured by ^1^H and ^19^F NMR detection, and ^15^N NMR chemical shifts

Anion	Structure	*D* (cation)^*a*^ × 10^–10^ (m^2^ s^–1^)	*D* (anion) × 10^–10^ (m^2^ s^–1^)	*δ* (^15^N) (ppm)
BF_4_^–^	**1-I**	16.8	16.4	–175.1
ClO_4_^–^	**2-I**	9.5	—^*c*^	–175.0
PF_6_^–^	**3-I**	10.3	10.5	–175.8
SbF_6_^–^	**4-I**	9.8	—^*d*^	–175.1
TfO^–^	**5-I**	14.0	15.0	–175.1
TsO^–^	**6-I**	16.3	16.2	–174.8
NO_3_^–^	**7-I**	10.8	—^*c*^	–174.8
CF_3_CO_2_^–^	**8-I**	18.7	15.7	–175.2
[BF_4_^–^]^*b*^	**9-I**	9.4	6.0	–175.5
BF_4_^–^	**1-Ag**	9.3	9.9	–126.5
ClO_4_^–^	**2-Ag**	10.7	—^*c*^	–124.0
PF_6_^–^	**3-Ag**	9.5	9.4	–128.4
SbF_6_^–^	**4-Ag**	15.1	—^*d*^	–129.3
TfO^–^	**5-Ag**	13.1	13.0	–122.0
TsO^–^	**6-Ag**	9.4	8.5	–111.8
NO_3_^–^	**7-Ag**	10.4	—^*c*^	–113.1
CF_3_CO_2_^–^	**8-Ag**	15.2	12.9	–109.5

^*a*^[Bis(pyridine)iodine]^+^.

^*b*^BF_4_^–^ was scavenged with Bn_12_BU[6] in this solution providing a naked [bis(pyridine)iodine]^+^.

^*c*^This anion lacks an NMR active nucleus preventing the acquisition of its diffusion coefficient.

^*d*^The ^19^F NMR signal of this counterion is extensively split and broadened due to ^1^ *J*_Sb,F_.

^15^N NMR is a sensitive tool for investigation of halogen bond formation,^30,43,45,50^ and is commonly used for the assessment of solvent effects, protonation and metal coordination of nitrogenous Lewis bases.^51–53^ The ^15^N NMR chemical shifts of compounds **1–9** were detected using ^1^H,^15^N-HMBC experiments,^54^ providing a single signal for each compound (Table 1). Formation of the [N–I–N]^+^ complex is accompanied by a comparable, larger than 100 ppm chemical shift change (pyridine, *δ*_15N_ = –67 ppm (ref. 30)) for **1-I** to **9-I**. The variation of the ^15^N NMR chemical shifts of **1-Ag** to **8-Ag** indicates that, in contrast to iodine(i), silver(i) interacts with anions in a coordination-strength dependent manner. Solvent and counterion coordination to silver(i) complexes has previously been reported^52^ to have a major influence on pyridine ^15^N NMR coordination shifts (*δ*_coord_ = *δ*_complex_ – *δ*_ligand_). Strongly coordinating counterions may convert the linear, bis-coordinate [bis(pyridine)silver]^+^ to tris-coordinate, neutral [bis(pyridine)(counterion)silver] complexes.^52,55^ The complex **8-Ag** shows the most deshielded ^15^N NMR chemical shift among the silver(i) complexes, which is in line with CF_3_CO_2_^–^ being the strongest coordinating anion of the series. A smaller absolute coordination shift, 41–45 ppm, of the silver(i) complexes possessing strongly coordinating counterions, such as TsO^–^, CF_3_CO_2_^–^ and NO_3_^–^, is explained by an efficient charge transfer from the anion to silver(i) through orbital overlap. This decreases the positive charge of silver(i) and consequently the charge transfer from the pyridine nitrogens to silver(i), yielding smaller ^15^N NMR coordination shifts. The small, ≤1 ppm, ^15^N chemical shift variation of **1-I** to **9-I** indicates the absence of an analogous direct charge transfer interaction of the counterion to the iodine(i) of [bis(pyridine)iodine]^+^. Hence, strong direct coordination of a counterion to iodine(i) by orbital overlap does not take place; rather, this interaction remains electrostatic. Slightly lower ^15^N NMR chemical shifts were observed for **10-I** (–165.5 ppm), **11-I** (–165.0 ppm) and **12-I** (–163.3 ppm), reflecting weakened N–I bonds, as a consequence of the steric restraint introduced by the 1,2-diethynylbenzene backbone.^28^

The propensity of the counterions of **1-I** to **9-I** to compete with pyridine for coordination to the empty p-orbital of I^+^ was evaluated by analyses of the ^15^N NMR chemical shifts of the [bis(pyridine)iodine]^+^ counterion and of the alternative [(pyridine)(counterion)iodine] pyridine geometries, predicted at the B3LYP level using the dichloromethane continuum solvent model (Table 2, see ESI† for details). The ^15^N chemical shifts of the [bis(pyridine)iodine]^+^ counterion geometries of **1-I** to **8-I** (*N*_B_, Table 2) are predicted to be virtually counterion independent, and comparable in magnitude to those experimentally observed (Table 1). In contrast, considerably counterion dependent shifts are predicted for the [(pyridine)(counterion)iodine] complexes (*N*_A_, Table 2), with higher shielding of the nitrogen of those encompassing weaker coordinating anions. This suggests that pyridine *versus* anion exchange does not take place. Computational thermochemical analysis predicts that formation of a [(pyridine)(counterion)iodine] complex is endothermic for complexes that possess weakly and moderately coordinating anions, yet somewhat exothermic for TsO^–^, NO_3_^–^, and CF_3_CO_2_^–^ (Table 2). It should be underlined that the applied thermochemical calculation does not take into account the electrostatic attraction that arises in the electrolyte formed by [bis(pyridine)iodine]^+^ and its counterion in the solution. This attraction, which is also supposed to account for the variation in the experimental ^15^N shifts for **1-I** to **8-I**, is estimated to be 20–30 kJ mol^–1^ (see ESI† for details). With this correction, the DFT results are compatible with the [bis(pyridine)iodine]^+^ complex, being stable for **1-I** to **8-I**. Strong ion pairing is supported by the diffusion NMR data. This conclusion is further supported by detection of the [bis(pyridine)iodine]^+^ complex by HR(ESI)MS for all studied systems (see ESI for details†), further corroborating that pyridine *versus* anion exchange does not take place in solution.

**Table 2:** Computationally predicted ^15^N NMR chemical shifts of the (A) [(pyridine)(counterion)iodine] pyridine and of the alternative (B) [bis(pyridine)iodine(counterion)] geometries of **1-I** to **8-I**, and the estimated energies for (1) the hypothetical reaction of a pyridine-counterion exchange, and (2) the formation of the [bis(pyridine)iodine(counterion)] ion pair^*a*^

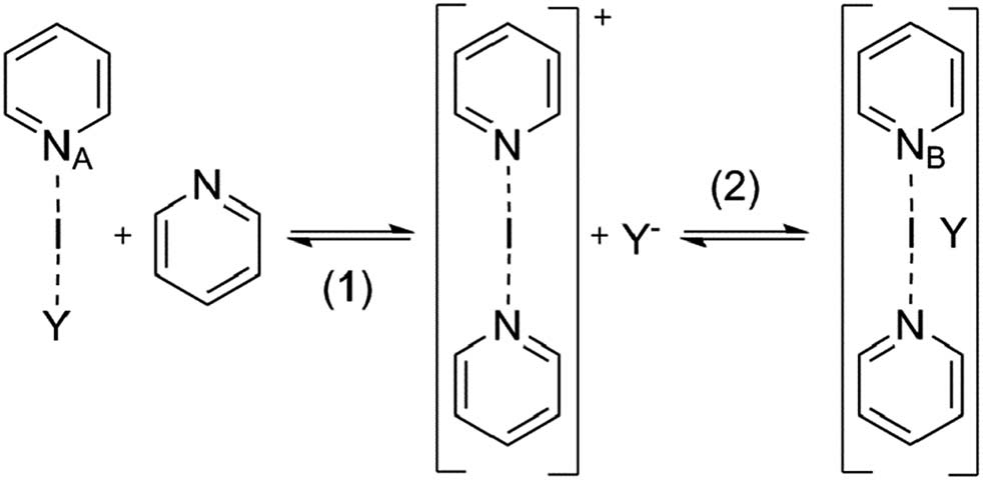					
Anion	Structure	*δ* (^15^N) ppm		Δ*G* kJ mol^–1^	
		*N*_A_	*N*_B_	(1)	(2)
BF_4_^–^	**1-I**	–222.6	–173.7	28.9	5.4
ClO_4_^–^	**2-I**	–201.5	–173.9	18.5	6.2
PF_6_^–^	**3-I**	–233.6	–174.3	44.2	19.7
SbF_6_^–^	**4-I**	–233.9	–174.6	39.7	3.9
TfO^–^	**5-I**	–198.7	–173.6	12.6	9.4
TsO^–^	**6-I**	–182.2	–175.2	–6.1	37.7
NO_3_^–^	**7-I**	–180.3	–172.7	–16.4	4.9
CF_3_CO_2_^–^	**8-I**	–176.1	–173.2	–25.7	40.4

^*a*^The experimental ^15^N NMR chemical shift of **9-I** was used as secondary reference.

**Fig. 2:**
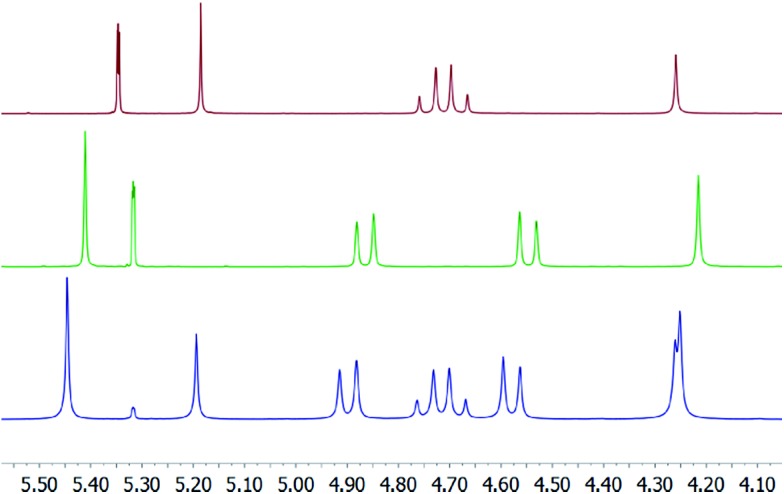
The aliphatic region of the ^1^H NMR of dodecabenzylbambus[6]uril (Bn_12_BU[6]) in the absence and presence of complex **1/1-*d***. *Top row (red)*: the spectrum of free Bn_12_BU[6]. *Middle row (green)*: the spectrum following addition of 1 eq. [bis(pyridine)iodine]^+^ tetrafluoroborate to the solution. The counterion is trapped as [(Bn_12_BU[6])(BF_4_^–^)]. *Bottom row (blue)*: the spectrum of Bn_12_BU[6] with 0.55 eq. [bis(pyridine)iodine]^+^ tetrafluoroborate. Signals belonging to the BF_4_^–^ complexed and the free Bn_12_BU[6] are observed simultaneously, indicating strong BF_4_^–^ binding. Hence, the use of an excess of Bn_12_BU[6] ensures the complete trapping of the anion from the dichloromethane solution.

The ^15^N NMR shift of **9-I** is comparable to that of **1-I**, suggesting that the presence or absence of a weakly coordinating counterion does not influence the electron distribution of the [N–I–N]^+^ halogen bond. The BF_4_^–^ of **9-I** was scavenged by addition of an excess (1.8 eq.) of Bn_12_BU[6] to the solution of **1-I** with the efficient trapping of the counterion being confirmed by characteristic changes (a) in the ^1^H NMR chemical shifts of bambusuril^47^ (Fig. 2), (b) of the translational diffusion coefficients of BF_4_^–^ (Table 1), (c) of the ^15^N NMR shifts alteration of Bn_12_BU[6] (Δ*δ*_15N_ = 2.1 ppm for N–CH_3_, and 2.4 ppm for N–CH_2_, Fig. S1†), and (d) by observation of intermolecular ^1^H,^19^F heteronuclear Overhauser effects between BF_4_^–^ and Bn_12_BU[6] (Fig. S2†) whereas there is no ^1^H,^1^H NOESY crosspeak between [bis(pyridine)iodine]^+^ and Bn_12_BU[6]. The use of an excess of Bn_12_BU[6] in the studies of **9-I** ensured the absence of free BF_4_^–^ in solution, and hence that a counterion-free [bis(pyridine)iodine]^+^ complex was investigated. EXSY crosspeaks between the signals of the free and BF_4_^–^-bound Bn_12_BU[6] (Fig. S3†) indicated anion migration between the Bn_12_BU[6] units. Upon addition of Bn_12_BU[6] to **8-I**, the ^15^N NMR chemical shift remained unaltered, supporting the above conclusions, and in addition further confirmed that CF_3_CO_2_^–^ is incapable of competing with pyridine for iodine(i) coordination.

### Halogen bond symmetry in solution

Isotopic perturbation of equilibrium (IPE) NMR^56^ is a technique capable of distinguishing between a static, symmetric geometry and a rapidly interconverting pair of asymmetric geometries in solution. It has been used successfully, for example, in the symmetry elucidation of [N–H···N]^+^ hydrogen bonded^57,58^ and [N···X···N]^+^ halogen bonded^30,46^ molecular systems, as well as of carbocations^59^ and metal chelating complexes.^60^ The main advantage of IPE is that it succeeds even when the rapidly interconverting species cannot be “frozen out” due to a shallow energy barrier between them. It requires the analysis of an isotopologue mixture of a compound, which is often a mixture of its selectively deuterated and nondeuterated analogues. In such solutions, two sets of NMR signals are observed due to the secondary isotope effect on the vibrational frequencies.^61^ The chemical shift difference of the signal originating from the deuterated and that of the nondeuterated molecule, most commonly detected at ^13^C NMR frequency, is the secondary isotope effect (IE) (eqn (1)),

1 ^*n*^*Δ*_obs_ = *δ*C_(D)_ – *δ*C_(H)_

where *n* is the number of bonds between the deuterium and the observed nucleus, whilst C_(D)_ and C_(H)_ are the ^13^C NMR chemical shifts of the deuterated and nondeuterated isotopologues. Although the magnitude of the isotope effect is dependent on the distance of the detected nucleus from the position of isotopic substitution, a sizeable ^*n*^ *Δ*_eq._ need not be restricted to a small *n*. The observed isotope effect, ^*n*^ *Δ*_obs_, consists of intrinsic, ^*n*^ *Δ*_0_, and equilibrium, ^*n*^ *Δ*_eq._, isotope effects (eqn (2)).

2 ^*n*^*Δ*_obs_ = ^*n*^*Δ*_0_ + ^*n*^*Δ*_eq._

The first component, ^*n*^ *Δ*_0_, is the direct consequence of isotope substitution, and is essentially temperature independent. The second component, ^*n*^ *Δ*_eq._, manifests only in systems that are involved in a dynamic exchange process. Due to its dependence on the equilibrium constant of an exchange process (eqn (3)), ^*n*^ *Δ*_eq._ is temperature dependent.

3 ^*n*^*Δ*_eq._ = *D*(*K* – 1)/[2(*K* + 1)]

where *K* is the equilibrium constant and *D* is the chemical shift difference of the exchanging species, in this study the chemical shift difference of a selected nucleus in the N^*δ*+^–I, and the N^*δ*–^···I^*δ*+^ forms of pyridine. Accordingly, static ([N···I···N]^+^) and dynamic ([N–I···N]^+^ ⇄ [N···I–N]^+^) halogen bonding geometries are distinguishable in solution by observation of the temperature dependence of their ^2^H-induced ^13^C NMR isotope effects.^30,56^

**Fig. 3:**
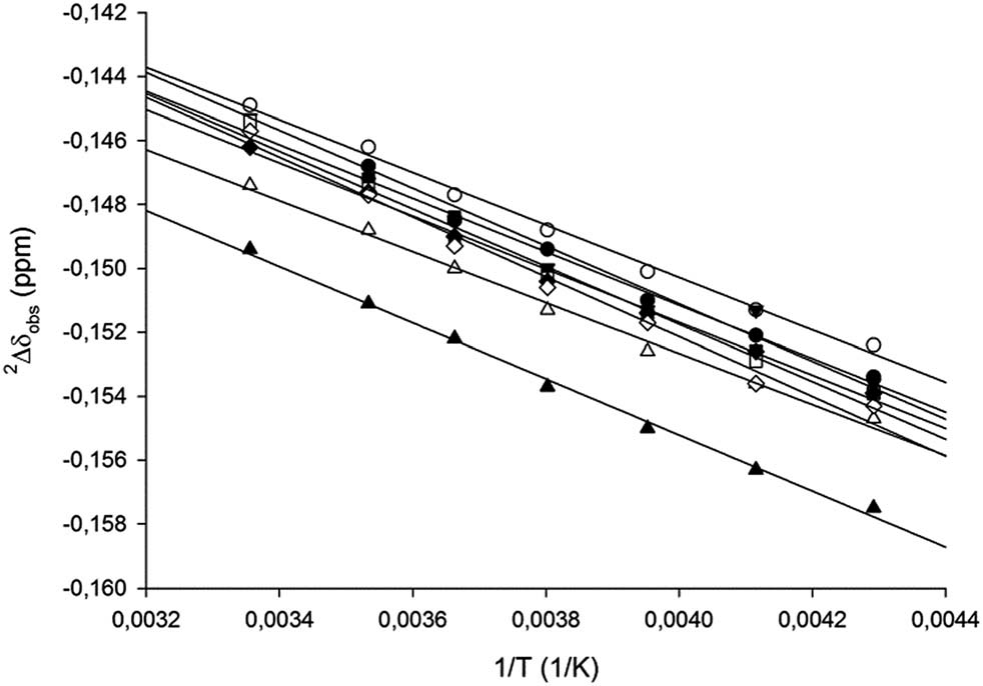
The similar temperature dependence of the two-bond isotope effect (^2^ *Δ*_obs_) measured on C-3 of **1-I** to **8-I** indicates that the [N–I–N]^+^ halogen bond is static and symmetric in solution, regardless of the counterion. ○ BF_4_^–^, ClO_4_^–^, ■ PF_6_^–^, □ SbF_6_^–^, TfO^–^, ♦ TsO^–^, △ NO_3_^–^, ▲ CF_3_CO_2_^–^, and BF_4_^–^.

We applied IPE to evaluate whether the geometry of the three-center [N–I–N]^+^ halogen bond of [bis(pyridine)iodine]^+^^14,30,46^ could be influenced in solution by counterion coordination. Previously this bond was demonstrated to be static and symmetric in solution in a complex encompassing TfO^–^ as counterion.^30^ By acquisition of ^13^C {^1^H,^2^H} NMR spectra of isotopologue mixtures of **1-I** to **9-I** dissolved in CD_2_Cl_2_ at various temperatures, the temperature dependence of the isotope shifts was determined.^62^ Similar to previous studies,^30,45,46,63^ the C3 position of the pyridine of **1-I** to **9-I** showed the largest temperature dependence and provided data with the highest squared correlation coefficients (Fig. 3). The overall smaller temperature dependence of the IEs of the studied [bis(pyridine)iodine]^+^ complexes (Table 3) as compared to that of pyridine pyridinium triflate (**5-H**), which was previously shown to exist as a rapidly interconverting tautomeric mixture,^30,45,46^ indicates that **1-I** to **9-I** are present as static, symmetric species in solution. Here it should be noted that the nonzero temperature dependence of the intrinsic isotope effects is due to the temperature dependence of the polarity of the solvent,^64^ the polarity-alteration of the solvent modulates the charge distribution of pyridine.^65^ An efficient electron density transfer between the nitrogen lone pair and adjacent bonds, influencing the magnitude of the observed isotope effects as well as the amine basicity, has been previously described.^66^ Through-space polarization *via* dipolar interaction has also been reported previously for ethers, for example.^67^ The static symmetric [N···I···N]^+^ geometry of **1-I** to **9-I** is corroborated by the primary dependency of the magnitude of their IEs on the distance of the observed carbon from the position of ^1^H-to-^2^H substitution, in contrast to those of [bis(pyridine)hydrogen]^+^ triflate **5-H** (Table 3) being predominantly dependent on the number of intervening bonds between the observed nucleus and the nitrogen involved into the [N–H···N]^+^ ⇄ [N···H–N]^+^ tautomerization process.^31,68^ Similar to **1-I**, **9-I** has a static, symmetric [N···I···N]^+^ halogen bond in solution, confirming that a weakly coordinating anion does not influence the symmetry of the iodine centered halogen bond. The above observations agree with the previously reported^15,30,43,46^ high energetic gain upon formation of a symmetric [N···I···N]^+^ halogen bond. Similar to others,^13^ we have observed a lower stability of **5-I**, manifested in its shorter lifetime in solution as compared to [bis(pyridine)iodine]^+^ complexes that encompass other counterions. This difference is likely due to the hygroscopicity of triflic acid and not due to any specific interaction of TfO^–^ with the [N···I···N]^+^ halogen bond. Counterion dependent stability and reactivity of [bis(pyridine)halogen]^+^ complexes may influence their synthetic applicability,^36,41,69^ encouraging for further studies.

**Table 3:** Temperature coefficients (ppm K) of the isotope shifts of **1–9**, observed for CD_2_Cl_2_ solutions

Anion	Structure	C2 ^1^ *Δ*_obs_	C3 ^2^ *Δ*_obs_	C4 ^3^ *Δ*_obs_	C5 ^4^ *Δ*_obs_	C6 ^3^ *Δ*_obs_
BF_4_^–^	**1-I**	–6.6	–8.4	0.3	0	–2.9
ClO_4_^–^	**2-I**	–5.6	–8.3	0.3	0	–3.6
PF_6_^–^	**3-I**	–6.3	–8.9	0.4	0	–3.4
SbF_6_^–^	**4-I**	–6.2	–9.0	0.8	0	–3.4
TfO^–^	**5-I**	–5.9	–8.5	0.2	0	–3.8
TfO^–^	**5-H** ^*b*^	–6.1	–9.8	–4.5	–5.8	–6.5
TsO^–^	**6-I**	–2.6	–8.1	–0.3	0	–3.2
NO_3_^–^	**7-I**	–5.8	–8.0	0.9	0	–2.5
CF_3_CO_2_^–^	**8-I**	–6.3	–8.8	0.9	0	–4.6
[BF_4_^–^]	**9-I** ^*a*^	–6.5	–9.4	0.6	0	–3.5
BF_4_^–^	**10-I**	–6.5	–9.4	n.d.^*c*^	0	n.d.^*c*^
TfO^–^	**11-I** ^*b*^	–7.4	–6.5	–2.4	0	–2.7
TfO^–^	**11-H** ^*b*^	–10.0	–10.6	–3.5	0	+15.0
NO_3_^–^	**12-I**	n.d.^*c*^	–9.8	2.5	0	–4.2

^*a*^The counterion of **9-I** was scavenged using Bn_12_BU[6] providing a naked [bis(pyridine)iodine]^+^.^47^

^*b*^The data of **5-H**, **11-I** and **11-H** are from ref. 30 and 46.

^*c*^Due to limited solubility and minor temperature dependence, this coefficient could not be reliably determined.

The three-center halogen bond is slightly destabilized by the introduction of a geometrical restraint enforcing a somewhat longer than optimal nitrogen–nitrogen distance for the [N···I···N]^+^ bond, thereby decreasing the overlap of the filled nonbonding orbital of the pyridine nitrogens with the empty p-orbital of iodine(i).^30,45^ Consequently, optimal N–I bond lengths are obtainable for such a system only upon an energetically penalized adjustment of its covalent backbone. To evaluate whether the counterion is capable of influencing a weakened three-center halogen bond, [(1,2-bis(pyridin-2-ylethynyl)benzene)iodine]^+^ complexes (Fig. 1) encompassing the weakly coordinating tetrafluoroborate (**10-I**), the moderately coordinating triflate (**11-I**), and the strongly coordinating nitrate (**12-I**) were assessed using IPE NMR. The overall lower temperature dependence of the IEs of **10-I** to **12-I** as compared to **11-H** (Table 3), and the primary dependence of the temperature coefficients of their IEs on the position of the ^1^H-to-^2^H substitution indicate that the [N···I···N]^+^ halogen bond of these systems is also static and symmetric. Hence, counterion coordination is incapable of introducing asymmetry into even a slightly weakened three-center halogen bond in solution.

### Counterion coordination *in silico*

**Fig. 4:**
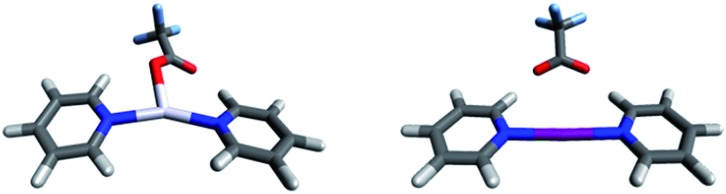
The DFT-predicted equilibrium geometries of complexes **8-Ag** and **8-I** are shown on the left and right, respectively. Whereas silver(i) is predicted to form T-shaped, tris-coordinate species with a strongly bound counterion, short iodine–oxygen contact is not seen for the corresponding iodine(i) complex, which prefers a linear, bis-coordinate N–I–N geometry.

For theoretical confirmation of the experimental findings, the equilibrium geometries of **1-I** to **9-I** were calculated using density functional theory (DFT) employing the B3LYP exchange and correlation functional.^70–73^ In the thermochemical calculations, the LANL08d and LANL08f^74^ basis sets, in conjunction with the LANL2DZ^75,76^ effective core potential, were used for I and Sb, respectively; the LANL2DZ^75,76^ basis set was used for Ag; Pople's 6-311+G(d,p)^77–79^ basis set was used for B, O, N, F and Cl; and Pople's 6-311G(d,p) basis set was used for the remaining atoms. For the estimation of chemical shieldings, single-point calculations at the geometries obtained were performed using the 6-311+G(d,p) basis set^80^ for I, and Kutzelnigg's IGLO-III basis set^81^ for the remaining atoms. Solvent effects were accounted for by the Polarizable Continuum Model (PCM),^82,83^ with CH_2_Cl_2_ as the solvent. All calculations were performed using the Gaussian09 program package.^84^ The potential energy surfaces (PES) for all the complexes containing counterions are complicated and likely to show a number of local minima for the placement and orientation of the counterion. Care was taken to find the most favourable configuration for each counterion; however, it can never be fully excluded that there are local minima with slightly lower energies than those presented. The PESs are shallow in the region of interest, so even if a slightly lower minimum geometry was missed this would not affect the conclusions of the thermochemical analysis, shown in Table 2. In good agreement with the spectroscopic data, DFT calculations predict symmetric [bis(pyridine)iodine]^+^ geometry for complexes **1-I** to **9-I**. The minor, <0.2%, difference in the N–I bond lengths of **8-I** (Table 4), for example, is likely insignificant.^85^ The N–I bond length is virtually unaffected by the counterion, whereas the N–I–N angle shows some minor variation, yet remains overall linear. The latter deviation from complete linearity is likely due to weak hydrogen bonding of some of the counterions to the H-2 of the pyridines. This interaction has previously been noticed for structurally closely related complexes.^52,86,87^ Most of the analogous silver complexes are also linear and symmetric; however, a slight asymmetry along with a significant distortion from linearity of the N–Ag–N angle is predicted for **5-Ag**, **7-Ag** and **8-Ag**. These complexes encompass small, moderately or strongly coordinating counterions (TfO^–^, NO_3_^–^ and CF_3_CO_2_^–^), and their geometry is likely altered due to the steric requirements of tight counterion coordination that yields neutral, T-shaped and slightly asymmetric species (Fig. 4). Neither small symmetric (BF_4_^–^, ClO_4_^–^, PF_6_^–^, SbF_6_^–^) nor larger sterically hindered anions (TsO^–^) are capable of direct coordination to silver(i) in these complexes. It should be noted that the corresponding iodine(i) centered complexes **7-I** and **8-I** do not form a comparable strong iodine–oxygen bond, and thus remain linear and bis-coordinate.

**Table 4:** Computationally predicted and X-ray crystallographically determined N–X bond distances and N–X–N bond angles for the complexes **1-I** to **9-I** and **1-Ag** to **8-Ag**

Anion	Structure	Computationally predicted distances and angles			X-ray crystallographic distances and angles		
		*r*(N–X)_1_ (Å)	*r*(N–X)_2_ (Å)	*σ*(N–X–N) (°)	*r*(N–X)_1_ (Å)	*r*(N–X)_2_ (Å)	*σ*(N–X–N) (°)
BF_4_^–^	**1-I** ^*a*^	2.301	2.301	178.0	2.260(3) × 2	^*c*^	180.0
					2.261(3) × 2	^*c*^	180.0
					2.255(3)	2.261(3)	177.7(1)
ClO_4_^–^	**2-I**	2.301	2.301	175.8	2.257(2) × 2	^*c*^	180.0
					2.260(2) × 2	^*c*^	180.0
					2.256(2)	2.256(2)	177.72(9)
PF_6_^–^	**3-I**	2.303	2.301	178.8	2.268(2)	2.268(2)	180.0
SbF_6_^–^	**4-I**	2.302	2.302	179.2	2.252(3)	2.252(3)	180.0
TfO^–^	**5-I**	2.301	2.300	178.0	2.246(8)	2.261(7)	178.0(3)
TsO^–^	**6-I**	2.301	2.300	177.8	2.241(3)	2.268(3)	178.75(8)
NO_3_^–^	**7-I**	2.303	2.303	179.0	2.250(4) × 2	^*c*^	180.0
					2.265(3) × 2	^*c*^	180.0
CF_3_CO_2_^–^	**8-I**	2.302	2.298	177.4	—	—	—
[BF_4_^–^]	**9-I** ^*b*^	2.301	2.301	180.0	—	—	—
BF_4_^–^	**1-Ag**	2.198	2.197	175.7	2.137(3)	2.138(3)	176.0(1)
ClO_4_^–^	**2-Ag**	2.190	2.189	179.1	2.131(3)	2.132(3)	177.0(1)
PF_6_^–^	**3-Ag**	2.193	2.192	176.9	2.128(4)	2.133(4)	176.9(1)
SbF_6_^–^	**4-Ag**	2.193	2.192	178.0	2.130(2)	2.143(2)	174.84(8)
TfO^–^	**5-Ag**	2.225	2.222	166.8	2.153(4)	2.158(3)	167.4(1)
TsO^–^	**6-Ag**	2.191	2.188	176.8	2.177(2)	2.192(2)	155.01(7)
NO_3_^–^	**7-Ag**	2.251	2.251	162.5	2.176(2)	2.176(2)	173.85(8)
NO_3_^–^	**7-Ag-2**				2.152(2)	2.152(2)	173.2(1)
					2.255(3)^*d*^	—	—
CF_3_CO_2_^–^	**8-Ag**	2.274	2.250	164.5	—	—	—

^*a*^Crystallographic data is from ref. 89.

^*b*^Computational data is from ref. 30.

^*c*^Complex lies on a symmetry element with two equal N–I distances with an exact 180° angle.

^*d*^Only one Ag–N bond in a pseudo-octahedral complex.

### Counterion coordination in the solid-state

**Fig. 5:**
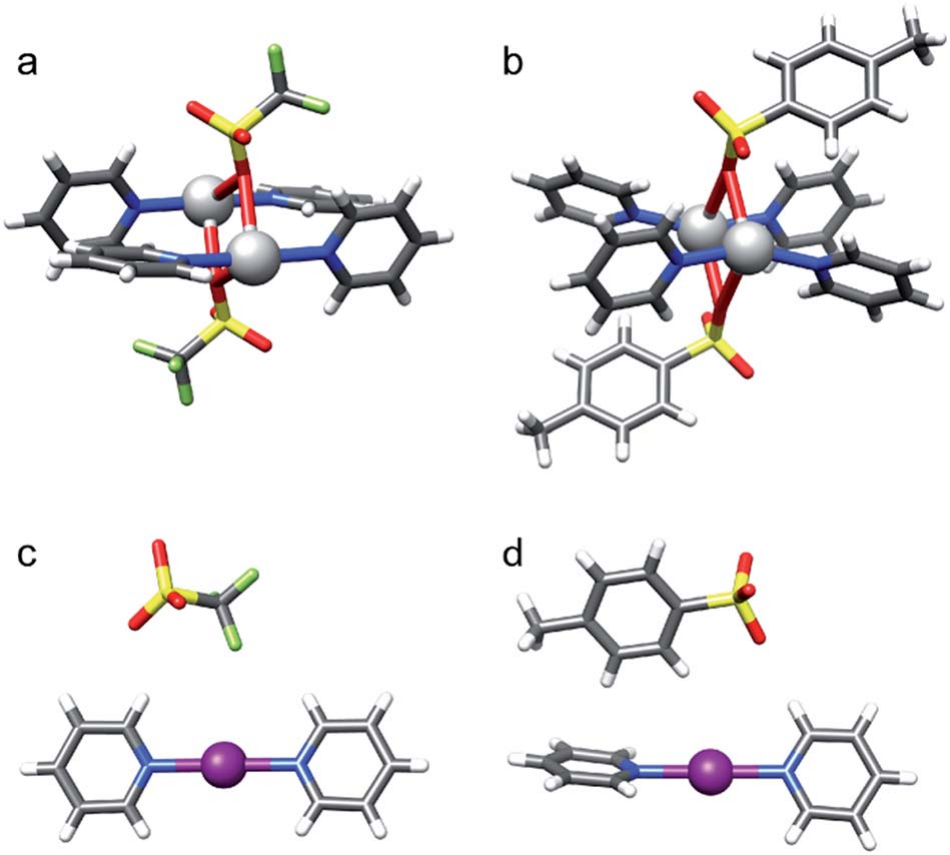
The solid state geometries of complexes (a) **5-Ag**, (b) **6-Ag**, (c) **5-I** and (d) **6-I** obtained by X-ray crystallography reveal that the bis(pyridine) complexes of silver(i) form pseudo-tetracoordinated dimers, whereas those of iodine(i) form ionic, linear, bis-coordinated N–I–N complexes in the presence of moderately coordinating counterions, such as TfO^–^ and TsO^–^.

Single crystals were obtained *via* slow diffusion of hexane into the dichloroethane solution of the studied complexes under stepwise cooling from 25 °C to –20 °C. X-ray crystallographic analyses verified that the iodine centered complexes **1-I** to **7-I** prefer a symmetric, linear, bis-coordinated N–I–N structure in the solid state (Table 4). The counterion has only a minor, <2%, influence on the N–I bond lengths. A weak coordination of the anions and their consequent negligible effect on the geometry of the three-centered halogen bond in the solid state is in good agreement with the DFT predicted stable, symmetric [bis(pyridine)iodine]^+^ solution geometries. The N–I bond lengths of these complexes are significantly shorter than the sum of the van der Waals radii of the participating atoms (*R*_XB_ = 0.64, where *R*_XB_ = *d*_NI_/(*I*_vdW_ + *N*_vdW_)^88^), well reflecting the unusual strength of the halogen bond^29^ of **1-I** to **7-I**. Importantly, the asymmetric coordination of the counterion (Fig. 5) of these complexes is unable to induce asymmetry in the three-center halogen bond. Whereas the [N···I···N]^+^ interaction has a significant covalent character,^15,28,30,43,45^ the coordination of the counterions to [bis(pyridine)iodine]^+^ is predominantly Coulombic and is weak. Unfortunately, single crystals for complex **8-I** were not obtained, despite repeated attempts.

**Fig. 6:**
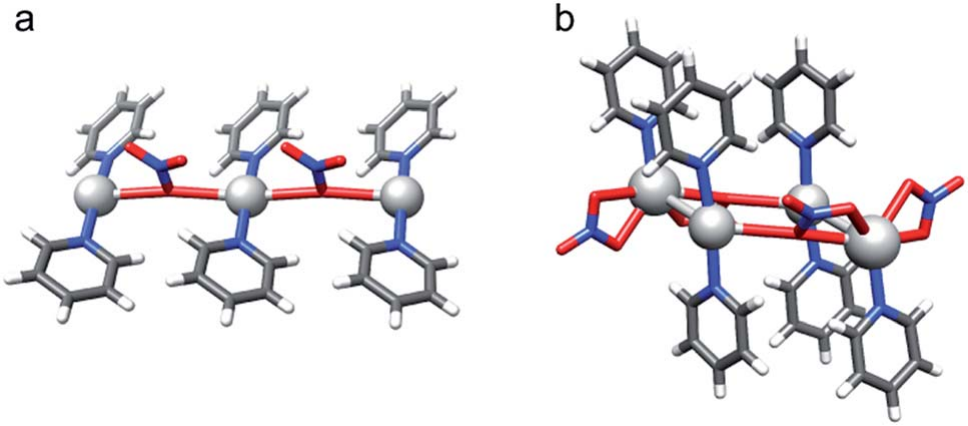
X-ray crystallographic investigation of [bis(pyridine)iodine]nitrate revealed (a) pseudo-square planar N–Ag(NO_3_^–^)_2_–N (**7-Ag**), and (b) coexisting pseudo-tetrahedral and pseudo-octahedral (5 + 1) N–(Ag)Ag(NO_3_^–^)_2_–N (**7-Ag-2**) coordination geometries.

Silver(i) centered complexes **1-Ag** to **4-Ag** that encompass weakly coordinating anions prefer linear, bis-coordinate N–Ag–N geometries in the solid state (Table 4). The interaction of [bis(pyridine)silver]^+^ with these counterions is of ionic character, similar to that of the corresponding [bis(pyridine)iodine]^+^. In contrast, pseudo-tetracoordinate (3 + 1) dimers were observed for **5-Ag** and **6-Ag** that possess the moderately coordinating counterions TfO^–^ and TsO^–^ (Fig. 5). X-ray analyses of [bis(pyridine)silver]^+^ nitrate showed a pseudo-square planar N–Ag(NO_3_^–^)_2_–N geometry (Fig. 6a, Table 4, **7-Ag**), and a tetramer (dimer of dimers) with an extraordinary combination of a pseudo-tetrahedral (2 + 2) N–(Ag)Ag(NO_3_^–^)–N coordination and an exceptional pseudo-octahedral (5 + 1) N–(Ag)Ag(NO_3_^–^)_2_–N coordination (Fig. 6b, Table 4, **7-Ag-2**). Despite repeated attempts, single crystals for the [bis(pyridine)silver]^+^ complex with the strongly coordinating counterion CF_3_CO_2_^–^ (**8-Ag**) were not obtained. X-ray analyses of **7-Ag** and **8-Ag** were attempted earlier by White *et al.*,^90^ who reported the formation of binuclear species containing silver nitrate and pyridine in a 1 : 3, and silver trifluoroacetate and pyridine in a 3 : 2, ratio. DFT predicts the tris-coordinate complexes of **7-Ag** and **8-Ag** to be energetically more favorable than their bis-coordinate analogues. The strong interaction of the counterion of complexes **5-Ag** to **8-Ag** with silver(i) manifests in specific, close interatomic contacts, *i.e. d*_Ag–O_ = 2.705 Å (**5-Ag**, XRD), 2.545 Å (**6-Ag**, XRD), 2.690 Å (**7-Ag**, DFT), and 2.749 Å (**8-Ag**, DFT). The counterion coordination to silver(i) in these species is strong (*R*_coord_ = 0.79–0.85, where *R*_coord_ = *d*_Ag–O_/(Ag_vdW_ + O_vdW_)^29,88^), yet it remains weaker as compared to the coordination of pyridines (*R*_coord_ = 0.67–0.69).

**Fig. 7:**
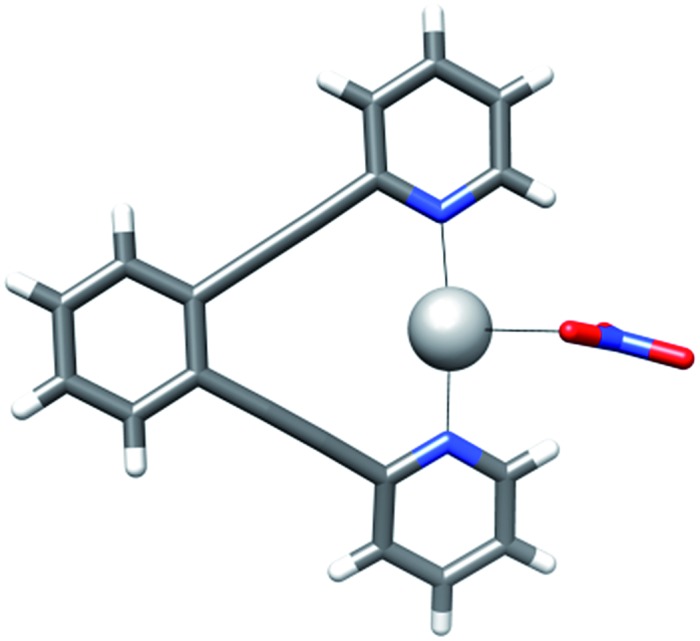
The X-ray structure of **12-Ag**.

[(1,2-Bis(pyridin-2-ylethynyl)benzene)silver]^+^ nitrate (**12-Ag**) crystallized as a T-shaped complex, as shown in Fig. 7. The N–Ag bond distances of 2.169(3) Å and 2.177(3) Å, and the Ag–O bond distance of 2.658(4) Å of this complex correspond well to those observed for the sterically unrestrained **7-Ag** (Table 4). The adaptability of the (1,2-bis(pyridin-2-ylethynyl)benzene) backbone of this complex to permit optimal nitrogen–nitrogen distances for complexation agrees well with previous observations made for the analogous [N···I···N]^+^ and [N···Br···N]^+^ complexes.^30^

## Conclusions

Counterions do not influence the intrinsically preferred linear, centrosymmetric geometry of the [N···I···N]^+^ halogen bond, either in solution or in the solid state. Neither does the scavenging of the counterion influence the symmetry of the three-center bond. In contrast, the weakly coordinating counterions BF_4_^–^ and ClO_4_^–^ were proposed^34,35^ to be capable of desymmetrizing the [N···Br···N]^+^ halogen bond of [bis(pyridine)bromine]^+^ in the solid state. This observation may be explained by the lower energy gain upon formation of the [N···Br···N]^+^ as compared to the [N···I···N]^+^ halogen bond.^43^ The three-center halogen bonds of **1-I** to **8-I** resemble the classical coordination bond of the corresponding [N···Ag···N]^+^ complexes.^28^ Whereas moderately and strongly coordinating counterions, *e.g.* TfO^–^, TsO^–^, NO_3_^–^ and CF_3_CO_2_^–^, do not affect the [N···I···N]^+^ halogen bond, they have a considerable influence on the corresponding coordinative silver(i) complexes. Hence, the latter are altered from linear, bis-coordinate [bis(pyridine)silver]^+^ counterion complexes to multi-coordinate [bis(pyridine)(counterion)silver] species. These counterions enforce nonlinear, and occasionally also asymmetric geometries, of the [N···Ag···N]^+^ coordination bonds. This is most apparent in the solid state (Table 4, Fig. 5 and 6), and yet it takes place in solution (Fig. 4) as well.

Overall, the three-center [N···I···N]^+^ and [N···Ag···N]^+^ bonds behave similarly in the presence of weakly coordinating anions (**1-I** to **4-I**, and **1-Ag** to **4-Ag**), but differently when moderately or strongly coordinating counterions are present (**5-I** to **8-I**, and **5-Ag** to **8-Ag**). Our results suggest that care should be taken when the three-center halogen bond is discussed in terms of coordination bonds of transition metals. The [N···I···N]^+^ bonds are best described as halogen bonds possessing a significant charge transfer character,^43^ which is present for all types of halogen bonds but yet may be of varying importance.^91–93^ Despite some differences,^15^ the three-center [D···X···D]^+^ halogen bond resembles a “short-strong” [D···H···D]^+^ hydrogen bond,^94^ and may alternatively be termed a short, strong halogen bond.

[Bis(pyridine)iodine]^+^ complexes are common synthetic reagents for halogenation and oxidation,^95–103^ with Barluenga's reagent being the most famous one.^36,41^ The latter was recently shown to exhibit a counterion dependent order–disorder phase transition in the solid state^13^ and has received increasing attention for applications in parallel synthesis^104^ and protein chemistry,^69^ for example. The understanding of the structure and properties of these reagents is therefore of both fundamental and practical importance.
