# Supplementary material for: Counterion influence on the N–I–N halogen bond
Source: Chem Sci. 2015 Apr 20;6(7):3746–56. doi: 10.1039/c5sc01053e (PMC5707496; doi:10.1039/c5sc01053e)
Supplement: Supplementary file 1 [file SC-006-C5SC01053E-s001.pdf]

Electronic Supplementary Material (ESI) for Chemical Science.
This journal is © The Royal Society of Chemistry 2015

Electronic Supplementary Information

Counterion Influence on the N-I-N Halogen Bond

Michele Bedin, Alavi Karim, Marcus Reitti, Anna-Carin C. Carlsson, Filip Topic, Mario Cetina, Pan Fangfang, Vaclav Havel, Fatima Al-Ameri, Vladimir Sindelar, Kari Rissanen, Jürgen Gräfenstein, and Máté Erdélyi

Table of Content

S2 1. Experimental section. General information, synthesis, compound characterization

S6 2. NMR spectra referred to in the main text

S9 3. NMR chemical shifts and their temperature dependence

S31 4. Computational details

S31 4.1 Cartesian coordinates, energies and selected vibrational frequencies.

S60 4.2 Thermochemistry

S62 4.3 Isotropic NMR chemical shieldings

S72 4.4 Estimation of the electrolytic stabilization energy

S73 5. Crystal structure determinations

S76 6. ^{1}H and ^{13}C NMR spectra of the studied compounds

S97 7. References

1. EXPERIMENTAL SECTION

Synthesis - General information. Unless otherwise stated, all reagents and solvents were obtained from commercial suppliers and used without further purification. CH_2Cl_2 was freshly distilled from CaH_2, and *n*-hexane was distilled from sodium metal/benzophenone ketyl in presence of tetraethylene glycol dimethyl ether prior to use. Reactions were carried out in capped Biotage microwave process vials. All glassware used in the synthesis of $[N\cdots X\cdots N]^+$ complexes was dried in an oven at 150 °C for several hours prior to use. The halogenation reactions were all performed under dry conditions with dry solvents, and in a nitrogen or an argon atmosphere. Centrifugation was carried out with a Heraeus Christ Labofuge A centrifuge. High resolution mass spectroscopy (HRMS) data was obtained on samples dissolved in dichloromethane using direct injection on a Q-TOF-MS with detection at 50-700 g/mol at Stenhagen Analyslab AB, Gothenburg, Sweden, with detection in the positive ion mode. For structural assignments ^{1}H NMR ^{13}C NMR, ^{19}F NMR, and ^{1}H,^{15}N NMR spectra were recorded on a Varian VNMR-S 500 or a Varian 400-MR spectrometer in CD_2Cl_2 at 25 °C. Chemical shifts are reported on the δ scale in ppm. For the ^{1}H and ^{13}C NMR spectral data the residual solvent signal was used as internal standard (δ_H 5.32 ppm and δ_C 54.00 ppm). For the ^{19}F NMR and ^{1}H,^{15}N gHMBCAD NMR spectral data sealed capillaries filled with hexafluorobenzene (δ_F -164.4 ppm) or nitromethane (δ_N 0.0 ppm) were used as external standards. The numbering of the structures refers to those used for NMR assignement For the syntheses of 1,2-bis(pyridin-2-ylethynyl)benzene, and its mono-deuterated analogue previously published routes were followed.[1] The syntheses of **11-I** and **11-I-*d*** are also previously reported.[1,2]

General synthetic protocol for [bis(pyridine)silver]$^+$ complexes. The Ag(I) salt (0.58 - 3.01 mmol, 1.0 eq.) was added to a 20-mL vial equipped with a stir bar. The vial was immediately sealed with a rubber septum. Subsequently, CH_2Cl_2 (4.0 mL) and pyridine (1.26 – 6.03 mmol, 2.0 eq.) were added *via* syringe at room temperature under nitrogen. The resulting mixture was stirred for 5-10 min, or until a clear mixture was obtained. Subsequently, *n*-hexane was added and a precipitate was formed. Centrifugation for 10 min at 3000 rpm, followed by removal of the supernatant by syringe, and drying under vacuum for several hours, furnished the $Py_2Ag(I)$ complex as a white solid in a moderate to high yield (52-96%).

General synthetic protocol for [bis(pyridine)iodine]$^+$ complexes. A 20-mL vial, sealed with a rubber septum, containing the [bis(pyridine)silver]$^+$ salt (0.55 - 1.96 mmol, 1.0 eq.), was dried under vacuum. Subsequently, CH_2Cl_2 (4.0 mL) and pyridine (1.11 – 3.92 mmol, 2.0 eq.) were added by syringe at room temperature under nitrogen atmosphere. The resulting mixture was three times alternatingly evacuated and flushed with nitrogen. Subsequently, I_2 (0.55 - 1.96 mmol, 1.0 eq.) was added, and the reaction mixture was stirred at ambient temperature under nitrogen for 5-10 min. Upon the addition of I_2, yellow AgI(s) was immediately precipitated. After centrifugation at 3000 rpm for 10 min, the clear, reddish supernatant containing the $Py_2I(I)$ complex was transferred *via* cannula into another sealed, 20-mL vial kept under vacuum. Following the transfer, the complex was precipitated by addition of *n*-hexane (20 mL). Precipitation was continued at -20 °C, in a freezer, for 30 min. The solid obtained after centrifugation at 3000 rpm for 10 min, and subsequent removal of the supernatant by syringe, was washed once with *n*-hexane (20 mL). Centrifugation, followed by removal of the supernatant as described above, and final drying under vacuum, furnished the $Py_2I(I)$ complex as a solid in a moderate to high yield (63-91%).

General crystallization procedure for [bis(pyridine)silver]$^+$ and [bis(pyridine)iodine]$^+$ complexes. The [bis(pyridine)silver]$^+$ or [bis(pyridine)iodine]$^+$ complex (50-150 mg) was dissolved in dichloroethane (2.0 mL) in a sealed, 4-mL vial. A small volume of hexane was added by syringe to generate a thin layer on the top of the dichloroethane solution. The vial was allowed to cool gradually, from room temperature to -8 °C, over 2 days to obtain crystals. Upon crystal formation, the vial was kept in the freezer at -20 °C ahead of X-ray crystallographic analysis.

General synthetic protocol for generation of a mixture of [bis(pyridine)iodine]$^+$ and selectively deuterium labelled [bis(pyridine)iodine]$^+$ complexes (1-I/1-I-*d* - 12-I/12-I-*d*) for isotopic perturbation of equilibrium studies. A 2:1 mixture of pyridine-2-d_1 and pyridine (1 mmol, 2 eq) and the silver(I) salt (0.5 mmol, 1 eq) with varying counterions were suspended in CH_2Cl_2 (5.0 mL) in a 20 mL vial sealed with a rubber septum equipped with a stir bar. The mixture was stirred at room temperature until all solids disappeared. Then, addition of *n*-hexanes leads to precipitation of the

[bis(pyridine)silver]$^+$ complexes as a white solid. The flask is then centrifuged, the supernatant removed and the solid dried under high vacuum for 3-4 hours. After thoroughly drying the isolated silver complex, a solution of 1.1eq of iodine in dry CH_2Cl_2 was added dropwise by syringe. Immediately, light yellow silver iodide precipitated out and the addition continued until no further precipitate appeared and a light pink color persisted. The reaction mixture was stirred for 15 min. Thereafter, the vial was centrifuged for 10 min at 2000 rpm. The resulting pink supernatant was transferred to another oven dried 20 mL vial, sealed with a rubber septum, and kept under an argon atmosphere. Addition of dry *n*-hexane (12.0-15.0 mL) resulted in the formation of a light yellowish precipitate. Addition of hexanes was continued until no additional precipitation could be observed. The vial was centrifuged for 10 min at 2000 rpm. The supernatant was removed, and the resulting yellowish solid was dried under vacuum generating an isotopologue mixture of [bis(pyridine)iodine]$^+$ complexes as a crystalline solid.

For the isotopic perturbation of equilibrium studies, the isotopologue mixture for complexes **10-I** and **12-I** were generated following the procedure reported for the [bis(pyridine)iodine]$^+$ (**1-I**/**1-I-*d*** - **8-I**/**8-I-*d***) complexes, as a mixture (1 mmol *versus* 0.3 mmol) of the deuterated and nondeuterated analogues. The preparation of a mixture of **11-I**/**11-I-*d*** was previously reported.[1,2]

NMR Spectroscopy. Isotopic perturbation of equilibrium NMR experiments were recorded on a Varian VNMR-S 500 MHz spectrometer equipped with a ^{1}H-^{19}F/^{15}N-^{31}P 5 mm PFG dual broadband probe using broadband ^{1}H and inverse-gated ^{2}H decoupling, and ^{13}C detection at 125 MHz. The spectra of the mixtures of the non-deuterated and mono-deuterated compounds were recorded for CD_2Cl_2 (δ_H 5.32, δ_C 54.00) solution. ^{1}H and ^{13}C NMR spectra of all complexes were recorded for the temperature interval 25 °°C to -40 °C. To determinate small variations of isotopic shifts ($^{n}\Delta_{obs}$), ^{13}C NMR spectra were recorded with 32768 data points and a reduced spectral window of 18656.7 Hz providing a 0.56 Hz/point acquired spectral resolution. The data was then zero-filled to 262144 points providing a digital resolution of 0.07 Hz/point, using the software MestreNova V10.0. Apodization was applied with the MNova functions gaussian=0.5 and exponential=0.5. Error estimates for the IPE measurements are comparable to that of previous related investigations.[1,2] Diffusion NMR measurements were performed on a Varian 500 MHz VNMR-S spectrometer using the ONE-SHOT pulse sequence as implemented in the software VNMRJ version 3.2. Sixteen scans were acquired using 60 s relaxation delay (d1), 2 ms diffusion-encoding / decoding gradient pulse duration and 25 ms diffusion delay. The z-gradient strengths were varied in 15 steps between 0 and 60 G/cm. Gradient strength was calibrated for the known diffusion coefficient of the residual water signal of a DMSO-d_6 sample. Nitrogen chemical shifts were acquired using ^{1}H-^{15}N-HMBCAD pulse sequence on a 400 MHz Varian-MR spectrometer equipped with a OneNMR probe, or on a 500 MHz Varian VNMR-S 500 MHz spectrometer equipped with a ^{1}H-^{19}F/^{15}N-^{31}P 5 mm PFG dual broadband probe.

Compound characterization.

[Bis(pyridine)silver] tetrafluoroborate (1-Ag). ^{1}H NMR (499.88 MHz, CD_2Cl_2) δ 8.69-8.71 (m, 4H, H2 and H6), 7.97 (tt, J = 7.7, 1.7 Hz, 2H, H4), 7.54-7.58 (m, 4H, H3 and H5); ^{13}C NMR (125.71 MHz, CD_2Cl_2) δ 152.6 (C2 and C6), 140.2 (C4), 126.3 (C3 and C5); ^{19}F NMR (470.31 MHz, CD_2Cl_2) δ -150.08 ($^{10}BF_4$), -150.13 ($^{11}BF_4$); ^{15}N NMR (50.67 MHz, CD_2Cl_2) δ -126.5. ^{15}N NMR (50.67 MHz, CD_2Cl_2) δ -126.5.

[Bis(pyridine)iodine] tetrafluoroborate (1-I/1-I-*d*). ^{1}H NMR (499.88 MHz, CD_2Cl_2) δ 8.79-8.82 (m, 4H, H2 and H6), 8.25 (m, 2H, H4), 7.64-7.68 (m 4H, H3 and H5); $^{13}C\{^1H,^2H\}$NMR (125.71 MHz, CD_2Cl_2) δ 150.0 (C2 and C6) 142.7 (C4), 128.5 (C3 and C5); 149.7 (C2-*d*), 149.9 (C6-*d*), 142.7 (C4-*d*), 128.3 (C3-*d*) ^{19}F NMR (470.31 MHz, CD_2Cl_2) δ -150.39 ($^{11}BF_4$), -150.45 ($^{10}BF_4$); ^{15}N NMR (50.67 MHz, CD_2Cl_2) δ -175.1. HR(ESI)MS calcd $C_{10}H_{10}IN_2^+$ for *m/z* 288.9989, found 288.9812.

[Bis(pyridine)silver] perchlorate (2-Ag). ^{1}H NMR (499.88 MHz, CD_2Cl_2) δ 8.71-8.75 (m, 4H, H2 and H6), 7.94 (tt, J = 7.7, 1.7 Hz, 2H, H4), 7.51-7.55 (m, 4H, H3 and H5); ^{13}C NMR (100.58 MHz, CD_2Cl_2) δ 152.6 (C2 and C6), 139.9 (C4), 126.1 (C3 and C5); ^{15}N NMR (50.67 MHz, CD_2Cl_2) δ -124.0.

[Bis(pyridine)iodine] perchlorate (2-I/2-I-*d*).[2] ^{1}H NMR (499.88 MHz, CD_2Cl_2) δ 8.77-8.81 (m, 4H, H2 and H6), 8.20-8.25 (m, 2H, H4), 7.61-7.66 (m, 4H, H3 and H5); $^{13}C\{^1H,^2H\}$NMR (125.71 MHz, CD_2Cl_2) δ 150.1 (C2 and C6), 142.7 (C4), 128.5 (C3 and C5), 149.7 (C2-*d*), 149.9 (C6-*d*), 142.7 (C4-*d*), 128.3 (C3-*d*); ^{15}N NMR (50.67 MHz, CD_2Cl_2) δ -175.0. HR(ESI)MS calcd $C_{10}H_{10}IN_2^+$ for *m/z* 288.9889, found 288.9731.

[Bis(pyridine)silver] hexafluorophosphate (3-Ag). ^{1}H NMR (499.88 MHz, CD_2Cl_2) δ 8.64-8.68 (m, 4H, H2 and H6), 8.02 (tt, J = 7.7 and 1.7 Hz, 2H, H4), 7.59-7.62 (m, 4H, H3 and H5); ^{13}C NMR (125.71 MHz, CD_2Cl_2) δ 152.5 (C2 and C6) 140.5 (C4), 126.5 (C3 and C5); ^{19}F NMR (470.31 MHz, CD_2Cl_2) δ -70.7, (d, 6F, $^1J_{P-F}$ = 713 Hz); ^{15}N NMR (50.67 MHz, CD_2Cl_2) δ -128.4.

[Bis(pyridine)iodine] hexafluorophosphate (3-I/3-I-*d*). ^{1}H NMR (499.88 MHz, CD_2Cl_2) δ 8.72-8.78 (m, 4H, H2 and H6), 8.22 (m, 2H, H4), 7.59-7.65 (m, 4H, H3 and H5); $^{13}C\{^1H,^2H\}$NMR (125.71 MHz, CD_2Cl_2) δ 149.9 (C2 and C6) 142.7 (C4), 128.4 (C3 and C5); 149.6 (C2-*d*), 149.9 (C6-*d*), 142.7 (C4-*d*), 128.3 (C3-*d*) ^{19}F NMR (470.31 MHz, CD_2Cl_2) δ (d, $^1J_{P-F}$ = 711 Hz); ^{15}N NMR (50.67 MHz, CD_2Cl_2) δ -175.1; HR(ESI)MS calcd $C_{10}H_{10}IN_2^+$ for *m/z* 288.9889, found 288.9794.

[Bis(pyridine)silver] hexafluoroantimonate (4-Ag). ^{1}H NMR (499.88 MHz, CD_2Cl_2) δ 8.62-8.66 (m, 4H, H2 and H6), 8.04 (tt, J = 7.7, 1.7 Hz, 2H, H4), 7.60-7.65 (m, 4H, H3 and H5); ^{13}C NMR (125.71 MHz, CD_2Cl_2) δ 152.2 (C2 and C6) 140.7 (C4), 126.6 (C3 and C5); ^{19}F NMR (470.31 MHz, CD_2Cl_2) -111 to -136 (br m); ^{15}N NMR (50.67 MHz, CD_2Cl_2) δ -129.3.

[Bis(pyridine)iodine] hexafluoroantimonate (4-I/4-I-*d*). ^{1}H NMR (499.88 MHz, CD_2Cl_2) δ 8.73-8.78 (m, 4H, H2 and H6), 8.22 (m, 2H, H4), 7.60-7.63 (m 4H, H3 and H5); $^{13}C\{^1H,^2H\}$NMR (125.71 MHz, CD_2Cl_2) δ 150.1 (C2 and C6) 142.7 (C4), 128.5 (C3 and C5); 149.6 (C2-*d*), 149.9 (C6-*d*), 142.7 (C4-*d*), 128.3 (C3-*d*); ^{19}F NMR (470.31 MHz, CD_2Cl_2); ^{15}N NMR (50.67 MHz, CD_2Cl_2) δ -175.8. HR(ESI)MS calcd $C_{10}H_{10}IN_2^+$ for *m/z* 288.9889, found 288.9881.

[Bis(pyridine)silver] triflate (5-Ag). ^{1}H NMR (499.88 MHz, CD_2Cl_2) δ 8.70-8.73 (m, 4H, H2 and H6), 7.90 (tt, J = 7.7, 1.7 Hz, 2H, H4), 7.47-7.50 (m, 4H, H3 and H5); ^{13}C NMR (125.71 MHz, CD_2Cl_2) δ 152.5 (C2 and C6) 139.6 (C4), 125.9 (C3 and C5), 121.3 (q, J = 320.3 Hz, CF_3); ^{19}F NMR (470.31 MHz, CD_2Cl_2) δ -76.4; ^{15}N NMR (50.67 MHz, CD_2Cl_2) δ -122.0.

[Bis(pyridine)iodine] triflate (5-I/5-I-*d*).[1] ^{1}H NMR (399.95 MHz, CD_2Cl_2) δ 8.78-8.81 (m, 4H, H2 and H6), 8.22 (m, 2H, H4), 7.61-7.65 (m 4H, H3 and H5); $^{13}C\{^1H,^2H\}$NMR (125.71 MHz, CD_2Cl_2) δ 150.0 (C2),149.7 (C2-*d*), 150.0 (C6), 150.2

(C6-*d*), 142.9 (C4-*d*), 142.7 (C–4), 120.9 (C3), 128.9 (C–5) 128.9 (C5-*d*), 120.4 (C3-*d*), 121.54 (q, *J* = 320.7 Hz, CF_3); ^{19}F NMR (376.29 MHz, CD_2Cl_2) δ -77.24 (s, CF_3); ^{15}N NMR (50.67 MHz, CD_2Cl_2) δ -175.1.

[Bis(pyridine)silver] tosylate (6-Ag). ^{1}H NMR (499.88 MHz, CD_2Cl_2) δ 8.70-8.72 (m, 4H, H2 and H6), 7.80 (tt, *J* = 7.7, 1.7 Hz, 2H, H4), 7.66-7.69 (m, 2H, Ts-H2 and Ts-H6), 7.35-7.39 (m, 4H, H3 and H5), 7.09-7.12 (m, 2H, Ts-H3 and Ts-H5), 2.33 (s, 3H, CH_3); ^{13}C NMR (100.58 MHz, CD_2Cl_2) δ 152.4 (C2 and C6) 143.8 (Ts-C1), 140.2 (Ts-C4), 138.6 (C4), 129.1 (Ts-C3 and Ts-C5), 126.4 (C3 and C5), 125.4 (Ts-C2 and Ts-C6), 21.5 (Ts-C4-$\underline{C}H_3$); ^{15}N NMR (50.67 MHz, CD_2Cl_2) δ -111.8.

[Bis(pyridine)iodine] tosylate (6-I/6-I-*d*). ^{1}H NMR (499.88 MHz, CD_2Cl_2) δ 8.79-8.83 (m, 4H, H2 and H6), 8.20 (m, 2H, H4), 7.69-7.73 (m, 2H, Ts-H2 and Ts-H6), 7.57-7.66 (m, 4H, H3 and H5), 7.08-7.17 (m, 2H, Ts-H3 and Ts-H5), 2.32 (s, 3H, CH_3); ^{13}C{^{1}H,^{2}H}NMR (125.71 MHz, CD_2Cl_2) δ 150.1 (C2 and C6), 145.7 (Ts-C1), 142.6 (C4), 139.2 (Ts-C4), 128.9 (Ts-C3 and Ts-C5), 128.5 (C3 and C5), 126.4 (Ts-C2 and Ts-C6), 21.5(Ts-C4-$\underline{C}H_3$); 149.6(C2-*d*), 149.9(C6-*d*), 142.5(C4-*d*), 128.2 (C3-*d*); ^{15}N NMR (50.67 MHz, CD_2Cl_2) δ -174.8, HR(ESI)MS calcd $C_{10}H_{10}IN_2^+$ for *m/z* 288.9889, found 288.9916.

[Bis(pyridine)silver] nitrate (7-Ag). ^{1}H NMR (499.88 MHz, CD_2Cl_2) δ 8.63-8.66 (m, 4H, H2 and H6), 7.86 (tt, *J* = 7.7, 1.7 Hz, 2H, H4), 7.44-7.47 (m, 4H, H3 and H5); ^{13}C NMR (100.58 MHz, CD_2Cl_2) δ 152.2 (C2 and C6) 139.0 (C4), 125.7 (C3 and C5); ^{15}N NMR (50.67 MHz, CD_2Cl_2) δ -113.1.

[Bis(pyridine)iodine] nitrate (7-I/7-I-*d*).[2] ^{1}H NMR (499.88 MHz, CD_2Cl_2) δ 8.82-8.85 (m, 4H, H2 and H6), 8.19-8.25 (m, 2H, H4), 7.61-7.68 (m, 4H, H3 and H5); ^{13}C{^{1}H,^{2}H}NMR (100.58 MHz, CD_2Cl_2) δ 150.1 (C2 and C6), 142.6 (C4), 128.4 (C3 and C5); 140.3 (C2-*d),* 150.4 (C6-*d*), 140.0 (C4-*d*), 120.0 (C3-*d*), 120.5 (C5-*d*) ^{15}N NMR (50.67 MHz, CD_2Cl_2) δ -174.8. HRMS calcd $C_{10}H_{10}IN_2^+$ for *m/z* 284.9889, found 284.9923. HR(ESI)MS calcd $C_{10}H_{10}IN_2^+$ for *m/z* 288.9889, found 288.9923.

[Bis(pyridine)silver] trifluoroacetate (8-Ag). ^{1}H NMR (499.88 MHz, CD_2Cl_2) δ 8.59-8.61 (m, 4H, H2 and H6), 7.85 (tt, *J* = 7.7, 1.7 Hz, 2H, H4), 7.43-7.45 (m, 4H, H3 and H5). ^{13}C NMR (125.71 MHz, CD_2Cl_2) δ 152.0 (C2 and C6), 138.7 (C4), 125.5 (C3 and C5) ^{15}N NMR (50.67 MHz, CD_2Cl_2) δ -108.5.

[Bis(pyridine)iodine] trifluoroacetate (8-I/8-I-*d*). ^{1}H NMR (499.88 MHz, CD_2Cl_2) δ 8.74-8.75 (m, 4H, H2 and H6), 8.11-8.14 (m, 2H, H4), 7.49-7.52 (m, 4H, H3 and H5); ^{13}C{^{1}H,^{2}H}NMR (125.71 MHz, CD_2Cl_2) δ 150.7 (C2 and C6), 141.8 (C4), 128.0 (C3 and C5) ;150.4 (C2-*d*), 150.7 (C6-*d*), 141.9 (C4-*d*), 127.9 (C3-*d*), 162.0 (q, $J_{\underline{C}F3}$ = 37.2 Hz) 112.8 (q, $^{1}J_{\underline{C}F3}$ = 290.4 Hz) ^{15}N NMR (50.67 MHz, CD_2Cl_2) δ -175.2. HR(ESI)MS calcd $C_{10}H_{10}IN_2^+$ for *m/z* 284.9889, found 284.9857.

[Bis(pyridine)iodine] tetrafluoroborate in the presence of [(Bn_{12}BU[6])] (9-I/9-I-*d*). ^{1}H NMR (499.88 MHz, CD_2Cl_2) δ 8.59-8.60 (m, 4H, H2 and H6), 8.08-8.11 (m, 2H, H4), 7.46-7.49 (m 4H, H3 and H5); ^{13}C{^{1}H,^{2}H}NMR (125.71 MHz, CD_2Cl_2) δ 149.7 (C2 and C6) 142.6 (C4), 128.4 (C3 and C5); 149.3 (C2-*d*), 149.7 (C6-*d*), 142.7 (C4-*d*), 128.3 (C3-*d*); ^{19}F NMR (376.29 MHz, CD_2Cl_2) δ -155.9 ($^{10}BF_4$), 158.1 ($^{11}BF_4$); ^{15}N NMR (50.67 MHz, CD_2Cl_2) δ -175.5.

[(1,2-Bis(pyridin-2-ylethynyl)benzene)silver] tetrafluoroborate (10-Ag). ^{1}H NMR (399.95 MHz, CD_2Cl_2) δ 8.92 (ddd, *J* = 5.4, 1.7, 0.9 Hz, 2H, H2 and H2'), 7.97 (td, *J* = 7.8, 1.7 Hz, 2H, H4 and H4'), 7.80 (ddd, *J* = 7.9, 1.4, 0.9 Hz, 2H, H5 and H5'), 7.70 (dd, *J* = 5.8, 3.3 Hz, 2H, H10 and H10'), 7.58 (ddd, *J* = 7.7, 5.4, 1.4 Hz, 2H, H3 and H3'), 7.48 (dd, *J* = 5.8, 3.4 Hz, 2H, H11 and H11'); ^{13}C NMR (125.71 MHz, CD_2Cl_2) δ 153.6 (C2 and C2′) 143.8 (C6 and C6′), 140.0 (C4 and C4′), 133.6 (C10 and C10′), 130.7 (C11 and C11′), 128.9 (C5 and C5′), 125.8 (C3 and C3′), 124.0 (C9 and C9′), 92.5 (C7 and C7′), 91.2 (C8 and C8′).

[(1,2-Bis(pyridin-2-ylethynyl)benzene)iodine] tetrafluoroborate (10-I/10-I-*d*). ^{1}H NMR (399.95 MHz, CD_2Cl_2) δ 8.86 (ddd, *J* = 5.7, 1.6, 0.8 Hz, 2H, H2 and H2-*d*), 8.18 (td, *J* = 7.8, 1.5 Hz, 2H, H4 and H4-*d*), 7.91 (ddd, *J* = 7.9, 1.4, 0.7 Hz, 2H, H5 and H5-*d*), 7.79 (dd, *J* = 5.8, 3.3 Hz, 2H, H10 and H10-*d*), 7.60 (dd, *J* = 5.8, 3.3 Hz, 2H, H11 and H11-*d*), 7.50 (ddd, *J* = 7.6, 5.6, 1.4 Hz, 2H, H3 and H3-*d*); ^{13}C{^{1}H,^{2}H}NMR (125.71 MHz, CD_2Cl_2) δ 151.3 (C2), 150.9 (C2-*d*), 127.1 (C3), 126.9 (C3-*d*), 142.7 (C4), 142.74 (C4-*d*), 130.9 (C5 and C5-*d*), 143.2 (C6), 143.1 (C6'), 134.9 (C10 and C10-*d*), 131.5 (C11 and C11-*d*), 124.7 (C9 and C9-*d*), 99.1 (C8 and C8-*d*), 91.1 (C7 and C7-*d*); ^{15}N NMR (40.54 MHz, CD_2Cl_2) δ -165.5.

[(1,2-Bis(pyridin-2-ylethynyl)benzene)iodine] triflate (11-I/11-I-*d*).[1] ^{1}H NMR (499.88 MHz, CD_2Cl_2) δ 8.87 (ddd, 2H, *J* = 5.7, 1.5, 0.8 Hz, H2 and H2-*d*), 8.18 (ddd, 2H, *J* = 7.6, 7.9, 1.5 Hz, H4 and H4-*d*), 7.91 (ddd, 2H, *J* = 7.9, 1.4, 0.8 Hz, H5 and H5-*d*), 7.76-7.80 (AA' part of AA'BB', 2H, H10 and H10-*d*), 7.56-7.61 (BB' part of AA'BB', 2H, H11 and H11-*d*), 7.50 (ddd, 2H, *J* = 7.6, 5.7, 1.4 Hz, H3 and H3-*d*); ^{13}C{^{1}H,^{2}H}NMR (125.71 MHz, CD_2Cl_2) δ 151.2 (C2), 150.9 (C2-*d*), 143.0 (C6) 143.0 (C6-*d*), 142.7 (C4 and C4-*d*), 134.8 (C10 and C10-*d*), 131.4 (C11 and C11-*d*), 130.9 (C5 and C5-*d*), 127.0 (C3) 126.8 (C3-*d*), 124.6 (C9 and C9-*d*), 121.5 (q, *J* = 320.9 Hz, $\underline{C}F_3$), 99.0 (C8 and C8-*d*), 91.0 (C7 and C7-*d*); ^{19}F NMR (376.29 MHz, CD_2Cl_2) δ -76.76 (s, CF_3). ^{15}N NMR (40.54 MHz, CD_2Cl_2) δ -165.0.

[(1,2-Bis(pyridin-2-ylethynyl)benzene)silver] nitrate (12-Ag/12-Ag-*d*). ^{1}H NMR (399.95 MHz, CD_2Cl_2) δ 8.92 (ddd, *J* = 5.3, 1.7, 0.9 Hz, 2H, H2 and H2-*d*), 7.91 (td, *J* = 7.8, 1.7 Hz, 2H, H4 and H4-*d*), 7.77 (ddd, *J* = 7.9, 1.4, 0.9 Hz, 2H, H5 and H5-*d*), 7.71 (dd, *J* = 5.8, 3.3 Hz, 2H, H10 and H10-*d*), 7.51 (ddd, *J* = 7.7, 5.3, 1.4 Hz, 2H, H3 and H3-*d*), 7.47 (dd, *J* = 5.8, 3.3 Hz, 2H, H11 and H11-*d)*; ^{13}C{^{1}H,^{2}H}NMR (100.58 MHz, CD_2Cl_2) δ 153.3 (C2), 143.9 (C6), 139.3 (C4), 133.4 (C10), 130.4 (C11), 128.8 (C5), 125.42 (C3), 124.4 (C9), 92.9 (C8), 90.5 (C7). ^{15}N NMR (40.54 MHz, CD_2Cl_2) δ -108.0.

[(1,2-Bis(pyridin-2-ylethynyl)benzene)iodine] nitrate (12-I/12-I-*d*). ^{1}H NMR (499.88 MHz, CD_2Cl_2) δ 8.93 (d, *J* = 5.5 Hz, 2H, H2 and H2-*d*), 8.16 (td, *J* = 7.8, 1.4 Hz, 2H, H4 and H4-*d*), 7.90 (dd, *J* = 7.9, 1.4 Hz, 2H, H5 and H5-*d*), 7.77 (dd, *J* = 5.8, 3.3 Hz, 2H, H10 and 10-*d*), 7.56 (dd, *J* = 5.8, 3.3 Hz, 2H, H11 and H11-*d*), 7.52-7.53 (m, 2H, H3 and H3-*d*); ^{13}C{^{1}H,^{2}H}NMR (125.71 MHz, CD_2Cl_2) δ 151.4 (C2), 150.4 (C2-*d*), 127.04 (C3), 126.89 (C3-*d*), 142.61 (C4), 142.63 (C4-*d*), 130.8 (C5 and C5-*d*),143.0 (C6), n.d (C6-*d*), 134.8 (C10 and C10-*d*), 131.4 (C11 and C11-*d*), 124.69 (C9 and C9-*d*), 98.99 (C8 and C8-*d*), 91.14 (C7 and C7-*d*); ^{15}N NMR (40.54 MHz, CD_2Cl_2) δ -163.0.

The ^{1}H and ^{13}C NMR spectra for each compound are shown as Figures S15-S52 on page S76-S96.

2. NMR SPECTRA REFERRED TO IN THE MAIN TEXT

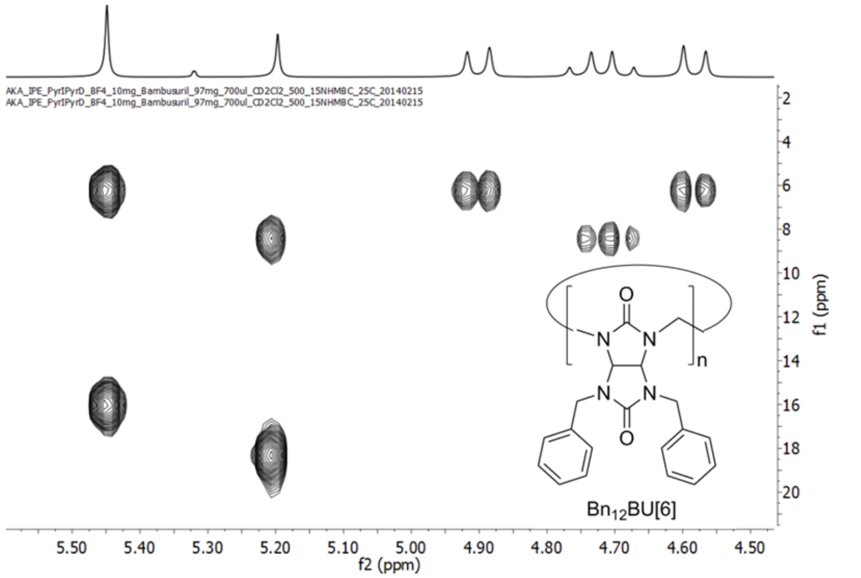

Figure S1. $^{1}H,^{15}N$ gHMBC spectrum of **9-I** acquired at 25°C at (499.89, 50.67 MHz) in CD_2Cl_2. Separate sets of NMR signals are seen for the free and the BF_4^--bound $Bn_{12}BU[6]$.[3-5] δ (^{15}N, $CH_3\underline{N}$) = 18.4 ppm (free), δ (^{15}N, $CH_3\underline{N}$) = 16.0 ppm (BF_4^- -bound); δ (^{15}N, $CH_2\underline{N}$) = 8.4 ppm (free), δ (^{15}N, $CH_2\underline{N}$) = 6.3 ppm (BF_4^- bound).

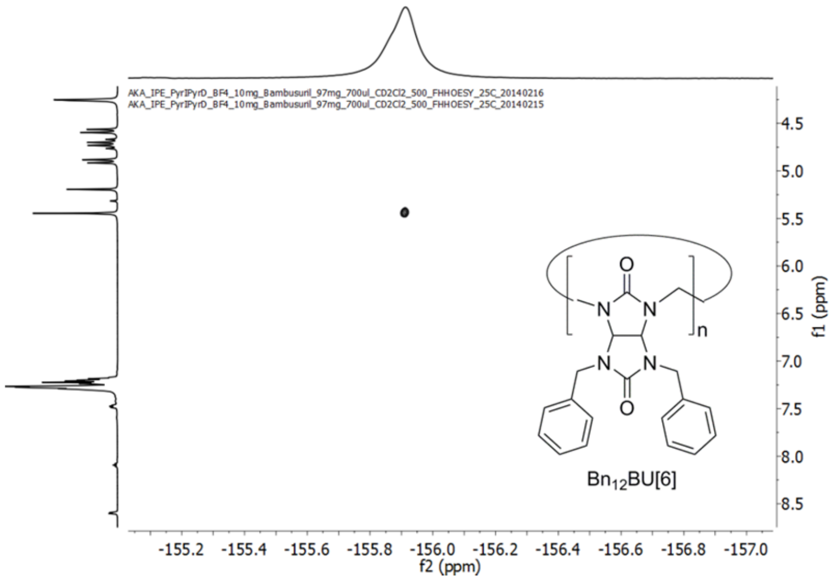

Figure S2. $^{1}H,^{19}F$ HOESY spectrum of **9-I** acquired at 25°C at (499.89, 50.67 MHz) in CD_2Cl_2 showing an intermolecular heteronuclear Overhauser effect between BF_4^- and the CH-group of $Bn_{12}BU[6]$.[3-5]

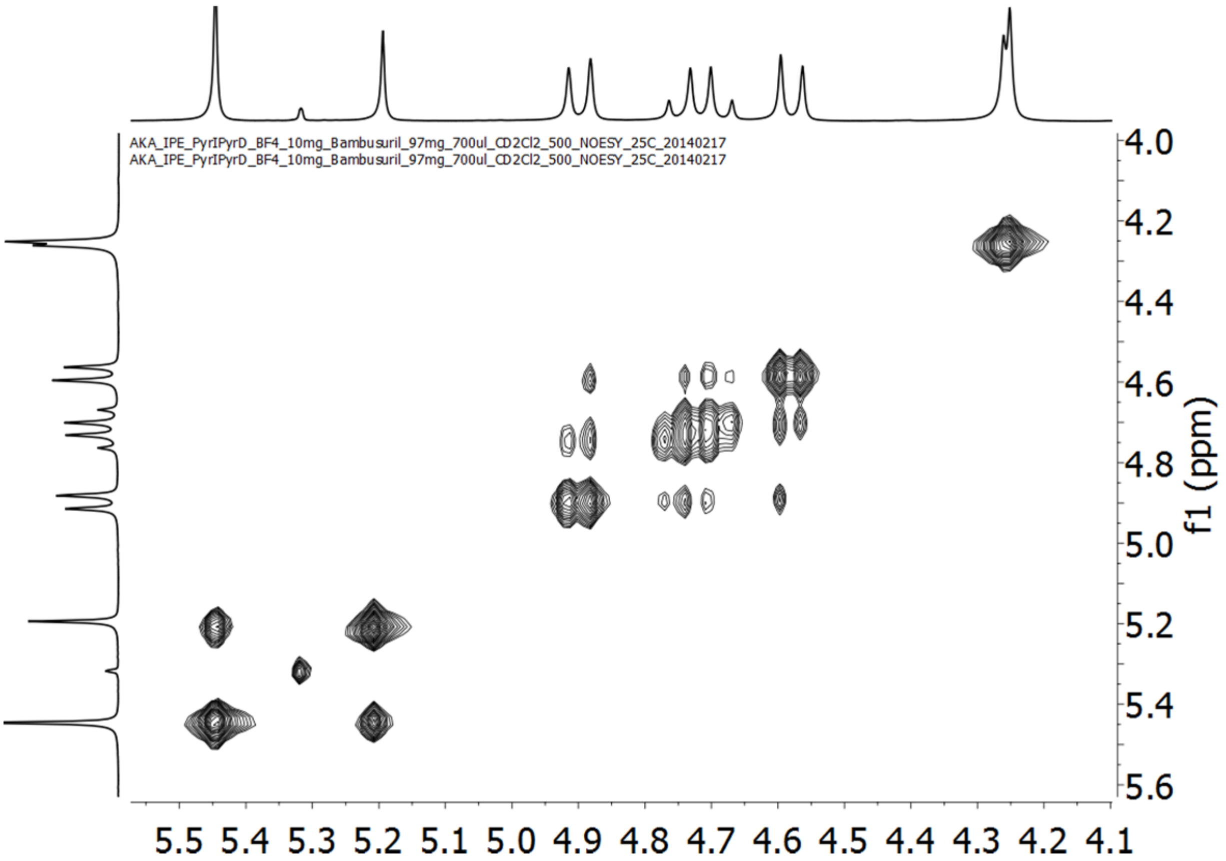

Figure S3. The EXSY spectrum of **9-I** acquired at 25°C at (499.89, 50.67MHz) in CD_2Cl_2 revealing that the BF_4^- is rapidly moving between the $Bn_{12}BU[6]$ units. Exchange crosspeaks are seen between the corresponding signals (CH_2 to CH_2) of the free and the BF_4^- bound forms of $Bn_{12}BU[6]$.

3. NMR CHEMICAL SHIFTS AND THEIR TEMPERATURE DEPENDENCE

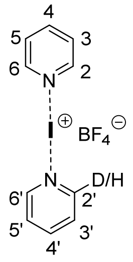

Table S1. The chemical shifts of [bis(pyridine)iodine] (1) tetrafluoroborate (1-I/1-I-*d*), given in ppm.

T (°C)	C-2/C-6 (H)	C-4 (H)	C-3/C-5 (H)	C-2 (D)	C-6 (D)	C-4 (D)	C-3 (D)	C-5 (D)
20	149.9939	142.647	128.4659	149.6611	149.9624	142.6668	128.321	128.4659
10	149.8907	142.5941	128.4031	149.5611	149.8594	142.6137	128.2569	128.4031
0	149.786	142.5382	128.3365	149.4514	149.7545	142.558	128.1888	128.3365
-10	149.6787	142.4788	128.2666	149.3432	149.6463	142.4987	128.1178	128.2666
-20	149.5726	142.4191	128.1965	149.2364	149.5399	142.4395	128.0464	128.1965
-30	149.4549	142.3547	128.1199	149.1223	149.4262	142.3746	127.9686	128.1199
-40	149.3569	142.295	128.0494	149.0182	149.323	142.3149	127.897	128.0494

Table S2. The temperature dependence of the isotope shifts () observed for 1-I/1-I-*d*, given in ppm.

T(K)	1/T (K^{-1})	$^{1}\Delta_{obs}$ $\delta_{(C2D-C2)}$	$^{2}\Delta_{obs}$ $\delta_{(C3D-C3)}$	$^{3}\Delta_{obs}$ $\delta_{(C4D-C4)}$	$^{4}\Delta_{obs}$ $\delta_{(C5D-C5)}$	$^{3}\Delta_{obs}$ $\delta_{(C6D-C6)}$
293	0.0034	-0.3328	-0.1449	0.0198	0	-0.0315
283	0.0035	-0.3296	-0.1462	0.0196	0	-0.0313
273	0.0037	-0.3346	-0.1477	0.0198	0	-0.0315
263	0.0038	-0.3355	-0.1488	0.0199	0	-0.0324
253	0.0040	-0.3362	-0.1501	0.0204	0	-0.0327
243	0.0041	-0.3326	-0.1513	0.0199	0	-0.0287
233	0.0043	-0.3387	-0.1524	0.0199	0	-0.0339

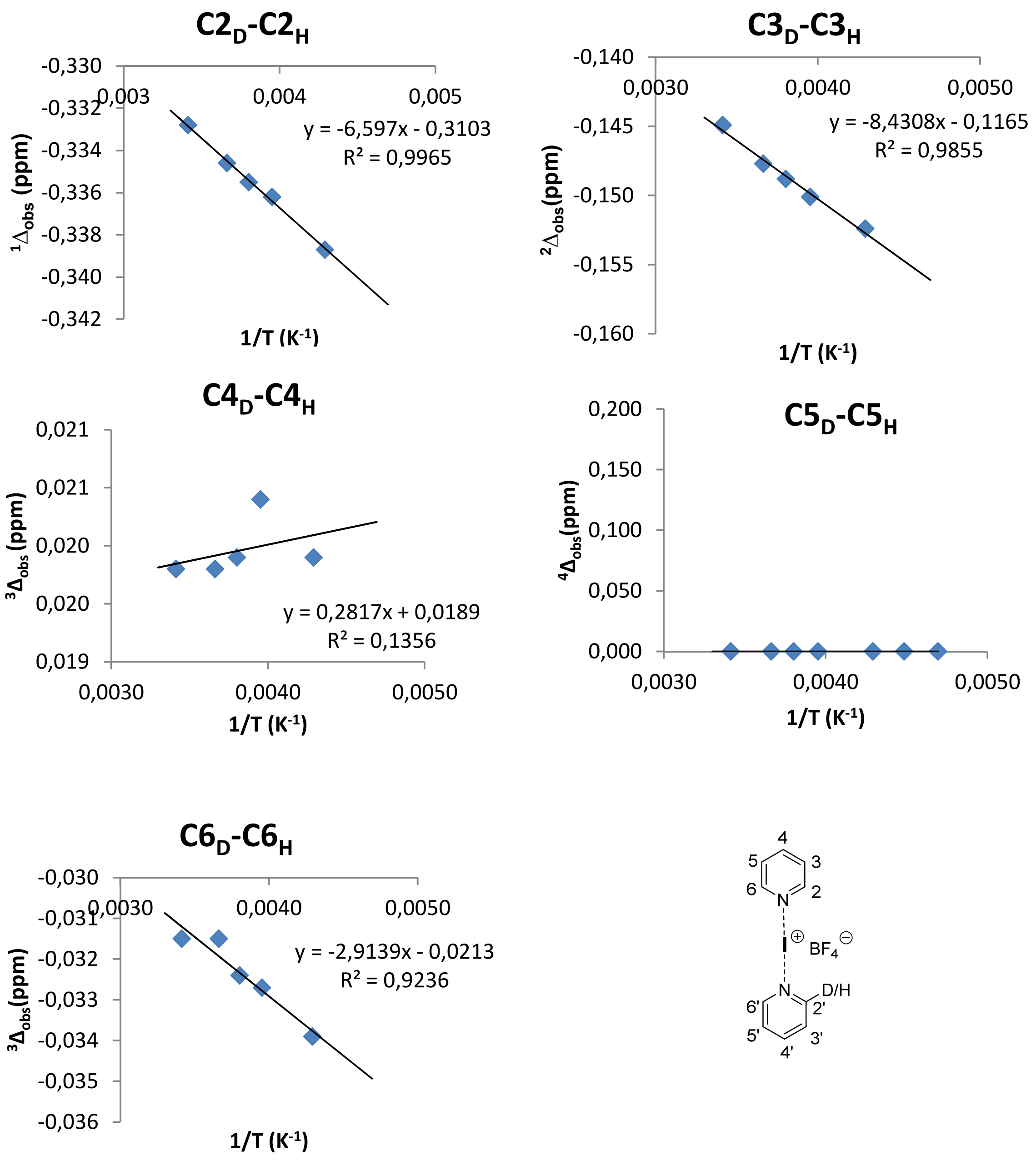

Figure S4. The temperature dependence of the isotope shifts of **1-I**/**1-I-*d*** are shown, for each carbon separately.

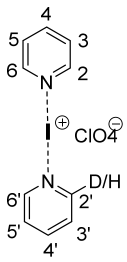

Table S3. The chemical shifts of [bis(pyridine)iodine] perchlorate (2-I/2-I-*d*), given in ppm.

T (°C)	C-2/C-6 (H)	C-4 (H)	C-3/C-5 (H)	C-2 (D)	C-6 (D)	C-4 (D)	C-3 (D)	C-5 (D)
25	150.0267	142.6553	128.4684	149.6916	149.9971	142.6749	128.3231	128.4684
10	149.876	142.5753	128.3751	149.5398	149.8458	142.5952	128.2278	128.3751
0	149.7682	142.517	128.3068	149.4302	149.7368	142.5377	128.1583	128.3068
-10	149.6672	142.4615	128.2416	149.3288	149.6356	142.4816	128.0916	128.2416
-20	149.5566	142.3987	128.1685	149.2176	149.5246	142.419	128.0172	128.1685
-30	149.4475	142.3362	128.1199	149.1078	149.415	142.3564	127.9686	128.1199
-40	149.3349	142.2699	128.0179	148.9944	149.3019	142.29	127.8643	128.0179

Table S4. The temperature dependence of the isotope shifts observed for (2-I/2-I-*d*), given in ppm.

T (K)	1/T (K^{-1})	$^{1}\Delta_{obs}$ δ_{C2D-C2}	$^{2}\Delta_{obs}$ δ_{C3D-C3}	$^{3}\Delta_{obs}$ δ_{C6D-C6}	$^{3}\Delta_{obs}$ δ_{C4D-C4}	$^{4}\Delta_{obs}$ δ_{C5D-C5}
298	0.033	-0.3351	-0.0296	0.0196	-0.1453	0
283	0.035	-0.3362	-0.0302	0.0199	-0.1473	0
273	0.036	-0.338	-0.0314	0.0207	-0.1485	0
263	0.038	-0.3384	-0.0316	0.0201	-0.15	0
253	0.039	-0.339	-0.032	0.0203	-0.1513	0
243	0.041	-0.3397	-0.0325	0.0202	-0.1513	0
233	0.042	-0.3405	-0.033	0.0201	-0.1536	0

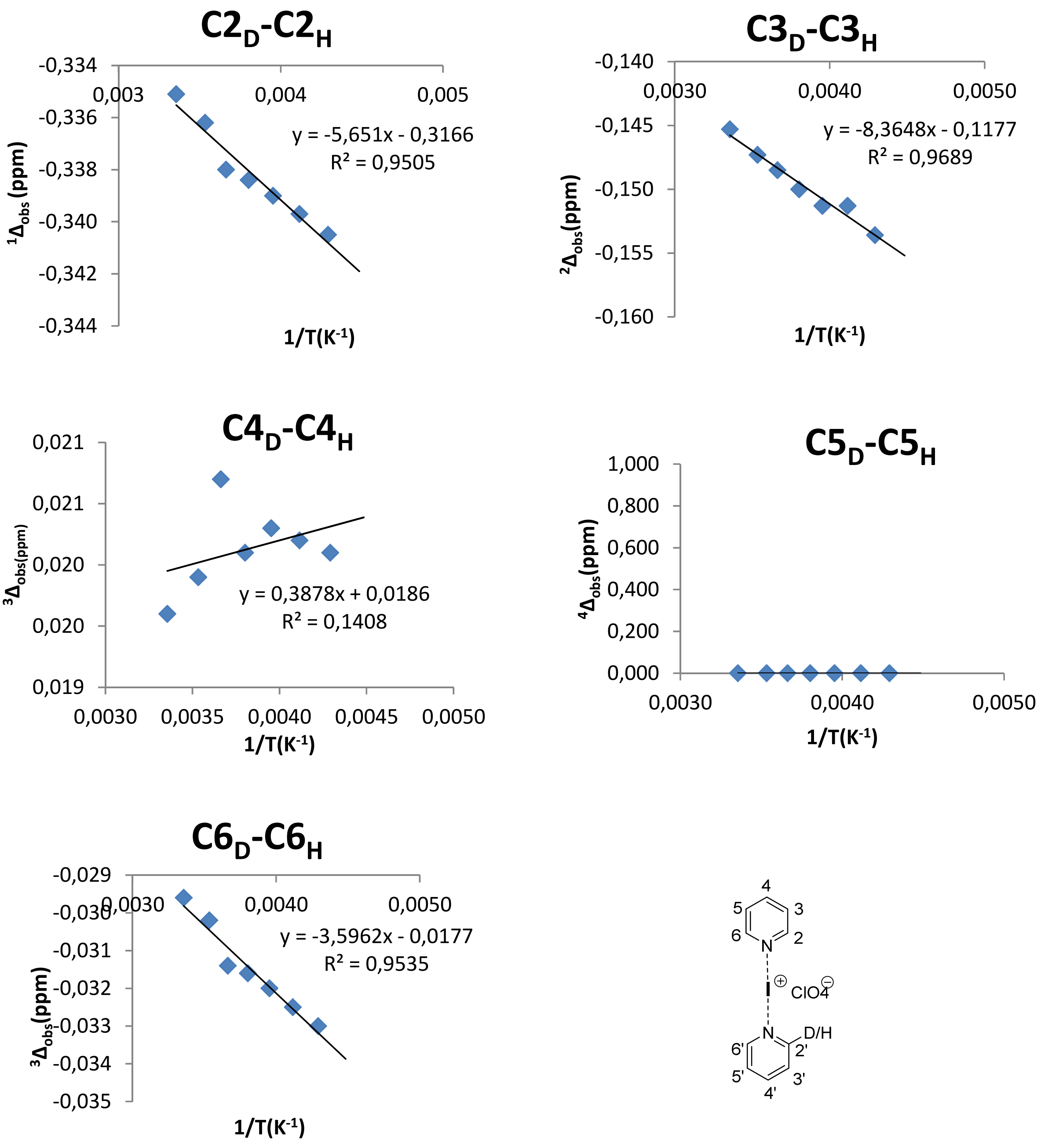

Figure S5. The temperature dependence of the isotope shifts of **2-I**/**2-I-*d*** shown for each carbon separately.

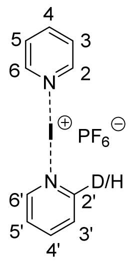

Table S6. The chemical shifts of [bis(pyridine)iodine] hexafluorophosphate (3-I/3-I-*d*), given in ppm.

T (°C)	C-2/C-6 (H)	C-4 (H)	C-3/C-5 (H)	C-2 (D)	C-6 (D)	C-4 (D)	C-3 (D)	C-5 (D)
25	149.9338	142.6812	128.4827	149.6291	149.9344	142.7009	128.3372	128.4827
10	149.8218	142.6041	128.3923	149.4873	149.7913	142.6235	128.2451	128.3923
0	149.7446	142.5614	128.3423	149.4090	149.7138	142.5809	128.1939	128.3423
-10	149.6131	142.4858	128.2542	149.2764	149.5814	142.5054	128.1040	128.2542
-20	149.5071	142.4246	128.1826	149.1693	149.4751	142.4444	128.0312	128.1826
-30	149.4012	142.3617	128.1088	149.0627	149.3686	142.3814	127.9562	128.1088
-40	149.2931	142.2969	128.0329	148.9538	149.2601	142.3166	127.8790	128.0329

Table S7. The temperature dependence of the isotope shifts observed for (3-I/3-I-*d*) given in ppm.

		${}^{1}\Delta_{obs}$	${}^{2}\Delta_{obs}$	${}^{3}\Delta_{obs}$	${}^{3}\Delta_{obs}$	${}^{4}\Delta_{obs}$
T (K)	1/T (K^{-1})	δ_{C2D}-δ_{C2}	δ_{C3D}-δ_{C3}	δ_{C4D}-δ_{C4}	δ_{C6D}-δ_{C6}	δ_{C5D}-δ_{C5}
298	0.033	-0.3047	0.0006	0.0197	-0.1455	0.0000
283	0.035	-0.3345	-0.0305	0.0194	-0.1472	0.0000
273	0.036	-0.3356	-0.0308	0.0195	-0.1484	0.0000
263	0.038	-0.3367	-0.0317	0.0196	-0.1502	0.0000
253	0.039	-0.3378	-0.0320	0.0198	-0.1514	0.0000
243	0.041	-0.3385	-0.0326	0.0197	-0.1526	0.0000
233	0.042	-0.3393	-0.0330	0.0197	-0.1539	0.0000

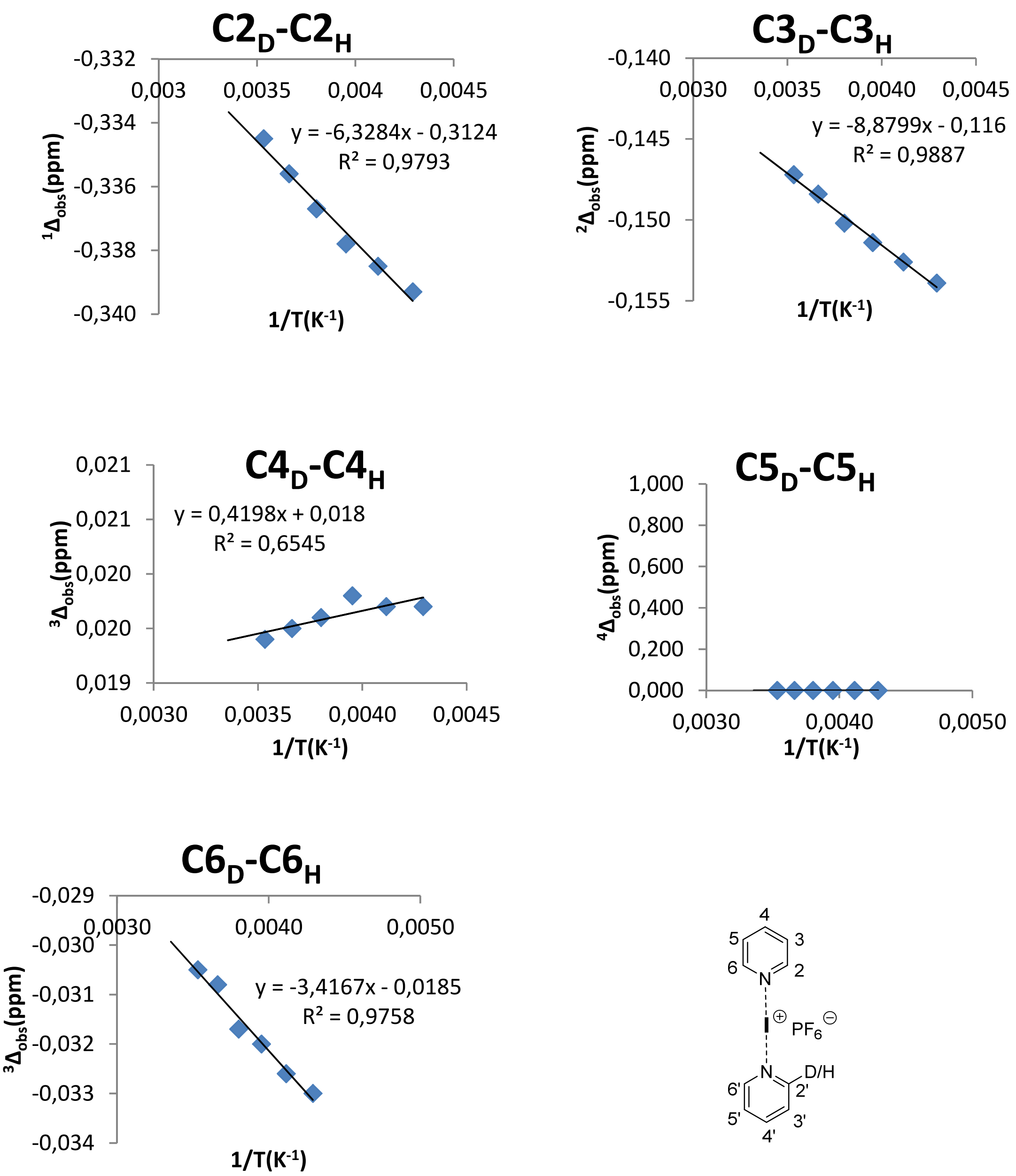

Figure S6. The temperature dependence of the isotope shifts of **3-I**/**3-I-*d*** shown for each carbon separately.

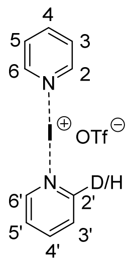

Table S8. The chemical shifts of [bis(pyridine)iodine] triflate (5-I/5-I-*d*), given in ppm.

T (°C)	C-2/C-6 (H)	C-4 (H)	C-3/C-5 (H)	C-2 (D)	C-6 (D)	C-4 (D)	C-3 (D)	C-5 (D)
25	150.0525	142.6765	128.4929	149.7147	150.0230	142.6958	128.3480	128.4929
10	149.9116	142.6042	128.4070	149.5751	149.8816	142.6240	128.2602	128.4070
0	149.7929	142.5403	128.3316	149.4551	149.7618	142.5600	128.1831	128.3316
-10	149.6848	142.4814	128.2621	149.3469	149.6535	142.5017	128.1127	128.2621
-20	149.5762	142.4206	128.1902	149.2370	149.5441	142.4411	128.0392	128.1902
-30	149.4623	142.3540	128.1130	149.1225	149.4298	142.3741	127.9609	128.1130
-40	149.3502	142.2888	128.0356	149.0089	149.3171	142.3086	127.8822	128.0356

Table S9. The temperature dependence of the isotope shifts observed for 5-I/5-I-*d*, given in ppm.

T (K)	1/T(K^{-1})	$^1\Delta_{obs}$ δ_{C2D}-δ_{C2}	$^2\Delta_{obs}$ δ_{C3D}-δ_{C3}	$^3\Delta_{obs}$ δ_{C4D}-δ_{C4}	$^3\Delta_{obs}$ δ_{C6D}-δ_{C6}	$^4\Delta_{obs}$ δ_{C5D}-δ_{C5}
298	0.0034	-0.3378	-0.0295	0.0193	-0.1449	0.0000
283	0.0035	-0.3365	-0.0300	0.0198	-0.1468	0.0000
273	0.0037	-0.3378	-0.0311	0.0197	-0.1485	0.0000
263	0.0038	-0.3379	-0.0313	0.0203	-0.1494	0.0000
253	0.0040	-0.3392	-0.0321	0.0205	-0.1510	0.0000
243	0.0041	-0.3398	-0.0325	0.0201	-0.1521	0.0000
233	0.0043	-0.3413	-0.0331	0.0198	-0.1534	0.0000

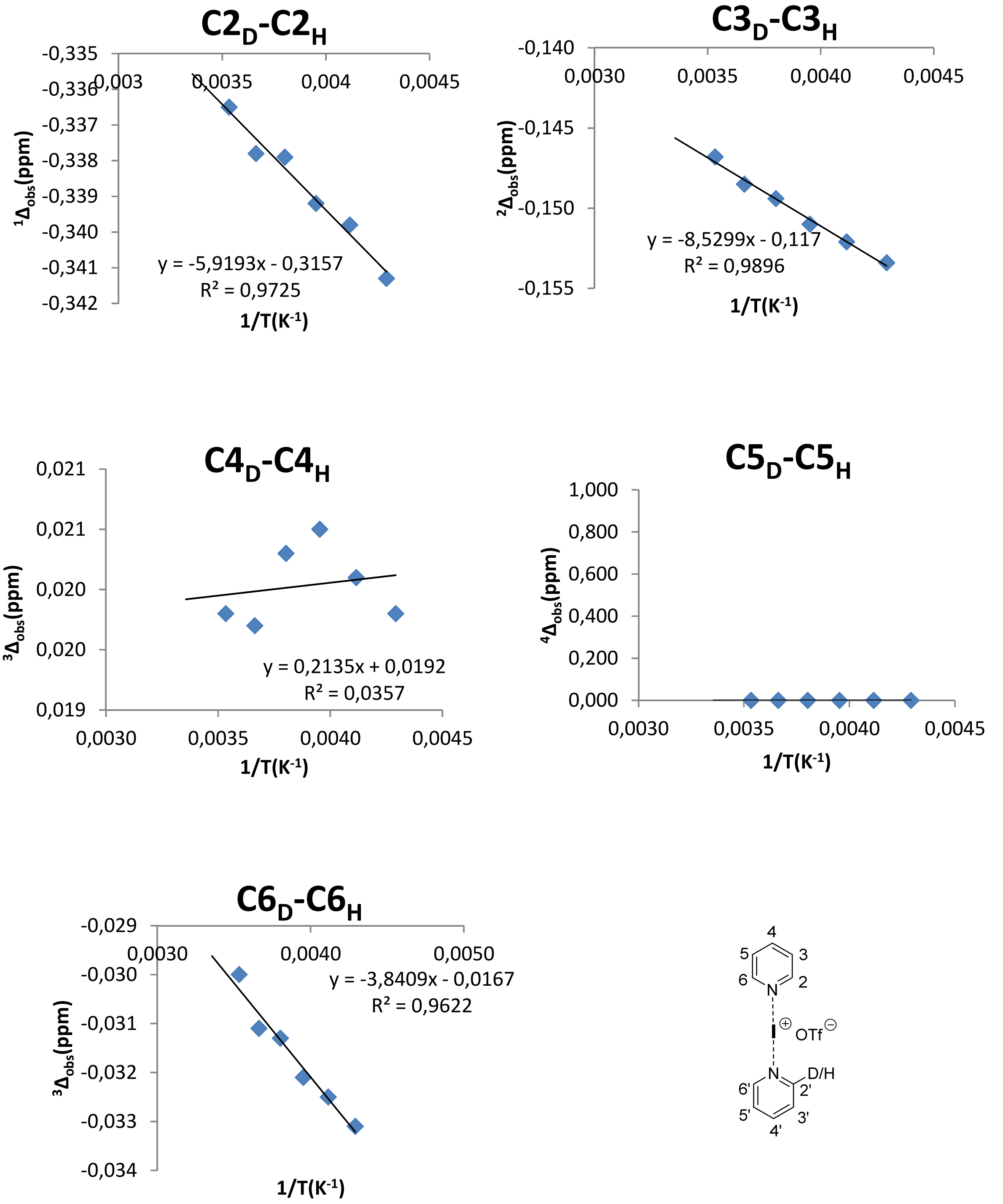

Figure S7. The temperature dependence of the isotope shifts of **5-I/5-I-*d*** shown for each carbon separately. Data for the temperature dependence of the isotope shifts of **5-H/5-H-*d*** is given in the supporting information of reference 2.

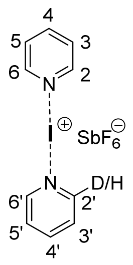

Table S10. The chemical shifts of [bis(pyridine)iodine] hexafluoroantimonate (4-I/4-I-*d*), given in ppm.

T (°C)	C-2/C-6 (H)	C-4 (H)	C-3/C-5 (H)	C-2 (D)	C-6 (D)	C-4 (D)	C-3 (D)	C-5 (D)
25	149.9619	142.6540	128.4480	149.6268	149.9324	142.6743	128.3026	128.4480
10	149.8237	142.5778	128.3597	149.4874	149.7935	142.5986	128.2122	128.3597
0	149.7163	142.5172	128.2892	149.3792	149.6853	142.5381	128.1400	128.2892
-10	149.6826	142.5279	128.2901	149.3443	149.6512	142.5488	128.1398	128.2901
-20	149.5072	142.3958	128.1479	149.1681	149.4754	142.4168	127.9965	128.1479
-30	149.3984	142.3304	128.0724	149.0585	149.3661	142.3515	127.9195	128.0724
-40	149.2884	142.2640	127.9949	148.9475	149.2557	142.2852	127.8410	127.9949

Table S11. The temperature dependence of the isotope shifts observed for 4-I/4-I-*d*, given in ppm.

T (K)	1/T(K^{-1})	$^{1}\Delta_{obs}$ δ_{C2D}-δ_{C2}	$^{2}\Delta_{obs}$ δ_{C3D}-δ_{C3}	$^{3}\Delta_{obs}$ δ_{C4D}-δ_{C4}	$^{3}\Delta_{obs}$ δ_{C6D}-δ_{C6}	$^{4}\Delta_{obs}$ δ_{C5D}-δ_{C5}
298	0.0034	-0.3351	-0.0295	0.0203	-0.1454	0.0000
283	0.0035	-0.3363	-0.0302	0.0208	-0.1475	0.0000
273	0.0037	-0.3371	-0.0310	0.0209	-0.1492	0.0000
263	0.0038	-0.3383	-0.0314	0.0209	-0.1503	0.0000
253	0.0040	-0.3391	-0.0318	0.0210	-0.1514	0.0000
243	0.0041	-0.3399	-0.0323	0.0211	-0.1529	0.0000
233	0.0043	-0.3409	-0.0327	0.0212	-0.1539	0.0000

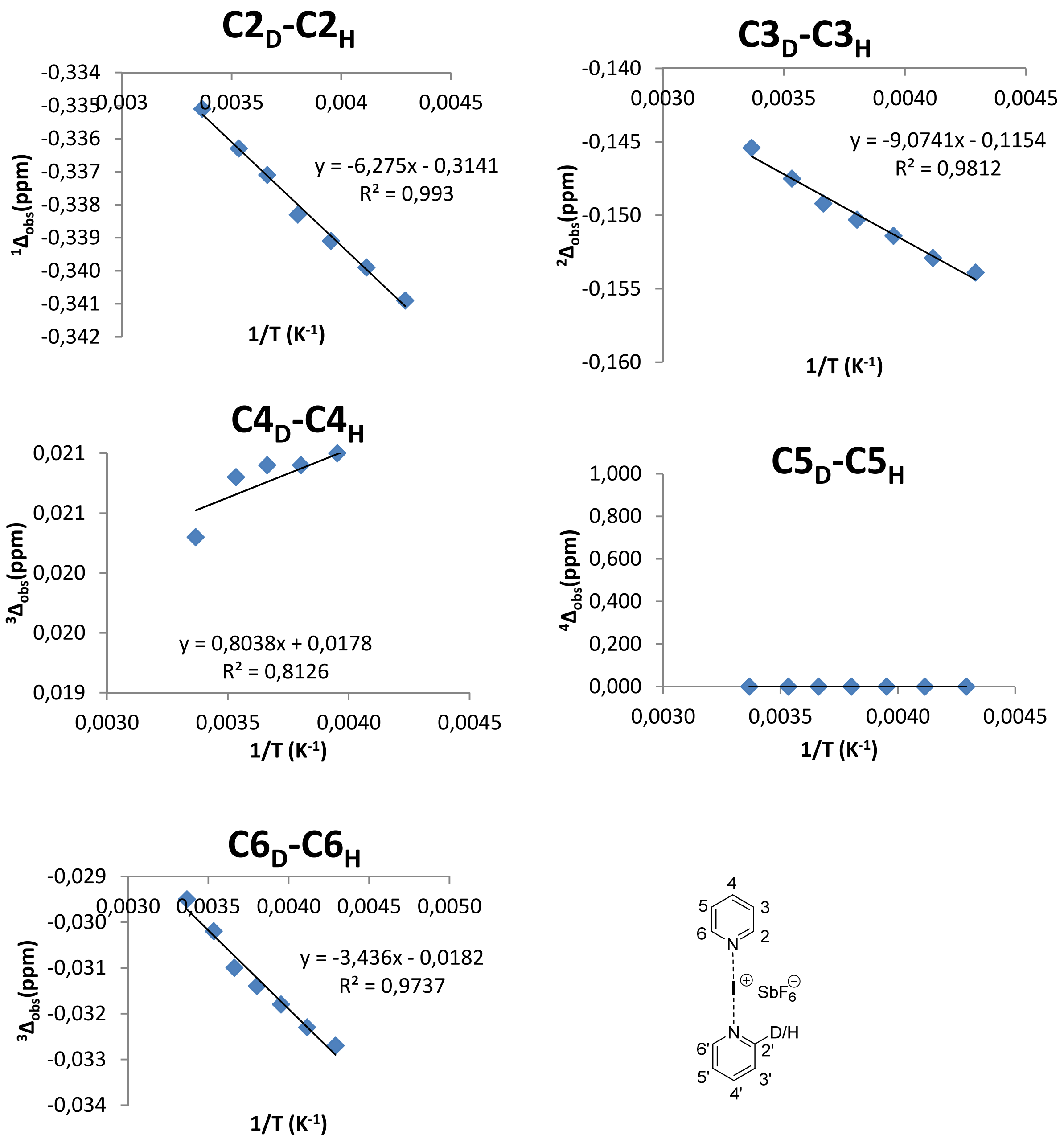

Figure S8. The temperature dependence of the isotope shifts of **4-I/4-I-*d***, shown for each carbon separately.

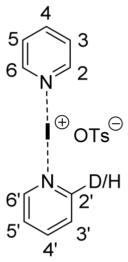

Table S12. The chemical shifts of [bis(pyridine)iodine] tosylate (6-I/6-I-*d*), given in ppm.

T (°C)	1/T	C-2/C-6 (H)	C-4 (H)	C-3/C-5 (H)	C-2 (D)	C-6 (D)	C-4 (D)	C-3 (D)	C-5 (D)
24	0.0034	149.9218	142.4566	128.3356	149.5856	149.8922	142.4745	128.1897	128.3356
10	0.0035	149.7955	142.3835	128.2541	149.4574	149.7644	142.4038	128.1065	128.2541
0	0.0037	149.6832	142.3197	128.1805	149.3448	149.6514	142.3401	128.0315	128.1805
-10	0.0038	149.5800	142.2601	128.1120	149.2415	149.5477	142.2806	127.9616	128.1120
-20	0.0040	149.4761	142.1988	128.0414	149.1370	149.4436	142.2192	127.8900	128.0414
-30	0.0041	149.3691	142.1355	127.9677	149.0296	149.3358	142.1556	127.8151	127.9677
-40	0.0043	149.2618	142.0701	127.9949	148.9217	149.2281	142.0903	127.8410	127.9949

Table S13. The temperature dependence of the isotope shifts observed for 6-I/6-I-*d*, given in ppm.

		$^{1}\Delta_{obs}$	$^{2}\Delta_{obs}$	$^{3}\Delta_{obs}$	$^{3}\Delta_{obs}$	$^{4}\Delta_{obs}$
T (K)	1/T(K^{-1})	δ_{C2D}-δ_{C2}	δ_{C3D}-δ_{C3}	δ_{C4D}-δ_{C4}	δ_{C6D}-δ_{C6}	δ_{C5D}-δ_{C5}
298	0.033	-0.362	-0.296	0.179	-0.459	0
283	0.035	-0.381	-0.311	0.203	-0.476	0
273	0.036	-0.384	-0.318	0.204	-0.49	0
263	0.038	-0.385	-0.323	0.205	-0.504	0
253	0.039	-0.391	-0.325	0.204	-0.514	0
243	0.041	-0.395	-0.333	0.201	-0.526	0
233	0.042	-0.401	-0.337	0.202	-0.539	0

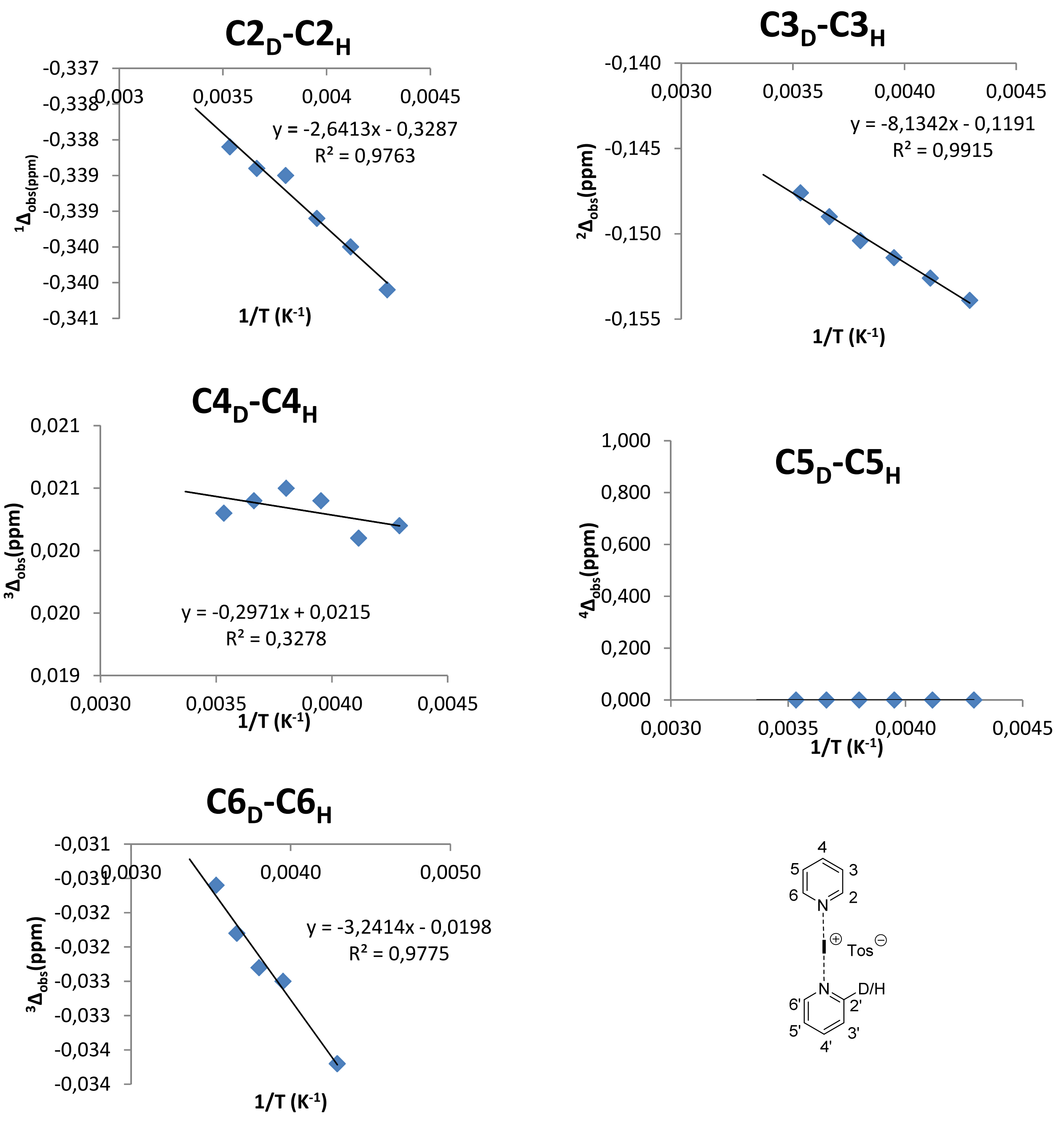

Figure S9. The temperature dependence of the isotope shifts of **6**/**6-*d***, shown for each carbon separately.

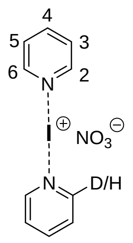

Table S12. The chemical shifts of [bis(pyridine)iodine] nitrate (7-I/7-I-*d*), given in ppm.

T (°C)	C-2/C-6 (H)	C-4 (H)	C-3/C-5 (H)	C-2 (D)	C-6 (D)	C-4 (D)	C-3 (D)	C-5 (D)
25	150.1714	142.5833	128.4541	149.8345	150.1404	142.6029	128.3067	128.4541
10	150.0323	142.5121	128.3682	149.6928	150.0019	142.5326	128.2194	128.3682
0	149.9164	142.4508	128.2965	149.5770	149.8852	142.4718	128.1465	128.2965
-10	149.8021	142.3892	128.2243	149.4614	149.7703	142.4099	128.0730	128.2243
-20	149.6875	142.3253	128.1498	149.3464	149.6552	142.3460	127.9972	128.1498
-30	149.5716	142.2578	128.0721	149.2301	149.5389	142.2786	127.9185	128.0721
-40	149.4533	142.1875	127.9909	149.1108	149.4201	142.2081	127.8362	127.9909

Table S13. The temperature dependence of the isotope shifts observed for 7-I/7-I-*d*, given in ppm.

T (K)	1/T (K^{-1})	$^{1}\Delta_{obs}$ δ_{C2D}-δ_{C2}	$^{2}\Delta_{obs}$ δ_{C3D}-δ_{C3}	$^{3}\Delta_{obs}$ δ_{C4D}-δ_{C4}	$^{3}\Delta_{obs}$ δ_{C6D}-δ_{C6}	$^{4}\Delta_{obs}$ δ_{C5D}-δ_{C5}
298	0.0034	-0.3369	-0.0310	0.0196	-0.1474	0.0000
283	0.0035	-0.3395	-0.0304	0.0205	-0.1488	0.0000
273	0.0037	-0.3394	-0.0312	0.0210	-0.1500	0.0000
263	0.0038	-0.3407	-0.0318	0.0207	-0.1513	0.0000
253	0.0040	-0.3411	-0.0323	0.0207	-0.1526	0.0000
243	0.0041	-0.3415	-0.0327	0.0208	-0.1536	0.0000
233	0.0043	-0.3425	-0.0332	0.0206	-0.1547	0.0000

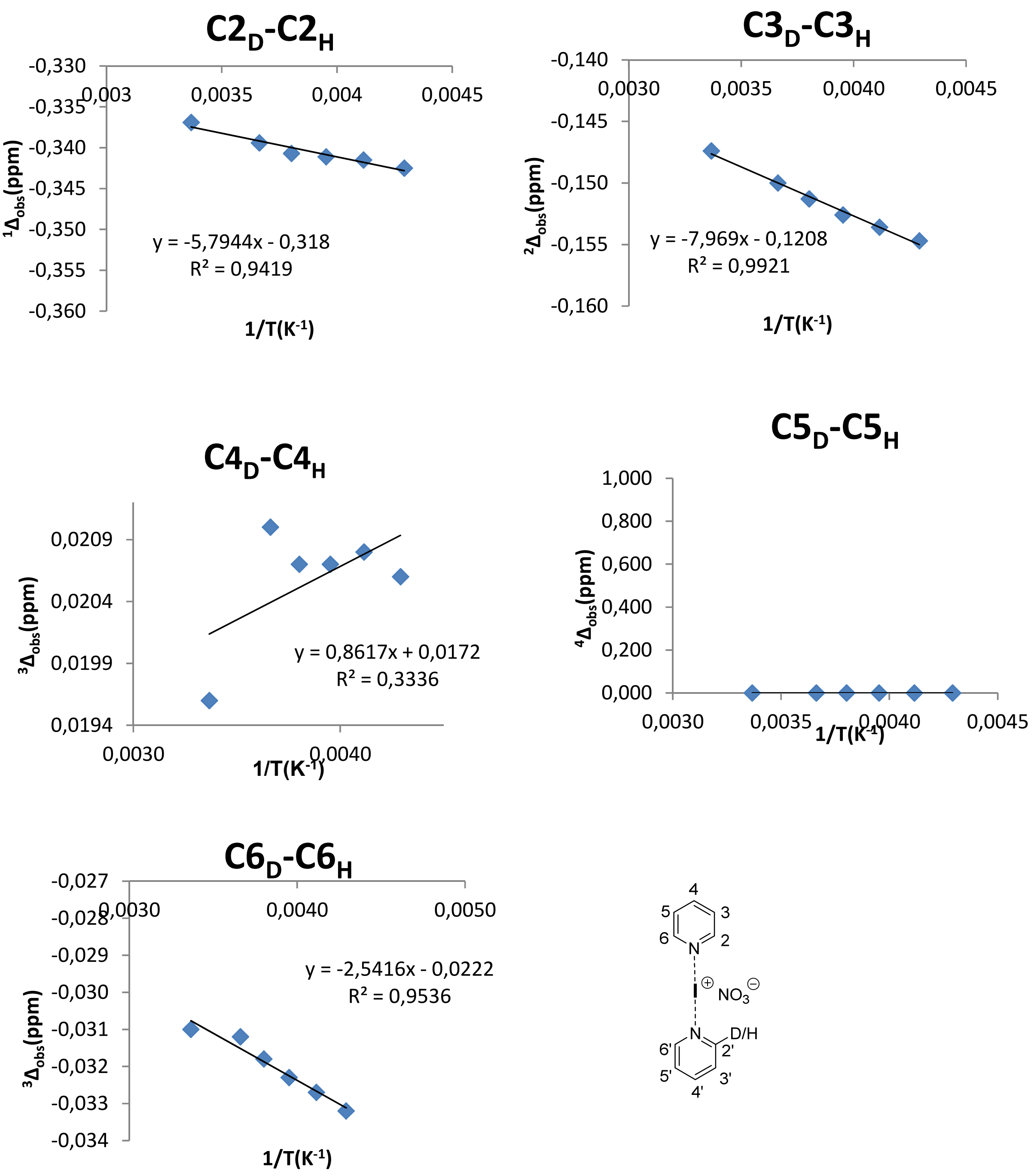

Figure S10. The temperature dependence of the isotope shifts of **7-I/7-I-*d***, shown for each carbon separately.

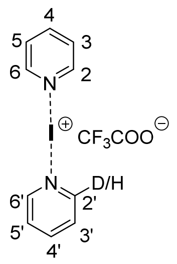

Table S14. The chemical shifts of [bis(pyridine)iodine] trifluoroacetate (8-I/8-I-*d*), given in ppm.

T (°C)	C-2/C-6 (H)	C-4 (H)	C-3/C-5 (H)	C-2 (D)	C-6 (D)	C-4 (D)	C-3 (D)	C-5 (D)
25	150.7248	141.8592	128.0830	150.3805	150.6988	141.8810	127.9336	128.0830
10	150.6298	141.8507	128.0464	150.2846	150.6030	141.8727	127.8953	128.0464
0	150.5696	141.8432	128.0212	150.2231	150.5421	141.8652	127.8690	128.0212
-10	150.4819	141.8291	127.9819	150.1345	150.4536	141.8515	127.8282	127.9819
-20	150.3983	141.8151	127.9430	150.0501	150.3694	141.8375	127.7880	127.9430
-30	150.3151	141.7992	127.9025	149.9658	150.2855	141.8216	127.7462	127.9025
-40	150.3285	141.8811	127.9584	149.9784	150.2982	141.9038	127.8009	127.9584

Table S15. The temperature dependence of the isotope shifts observed for 8-I/8-I-*d*, given in ppm.

		$^1\Delta_{obs}$	$^2\Delta_{obs}$	$^3\Delta_{obs}$	$^3\Delta_{obs}$	$^4\Delta_{obs}$
T (K)	1/T (K^{-1})	δ_{C2D}-δ_{C2}	δ_{C3D}-δ_{C3}	δ_{C4D}-δ_{C4}	δ_{C6D}-δ_{C6}	δ_{C5D}-δ_{C5}
298	0.0034	-0.3443	-0.0260	0.0218	-0.1494	0.0000
283	0.0035	-0.3452	-0.0268	0.0220	-0.1511	0.0000
273	0.0037	-0.3465	-0.0275	0.0220	-0.1522	0.0000
263	0.0038	-0.3474	-0.0283	0.0224	-0.1537	0.0000
253	0.0040	-0.3482	-0.0289	0.0224	-0.1550	0.0000
243	0.0041	-0.3493	-0.0296	0.0224	-0.1563	0.0000
233	0.0043	-0.3501	-0.0303	0.0227	-0.1575	0.0000

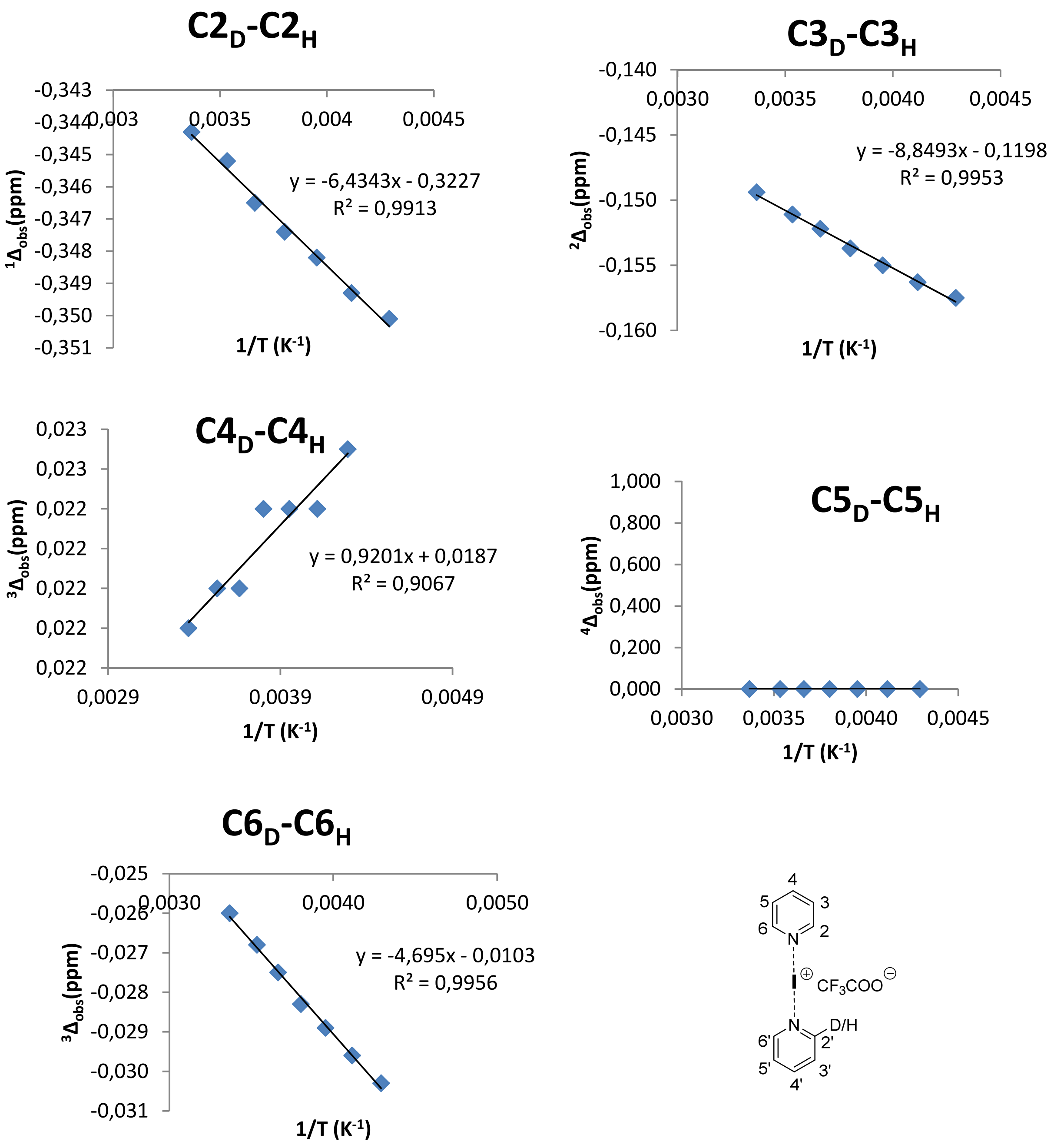

Figure S11. The temperature dependence of the isotope shifts of **8/8-*d*** shown for each carbon separately.

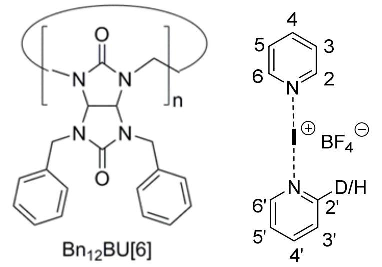

Table S16. The chemical shifts of [bis(pyridine)iodine] tetrafluoroborate with the counter ion scavanged (9-I/9-I-*d*) given in ppm.

T (°C)	C-2/C-6 (H)	C-4 (H)	C-3/C-5 (H)	C-2 (D)	C-6 (D)	C-4 (D)	C-3 (D)	C-5 (D)
25	149.7116	142.6578	128.4182	149.3741	149.6836	142.6788	128.2725	128.4182
10	149.5742	142.5625	128.3169	149.2343	149.5467	142.5838	128.1692	128.3169
0	149.4436	142.4601	128.2094	149.1023	149.4148	142.4813	128.0601	128.2094
-10	149.391	142.438	128.1818	149.0496	149.3611	142.4605	128.0312	128.1818
-20	149.2922	142.3706	128.1078	148.9500	149.2637	142.3923	127.9561	128.1078
-30	149.1897	142.2969	128.0282	148.8455	149.1595	142.317	127.8746	128.0282
-40	149.0841	142.218	127.9453	148.7405	149.0523	142.2404	127.791	127.9453

Table S17. The temperature dependence of the isotope shifts observed for 9-I/9-I-*d*, with the counterion trapped, given in ppm.

		$^1\Delta_{obs}$	$^2\Delta_{obs}$	$^3\Delta_{obs}$	$^3\Delta_{obs}$	$^4\Delta_{obs}$
T (K)	1/T(K^{-1})	δ_{C2D}-δ_{C2}	δ_{C3D}-δ_{C3}	δ_{C4D}-δ_{C4}	δ_{C6D}-δ_{C6}	δ_{C5D}-δ_{C5}
298	0.0034	-0.3375	-0.028	0.021	-0.1457	0
283	0.0035	-0.3399	-0.0275	0.0213	-0.1477	0
273	0.0037	-0.3413	-0.0288	0.0212	-0.1493	0
263	0.0038	-0.3414	-0.0299	0.0225	-0.1506	0
253	0.0040	-0.3422	-0.0285	0.0217	-0.1517	0
243	0.0041	-0.3442	-0.0302	0.0201	-0.1536	0
233	0.0043	-0.3436	-0.0318	0.0224	-0.1543	0

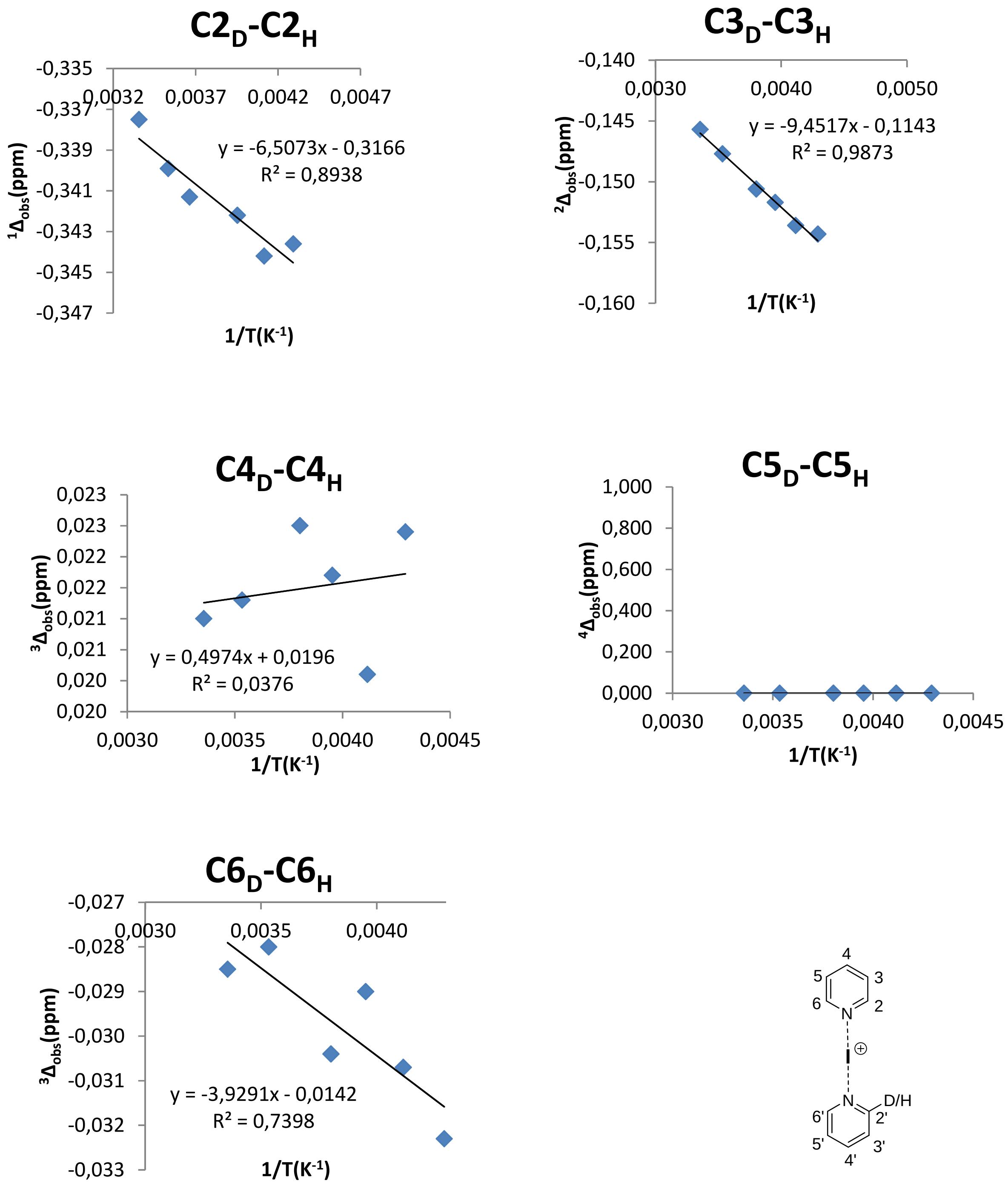

Figure S12. The temperature dependence of the isotope shifts of **9-I/9-I-*d***, shown for each carbon separately.

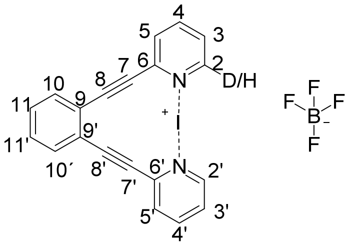

Table S18. The chemical shifts of [(1.2-bis(pyridin-2-ylethynyl)benzene)iodine] tetrafluoroborate (10-I/10-I-*d*), given in ppm.

T (°C)	C2(H)	C2(D)	C3(H)	C3(D)	C4(H)	C4(D)	C5/5(H/D)	C6(H)	C6(D)
30	151.3186	150.9878	127.0872	126.9450	142.7551	142.7779	130.9203	143.2181	143.1890
25	151.2671	150.9358	127.0561	126.9135	142.7277	142.7490	130.8937	143.1519	143.1224
20	151.2126	150.8805	127.0246	126.8815	142.6986	142.7208	130.8656	143.0817	143.0514
10	151.1025	150.7701	126.9590	126.8148	142.6392	142.6619	130.8077	142.9345	142.9052
0	151.0045	150.6712	126.9024	126.7569	142.5882	142.6096	130.7575	142.8015	142.7726
-10	150.8907	150.5552	126.8310	126.6841	142.5204	142.5392	130.6897	142.6539	142.6248

Table S19. The temperature dependence of the isotope shifts observed for (10-I/10-I-*d*), given in ppm.

		$^{1}\Delta_{obs}$	$^{2}\Delta_{obs}$	$^{3}\Delta_{obs}$	$^{3}\Delta_{obs}$
T (K)	1/T (K^{-1})	δ_{C2D}-δ_{C2}	δ_{C3D}-δ_{C3}	δ_{C4D}-δ_{C4}	δ_{C6D}-δ_{C6}
303	0.0033	-0.3308	-0.1422	0.0228	-0.0291
298	0.0034	-0.3313	-0.1426	0.0213	-0.0295
293	0.0034	-0.3321	-0.1431	0.0222	-0.0303
283	0.0035	-0.3318	-0.1442	0.0227	-0.0293
273	0.0037	-0.3326	-0.1455	0.0214	-0.0289
263	0.0038	-0.3357	-0.1469	0.0188	-0.0291

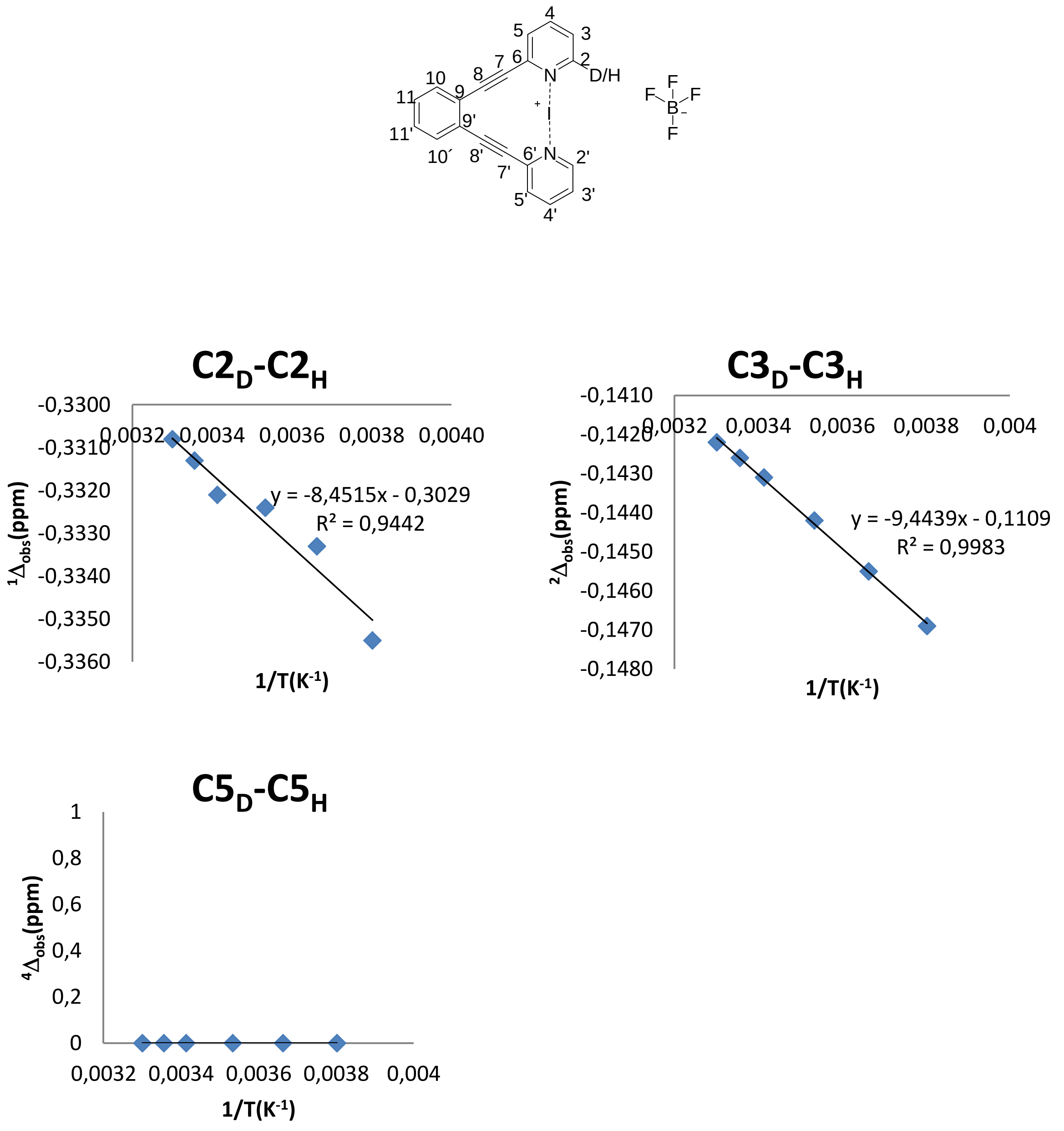

Figure S13. The temperature dependence of the isotope shifts of **10-I/10-I-*d***. Positions for which the temperature coefficients were not determinable are not shown.

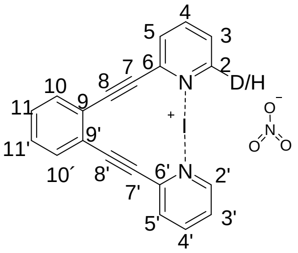

Table S20. The chemical shifts of [(1.2-bis(pyridine-2-ylethynyl)benzene)iodine] nitrate(12-I/12-I-*d*), given in ppm.

T (°C)	C-2(H)	C-2 (D)	C6H	C-6 (D)	C4(H)	C4(D)	C-3(H)	C-3 (D)	C5/5'
25	151.4838	151.1443	143.0984	-	142.6153	142.6363	127.0375	126.8934	130.8331
20	151.4448	151.2297	-	-	142.5989	142.6222	127.0228	126.8784	130.8074
10	151.3272	150.9886	142.9306	142.9033	142.5379	142.5604	126.9527	126.8062	130.7531
0	151.2062	150.8618	142.8152	142.785	142.4784	142.5003	126.8847	126.7378	130.6919
-5	151.1626	150.8215	142.7746	142.7475	142.4519	142.4755	126.8625	126.7137	130.6594
-10	151.0902	-	142.6852	142.655	142.4203	142.4431	126.8193	126.6703	130.6323
-20	150.9702	150.6305	142.557	142.5288	142.3647	142.3875	126.7544	126.604	130.5744
-30	150.8542	150.5062	142.4217	142.3888	142.3083	142.3314	126.6895	126.5381	130.513

Table S21. The temperature dependence of the isotope shifts observed for 12-I/12-I-*d* given in ppm.

T (K)	1/T(K^{-1})	$^{1}\Delta_{obs}$ δ_{C2D}-δ_{C2}	$^{2}\Delta_{obs}$ δ_{C3D}-δ_{C3}	$^{3}\Delta_{obs}$ δ_{C4D}-δ_{C4}
298	0.033	-0.3395	-0.1441	0.0210
293	0.034	-0.2151	-0.1444	0.0233
283	0.035	-0.3386	-0.1465	0.0225
273	0.036	-0.3444	-0.1469	0.0219
268	0.037	-0.3411	-0.1488	0.0236
263	0.038	n.d.	-0.1490	0.0228
253	0.039	-0.3397	-0.1504	0.0228
243	0.041	-0.3480	-0.1514	0.0231

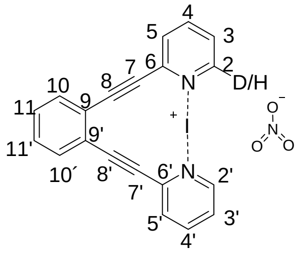

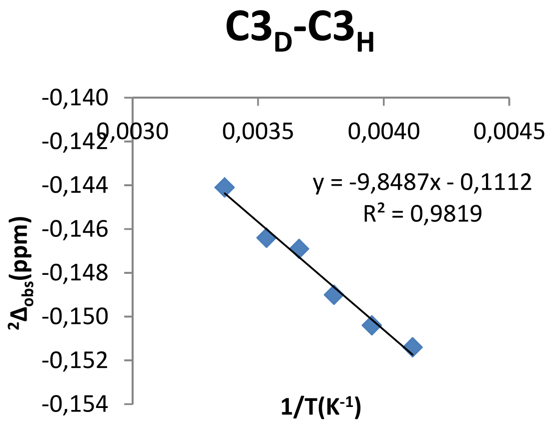

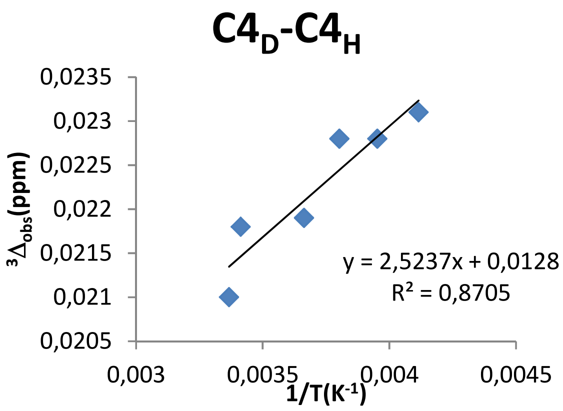

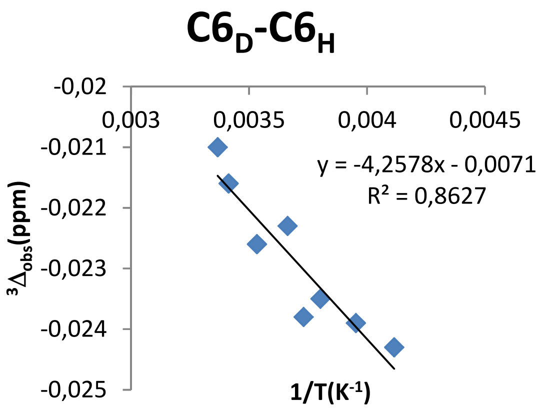

Figure S14. The temperature dependence of the isotope shifts of **12-I/12-I-*d***. Positions for which the temperature coefficients were not determinable are not shown.

Data for the temperature dependence of the isotope shifts of **11-I/11-I-*d*** and **11-H/11-I-*d*** are given in the supporting information of reference 1.

4. Computational Details: Geometry Optimization

Geometries were optimized with density functional theory (DFT), employing the B3LYP exchange and correlation functional.[6-10] in the thermochemical calculations, the LANL08d and LANL08f[11] basis set in conjunction with the LANL2DZ[12-14] effective core potential was used for I and Sb, respectively, LANL2DZ**Error! Bookmark not defined.**,**Error! Bookmark not defined.**,**Error! Bookmark not defined.** basis set for Ag, Pople's 6-311+G(d,p)[15-17] basis set for B, O, N, F and Cl, Pople's 6-311G(d,p)**Error! Bookmark not defined.**,**Error! Bookmark not defined.** basis set for the remaining atoms. For the calculation of chemical sheldings, single-point calculations at the geometries obtained were performed using the 6-311+G(d,p) basis set[18] for I, and Kutzelnigg's IGLO-III basis set[19] for the remaining atoms. Solvent effects were accounted for by the Polarizable Continuum Model (PCM),[20,21] with CH_2Cl_2 as solvent. The delocalized electrons in the 3-center-4-electron bonds make the DFT description of $[bis(pyridine)iodine]^+$ complexes challenging, owing on one hand to the incomplete description of nondynamic electron correlation in these bonds,[22] and on the other to the self-interaction error inherent to DFT.[23] However, reference calculations of closely related systems carried out in previous studies[1,2,24,25] with second-order Møller-Plesset perturbation theory (MP2)[26] confirm that DFT (B3LYP) provides a reasonable description of $[bis(pyridine)iodine]^+$ complexes. All calculations were performed using the Gaussian09 program package.[27]

4.1 Cartesian coordinates, energies and selected vibrational frequencies.

The optimized geometries corresponding to the global energy minimum of the complexes are shown. Energies are given in Hartree unit, coordinates in Ångström, vibrational frequencies in cm^{-1}. E(e) denotes the electronic energy (without ZPM), E(298), H(298) and G(298) the energy (including vibrational corrections), enthalpy and Gibbs free energy at 298.15K and 1 atm.

Py

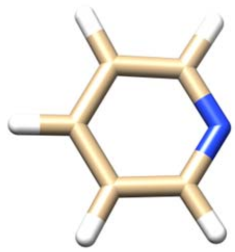

Point group: C2V

Charge: 0 Multiplicity: 1

N	0.000000	0.000000	1.417344
C	0.000000	1.144373	0.720490
C	0.000000	1.197244	-0.671483
C	0.000000	0.000000	-1.381707
C	0.000000	-1.197244	-0.671483
C	0.000000	-1.144373	0.720490
H	0.000000	2.060672	1.305056
H	0.000000	2.154356	-1.181332
H	0.000000	0.000000	-2.466700
H	0.000000	-2.154356	-1.181332
H	0.000000	-2.060672	1.305056

E(e)	-248.3548676360
ZPE	0.088294
E(T)	-248.262295
H(T)	-248.261351
G(T)	-248.293322

[Py-I]$^+$

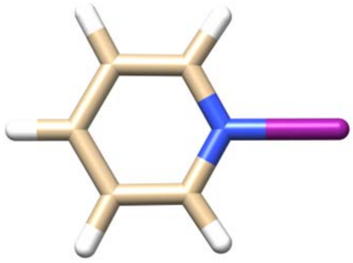

Point group: C2V

Charge: 1 Multiplicity: 1

I	0.000000	0.000000	1.534596
N	0.000000	0.000000	-0.557874
C	0.000000	1.183101	-1.216984
C	0.000000	1.201853	-2.598533
C	0.000000	0.000000	-3.301913
C	0.000000	-1.201853	-2.598533
C	0.000000	-1.183101	-1.216984
H	0.000000	2.084309	-0.616641

H	0.000000	2.159660	-3.105480
H	0.000000	0.000000	-4.386533
H	0.000000	-2.159660	-3.105480
H	0.000000	-2.084309	-0.616641

E(e)	-259.5374550910
ZPE	0.090226
E(T)	-259.441310
H(T)	-259.440366
G(T)	-259.478395

BF_4^-

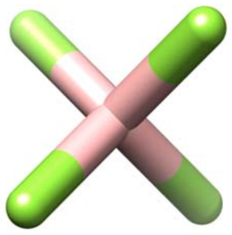

Point group: TD

Charge: -1 Multiplicity: 1

B	0.000000	0.000000	0.000000
F	0.816085	0.816085	0.816085
F	-0.816085	-0.816085	0.816085
F	0.816085	-0.816085	-0.816085
F	-0.816085	0.816085	-0.816085

E(e)	-424.7626066340
ZPE	0.013490
E(T)	-424.744655
H(T)	-424.743710
G(T)	-424.774444

ClO_4^-

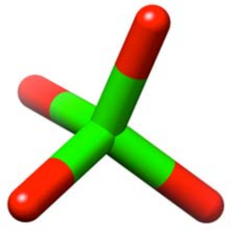

Point group: TD

Charge: -1 Multiplicity: 1

Cl	0.000000	0.000000	0.000000
O	0.865182	0.865182	0.865182
O	-0.865182	-0.865182	0.865182
O	0.865182	-0.865182	-0.865182

O	-0.865182	0.865182	-0.865182

E(e)	-761.0102913570
ZPE	0.013847
E(T)	-760.992195
H(T)	-760.991251
G(T)	-761.021712

PF_6^-

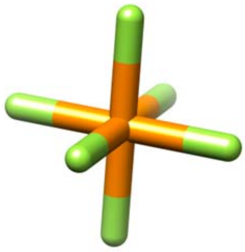

Point group: OH
Charge: -1 Multiplicity: 1

P	0.000000	0.000000	0.000000
F	0.000000	0.000000	1.642769
F	0.000000	0.000000	-1.642769
F	0.000000	1.642769	0.000000
F	0.000000	-1.642769	0.000000
F	-1.642769	0.000000	0.000000
F	1.642769	0.000000	0.000000

E(e)	-940.9713774160
ZPE	0.017897
E(T)	-940.947106
H(T)	-940.946162
G(T)	-940.980842

SbF_6^-

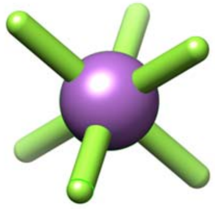

Point group: OH
Charge: -1 Multiplicity: 1

Sb	0.000000	0.000000	0.000000
F	0.000000	0.000000	1.921435
F	0.000000	0.000000	-1.921435
F	0.000000	1.921435	0.000000
F	0.000000	-1.921435	0.000000

F	-1.921435	0.000000	0.000000
F	1.921435	0.000000	0.000000

E(e)	-605.0085766110
ZPE	0.012332
E(T)	-604.987591
H(T)	-604.986647
G(T)	-605.027130

OTf⁻

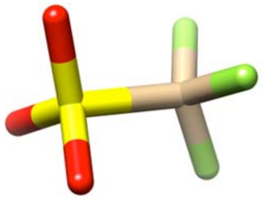

Point group: C3V
Charge: -1 Multiplicity: 1

S	-0.408296	-0.818319	0.000000
C	0.430946	0.863009	0.000000
O	-1.847428	-0.480862	0.000000
F	1.767357	0.731577	0.000000
F	0.082949	1.571243	1.086469
F	0.082949	1.571243	-1.086469
O	0.082949	-1.444039	-1.245695
O	0.082949	-1.444039	1.245695

E(e)	-961.7951718370
ZPE	0.026470
E(T)	-961.761457
H(T)	-961.760513
G(T)	-961.801377

OTs⁻

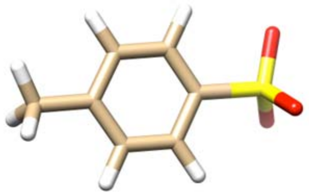

Point group: C1
Charge: -1 Multiplicity: 1

C	-2.654624	0.003364	0.007612
C	-1.930169	-1.197318	0.004836
C	-0.540674	-1.198501	-0.003943
C	0.153842	0.013118	-0.008285

C	-0.545267	1.215237	-0.003709
C	-1.940735	1.203849	0.004555
C	-4.164137	-0.009658	-0.004023
H	-2.462826	-2.143994	0.010304
H	0.003199	-2.136640	-0.007137
H	0.001048	2.150403	-0.007154
H	-2.479250	2.146940	0.009485
H	-4.561145	-0.724461	0.722017
H	-4.546961	-0.302391	-0.987620
H	-4.572386	0.975403	0.230312
S	1.966822	0.000762	-0.000496
O	2.367441	-0.832203	-1.171639
O	2.359449	-0.613546	1.301947
O	2.383079	1.426000	-0.123374

E(e)	-895.0975358930
ZPE	0.129523
E(T)	-894.957810
H(T)	-894.956866
G(T)	-895.006439

NO_3^-

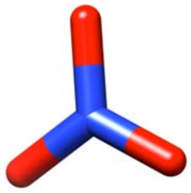

Point group: D3H
Charge: -1 Multiplicity: 1

N	0.000000	0.000000	0.000000
O	0.000000	1.258030	0.000000
O	1.089486	-0.629015	0.000000
O	-1.089486	-0.629015	0.000000

E(e)	-280.5454080200
ZPE	0.013716
E(T)	-280.528528
H(T)	-280.527584
G(T)	-280.555493

CF_3COO^-

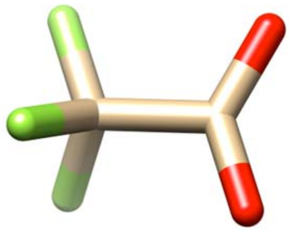

Point group: C1
Charge: -1 Multiplicity: 1

C	-0.523654	0.012460	-0.004687
F	-1.042414	-0.770660	-0.986444
F	-1.081357	1.234572	-0.163664
F	-1.009364	-0.476287	1.171400
C	1.054058	0.011083	-0.011414
O	1.537350	-1.135437	-0.005872
O	1.589625	1.131702	-0.006004

E(e)	-526.5243858030
ZPE	0.025415
E(T)	-526.492969
H(T)	-526.492025
G(T)	-526.529884

[Py-I-BF_4]

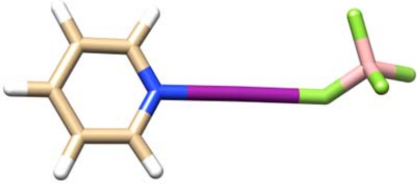

Point group: C1
Charge: 0 Multiplicity: 1

C	-4.213750	-1.008345	0.325480
C	-2.842923	-1.110049	0.170712
C	-4.812936	0.247962	0.288693
C	-4.018131	1.374941	0.096709
C	-2.651363	1.222673	-0.052645
N	-2.089290	-0.004725	-0.014031
H	-2.322840	-2.059828	0.190035
H	-4.793953	-1.911442	0.472392
H	-5.886253	0.347494	0.407967
H	-4.442373	2.371213	0.061299
H	-1.983512	2.061910	-0.203612
I	0.032111	-0.195375	-0.249755

B	3.544362	0.148900	0.211148
F	3.259173	1.503335	0.381550
F	2.432188	-0.438849	-0.574913
F	4.704140	-0.047210	-0.530418
F	3.589483	-0.520499	1.432418

E(e)	-684.3241900730
ZPE	0.104621
E(T)	-684.207083
H(T)	-684.206139
G(T)	-684.264802

[Py-I-ClO$_4$]

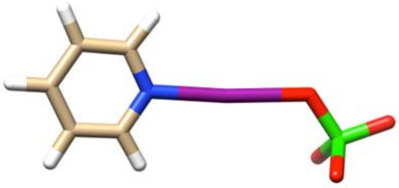

Point group: CS
Charge: 0 Multiplicity: 1

C	1.048432	4.478676	0.000000
C	1.175516	3.100096	0.000000
C	-0.224339	5.043399	0.000000
C	-1.338192	4.207986	0.000000
C	-1.153347	2.835729	0.000000
N	0.086008	2.306755	0.000000
H	2.139831	2.606272	0.000000
H	1.942028	5.091307	0.000000
H	-0.346440	6.121011	0.000000
H	-2.346278	4.604885	0.000000
H	-1.982106	2.137850	0.000000
I	0.334734	0.122143	0.000000
Cl	-0.334101	-3.315305	0.000000
O	0.781560	-2.210303	0.000000
O	0.385294	-4.605130	0.000000
O	-1.153347	-3.143365	1.221523
O	-1.153347	-3.143365	-1.221523

E(e)	-1020.5778480800
ZPE	0.105125
E(T)	-1020.460570
H(T)	-1020.459626
G(T)	-1020.516047

[Py-I-PF_6]

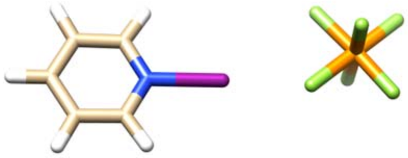

Point group: CS

Charge: 0 Multiplicity: 1

C	-1.058535	5.028059	0.000000
C	-1.191609	3.651807	0.000000
C	0.214705	5.591791	0.000000
C	1.328452	4.756233	0.000000
C	1.148456	3.385233	0.000000
N	-0.096631	2.859192	0.000000
H	-2.155288	3.157816	0.000000
H	-1.952412	5.640362	0.000000
H	0.337379	6.669340	0.000000
H	2.337217	5.151567	0.000000
H	1.975833	2.686376	0.000000
I	-0.330522	0.749848	0.000000
P	0.233817	-3.181034	0.000000
F	1.574430	-2.240893	0.000000
F	-0.708996	-1.759944	0.000000
F	0.214705	-3.145310	1.632685
F	1.133209	-4.535774	0.000000
F	0.214705	-3.145310	-1.632685
F	-1.150800	-4.042212	0.000000

E(e)	-1200.5274468100
ZPE	0.108930
E(T)	-1200.404011
H(T)	-1200.403067
G(T)	-1200.465359

[Py-I-SbF_6]

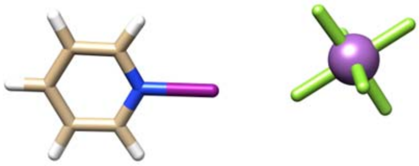

Point group: C1

Charge: 0 Multiplicity: 1

C	5.430346	1.508583	0.014901
C	4.068195	1.273695	-0.024756
C	6.308320	0.429767	0.077943
C	5.795167	-0.864511	0.099360
C	4.425939	-1.052734	0.057597
N	3.591675	0.008991	-0.003004
H	3.338202	2.072209	-0.073706

H	5.785751	2.531934	-0.004001
H	7.379598	0.595549	0.110114
H	6.441663	-1.732610	0.148248
H	3.970124	-2.034978	0.071561
I	1.493430	-0.312735	-0.061448
F	-0.980923	-0.743437	-0.129535
Sb	-2.758159	0.078064	0.017376
F	-4.483117	0.883774	0.163174
F	-1.937665	1.774962	-0.316279
F	-3.477668	-1.662047	0.343043
F	-2.980149	-0.183071	-1.863540
F	-2.441648	0.299807	1.890817

E(e)	-864.5654730300
ZPE	0.103403
E(T)	-864.445265
H(T)	-864.444321
G(T)	-864.513391

[Py-I-OTf]

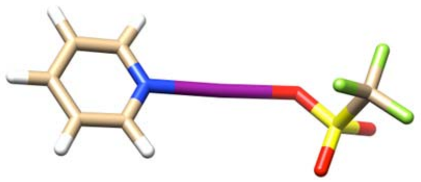

Point group: C1
Charge: 0 Multiplicity: 1

C	-5.069821	0.220049	-0.976241
C	-3.711250	-0.024253	-1.086796
C	-5.603113	0.536652	0.270344
C	-4.757248	0.599961	1.374406
C	-3.406457	0.345716	1.205573
N	-2.906688	0.040679	-0.007933
H	-3.242533	-0.275573	-2.030710
H	-5.691592	0.159648	-1.861422
H	-6.664388	0.731150	0.379978
H	-5.129979	0.842507	2.362335
H	-2.702808	0.379492	2.028813
I	-0.751409	-0.376362	-0.226668
S	2.701933	-0.703101	0.356863
O	2.320547	-0.542705	1.761806
O	3.779737	-1.638889	0.037501
O	1.511808	-0.889088	-0.576426
C	3.348857	0.982643	-0.170936
F	4.429025	1.297389	0.548556
F	3.678334	0.972249	-1.464560
F	2.411453	1.916545	0.027002

E(e)	-1221.3675796200
ZPE	0.117706
E(T)	-1221.234665
H(T)	-1221.233721

G(T)	-1221.297963

[Py-I-OTs]

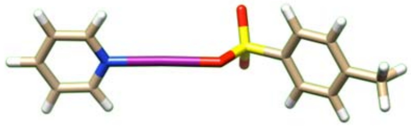

Point group: CS
Charge: 0 Multiplicity: 1

S	-1.067073	1.609080	0.000000
O	0.213185	0.717848	0.000000
C	-0.346571	3.253127	0.000000
O	-1.825270	1.433819	1.250841
O	-1.825270	1.433819	-1.250841
C	-0.061359	3.876985	-1.212629
C	-0.061359	3.876985	1.212629
C	0.515096	5.143069	-1.202940
C	0.515096	5.143069	1.202940
C	0.811316	5.795488	0.000000
C	1.408763	7.180470	0.000000
I	0.174977	-1.549358	0.000000
N	0.263693	-3.816433	0.000000
C	0.286313	-4.484550	1.167249
C	0.286313	-4.484550	-1.167249
C	0.333606	-5.869403	1.200541
C	0.333606	-5.869403	-1.200541
C	0.357463	-6.574167	0.000000
H	-0.293696	3.382559	-2.148263
H	-0.293696	3.382559	2.148263
H	0.737246	5.631275	-2.146752
H	0.737246	5.631275	2.146752
H	0.621017	7.941759	0.000000
H	2.024967	7.348202	-0.885814
H	2.024967	7.348202	0.885814
H	0.265038	-3.886510	2.070891
H	0.265038	-3.886510	-2.070891
H	0.350440	-6.377792	2.157153
H	0.350440	-6.377792	-2.157153
H	0.393670	-7.658106	0.000000

E(e)	-1154.6797250600
ZPE	0.220673
E(T)	-1154.441777
H(T)	-1154.440833
G(T)	-1154.510126

[Py-I-NO_3]

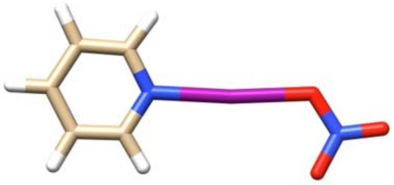

Point group: CS

Charge: 0 Multiplicity: 1

C	2.814510	-2.968988	0.000000
C	2.232869	-1.710986	0.000000
C	1.993323	-4.093345	0.000000
C	0.611340	-3.923786	0.000000
C	0.090296	-2.639477	0.000000
N	0.896041	-1.562006	0.000000
H	2.823274	-0.801954	0.000000
H	3.894536	-3.055047	0.000000
H	2.424552	-5.088457	0.000000
H	-0.064041	-4.770985	0.000000
H	-0.976927	-2.450483	0.000000
I	0.000000	0.517033	0.000000
N	-2.010753	2.960654	0.000000
O	-0.716005	2.681440	0.000000
O	-2.303369	4.152381	0.000000
O	-2.824680	2.040322	0.000000

E(e)	-540.1266908650
ZPE	0.105263
E(T)	-540.010600
H(T)	-540.009656
G(T)	-540.063098

[Py-I-CF_3COO]

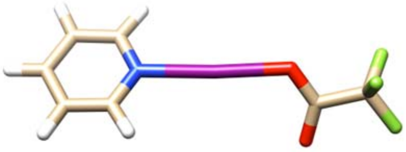

Point group: C1

Charge: 0 Multiplicity: 1

I	0.442184	-0.169266	0.127041
N	2.720852	-0.014851	0.020641
C	3.450782	-1.089545	-0.325518
C	4.832967	-1.023185	-0.410255
C	3.323228	1.155864	0.293348
C	4.701620	1.288181	0.226702
C	5.469558	0.183075	-0.130427
H	2.904008	-2.002053	-0.533396
H	5.392101	-1.907392	-0.692096
H	6.549655	0.260983	-0.190349

H	2.677603	1.983186	0.564760
H	5.155995	2.245591	0.452088
C	-4.076612	-0.019295	0.004250
F	-4.333101	-1.286316	-0.386245
F	-4.942876	0.781897	-0.633979
F	-4.350539	0.058894	1.325212
C	-2.592162	0.377875	-0.286962
O	-1.790250	-0.431316	0.309258
O	-2.357092	1.339646	-0.990560

E(e)	-786.1118235740
ZPE	0.117172
E(T)	-785.980844
H(T)	-785.979900
G(T)	-786.041024

[(Py-I-Py)BF_4] (1-I)

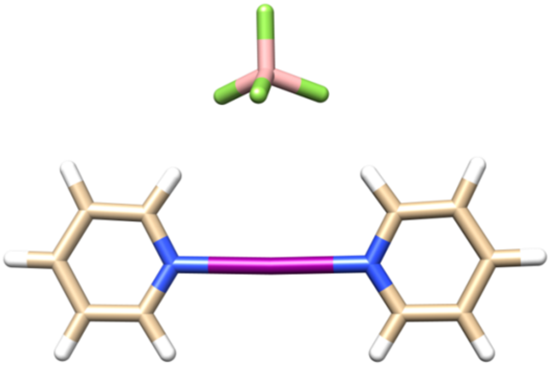

Point group: C1

Charge: 0 Multiplicity: 1

C	-4.388248	-2.158246	0.117797
C	-3.002219	-2.171066	0.118837
C	-5.050702	-0.941482	-0.027232
C	-4.304459	0.224958	-0.167175
C	-2.918929	0.146695	-0.159070
N	-2.294561	-1.036425	-0.017766
H	-2.436223	-3.089120	0.229352
H	-4.930659	-3.089461	0.229685
H	-6.134673	-0.904096	-0.030760
H	-4.779786	1.191876	-0.281962
H	-2.290615	1.024369	-0.265213
I	0.006180	-1.069306	-0.012503
N	2.306835	-1.021842	-0.016952
C	2.923401	0.168416	-0.129854
C	4.308391	0.255878	-0.136955
C	3.022046	-2.154842	0.091272
C	4.407957	-2.132963	0.089606
C	5.062350	-0.908716	-0.026047
H	2.289449	1.044368	-0.214872
H	4.777297	1.228380	-0.228629
H	6.146053	-0.864180	-0.029476
H	2.462334	-3.079161	0.179371
H	4.956487	-3.063106	0.178173

B	-0.020533	3.387190	0.066191
F	-0.037666	4.791226	-0.043768
F	-1.145208	2.847305	-0.609039
F	1.163817	2.881675	-0.528808
F	-0.061832	3.010180	1.424058

E(e)	-932.7082387840
ZPE	0.194795
E(T)	-932.494832
H(T)	-932.493887
G(T)	-932.567729

Frequencies:

Stretch (sym)	167.92
Stretch (asym)	165.75
Twist	34.02

[(Py-I-Py)ClO$_4$] (2-I)

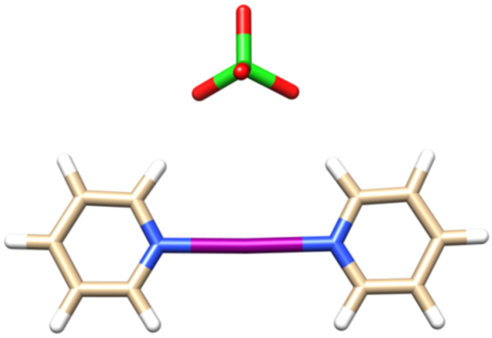

Point group: C1

Charge: 0 Multiplicity: 1

C	4.427961	-2.298839	0.101770
C	3.042248	-2.329944	0.108712
C	5.073672	-1.072601	-0.038975
C	4.311605	0.084769	-0.168708
C	2.927328	-0.011859	-0.154985
N	2.319370	-1.203769	-0.017949
H	2.488827	-3.256053	0.215758
H	4.983047	-3.223483	0.205733
H	6.157037	-1.020962	-0.047046
H	4.773663	1.058558	-0.279503
H	2.286062	0.857773	-0.251232
I	0.019382	-1.266241	-0.010205
N	-2.281539	-1.251430	-0.018534
C	-2.914116	-0.078832	-0.202470
C	-4.300087	-0.011363	-0.218083
C	-2.980770	-2.386170	0.155128
C	-4.366855	-2.383955	0.149045
C	-5.037935	-1.178055	-0.040544
H	-2.291245	0.799587	-0.333182
H	-4.782191	0.947511	-0.367028
H	-6.122139	-1.149153	-0.049012
H	-2.407853	-3.295197	0.299425
H	-4.902704	-3.314786	0.291561
Cl	-0.052732	3.387873	0.062839

O	-0.061162	4.849320	-0.248778
O	-0.122147	3.185671	1.543049
O	-1.234659	2.731232	-0.588801
O	1.208416	2.771354	-0.468327

E(e)	-1268.9565953200
ZPE	0.195438
E(T)	-1268.742852
H(T)	-1268.741907
G(T)	-1268.814682

Frequencies:

Stretch (sym)	167.38
Stretch (asym)	166.03
Twist	39.28

[(Py-I-Py)PF$_6$] (3-I)

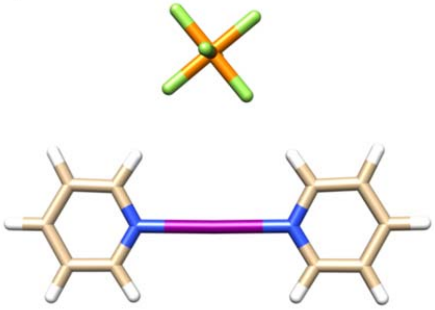

Point group: C1

Charge: 0 Multiplicity: 1

C	-2.074656	4.798336	-0.000449
C	-2.309779	3.432250	-0.000276
C	-0.758957	5.255611	-0.000542
C	0.281296	4.330764	-0.000440
C	-0.019919	2.976443	-0.000195
N	-1.295365	2.550286	-0.000074
H	-3.312830	3.020819	-0.000183
H	-2.913484	5.484141	-0.000514
H	-0.547991	6.319509	-0.000742
H	1.318785	4.643336	-0.000524
H	0.751533	2.215992	-0.000140
I	-1.719083	0.288520	-0.000122
N	-2.097236	-1.982869	-0.000247
C	-1.044764	-2.819835	-0.000959
C	-1.224771	-4.195446	-0.001010
C	-3.352096	-2.464616	0.000263
C	-3.598481	-3.828806	-0.000015
C	-2.518674	-4.708703	-0.000636
H	-0.060972	-2.367217	-0.001113
H	-0.356803	-4.844089	-0.001407
H	-2.684266	-5.780600	-0.000760
H	-4.154254	-1.735267	0.000765
H	-4.621301	-4.186288	0.000366
P	3.356214	-0.561872	0.000505
F	4.145153	-1.999823	0.001670
F	3.347686	-0.559005	1.641128

F	1.910667	-1.358535	0.000787
F	3.348050	-0.560793	-1.640169
F	4.787972	0.236496	0.000318
F	2.551895	0.879246	-0.000294

E(e)	-1448.9162397900
ZPE	0.199392
E(T)	-1448.696382
H(T)	-1448.695438
G(T)	-1448.772126

Frequencies:

Stretch (sym)	166.75
Stretch (asym)	163.76
Twist	30.40

[(Py-I-Py)SbF$_6$] (4-I)

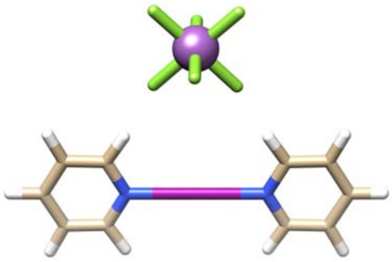

Point group: C1
Charge: 0 Multiplicity: 1

C	-3.498523	4.368491	0.029690
C	-3.470462	2.982800	0.029741
C	-2.294008	5.067065	-0.004961
C	-1.098411	4.355452	-0.038505
C	-1.136917	2.968335	-0.036756
N	-2.308032	2.307908	-0.003095
H	-4.378025	2.390332	0.056359
H	-4.451826	4.882683	0.056741
H	-2.288290	6.151655	-0.005621
H	-0.139265	4.858795	-0.065831
H	-0.233895	2.370075	-0.061438
I	-2.317441	0.005176	-0.002383
N	-2.324002	-2.297523	-0.002744
C	-1.156787	-2.965282	-0.025262
C	-1.126852	-4.352596	-0.027090
C	-3.490916	-2.965047	0.018721
C	-3.527556	-4.350562	0.018049
C	-2.327155	-5.056683	-0.005256
H	-0.250281	-2.372151	-0.041162
H	-0.170647	-4.861920	-0.045406
H	-2.328222	-6.141290	-0.006265
H	-4.394892	-2.366798	0.036585
H	-4.484221	-4.858875	0.035723
Sb	3.025265	-0.006805	0.004360
F	3.103132	-0.029792	-1.912213
F	4.346472	-1.392859	0.081892

F	1.635813	-1.333661	-0.042194
F	1.684504	1.367874	-0.072843
F	4.395817	1.331747	0.051102
F	2.928169	0.016214	1.920360

E(e)	-1112.9546191200
ZPE	0.193741
E(T)	-1112.738062
H(T)	-1112.737118
G(T)	-1112.820987

Frequencies:

Stretch (sym)	1166.49
Stretch (asym)	164.28
Twist	40.84

[(Py-I-Py)OTf] (5-I)

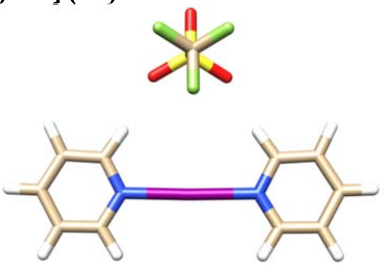

Point group: C1
Charge: 0 Multiplicity: 1

C	3.716949	-3.580224	0.267762
C	2.357028	-3.312927	0.274206
C	4.603021	-2.556309	-0.060017
C	4.099378	-1.297109	-0.372388
C	2.726785	-1.091093	-0.348989
N	1.885706	-2.090981	-0.028585
H	1.622937	-4.071573	0.521211
H	4.066732	-4.575085	0.516368
H	5.672065	-2.739246	-0.071849
H	4.753563	-0.473721	-0.633492
H	2.281987	-0.129205	-0.585477
I	-0.372376	-1.655110	-0.007486
N	-2.615398	-1.143859	-0.006627
C	-2.986599	0.117574	-0.290635
C	-4.327397	0.477040	-0.307720
C	-3.536214	-2.084429	0.266446
C	-4.889762	-1.786605	0.264930
C	-5.292703	-0.485244	-0.026846
H	-2.194574	0.829266	-0.503085
H	-4.597635	1.500348	-0.539935
H	-6.345932	-0.226189	-0.035167
H	-3.166605	-3.079837	0.485552
H	-5.608259	-2.566061	0.488782

S	0.590252	2.869618	-0.857518
O	0.858871	4.196408	-1.443231
O	-0.807255	2.400979	-0.993651
O	1.616419	1.842526	-1.146530
C	0.773090	3.156082	0.992488
F	-0.113887	4.065146	1.422402
F	0.566007	2.016283	1.673384
F	2.004517	3.598826	1.285604

E(e)	-1469.7430712400
ZPE	0.208007
E(T)	-1469.513764
H(T)	-1469.512820
G(T)	-1469.593140

Frequencies:

Stretch (sym)	167.84
Stretch (asym)	165.83
Twist	39.33

[(Py-I-Py)OTs] (6-I)

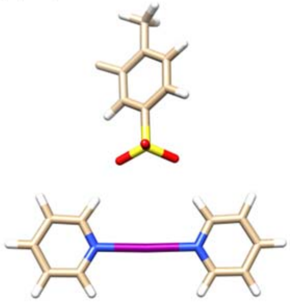

Point group: C1

Charge: 0 Multiplicity: 1

C	-3.530627	4.426565	0.050301
C	-3.558780	3.041226	0.053120
C	-2.303979	5.074239	-0.081705
C	-1.146000	4.313475	-0.207148
C	-1.236794	2.927451	-0.198136
N	-2.430631	2.320018	-0.069234
H	-4.484455	2.485930	0.154123
H	-4.456614	4.979964	0.151194
H	-2.253810	6.157760	-0.085809
H	-0.172134	4.777221	-0.310615
H	-0.360245	2.288479	-0.290652
I	-2.498056	0.019154	-0.051953
N	-2.506410	-2.283297	-0.034453
C	-1.329793	-2.933784	-0.094527
C	-1.287801	-4.322085	-0.082558
C	-3.664068	-2.963180	0.038125
C	-3.685018	-4.348597	0.052800

C	-2.477016	-5.040322	-0.008306
H	-0.428066	-2.326851	-0.152880
H	-0.327127	-4.821022	-0.130953
H	-2.465137	-6.124888	0.002213
H	-4.573270	-2.374411	0.084781
H	-4.633997	-4.868059	0.111906
C	6.421061	-0.049234	-0.153140
C	5.593363	-0.247509	-1.268407
C	4.209603	-0.241744	-1.146580
C	3.626296	-0.035910	0.105612
C	4.427567	0.161826	1.224213
C	5.816628	0.154157	1.089309
C	7.923391	-0.067828	-0.300010
H	6.040982	-0.409142	-2.244895
H	3.584502	-0.402446	-2.017845
H	3.963092	0.315813	2.190615
H	6.436333	0.308681	1.967522
H	8.255058	0.634319	-1.070593
H	8.278398	-1.060585	-0.595059
H	8.417638	0.198974	0.635951
S	1.824336	-0.006996	0.253836
O	1.384931	1.198034	-0.509733
O	1.526585	0.078618	1.704892
O	1.358176	-1.278211	-0.372512

E(e)	-1403.0367138900
ZPE	0.311193
E(T)	-1402.701376
H(T)	-1402.700432
G(T)	-1402.787430

Frequencies:

Stretch (sym.)	167.10
Stretch (asym.)	165.24
Twist	46.57

[(Py-I-Py)NO_3] (7-I)

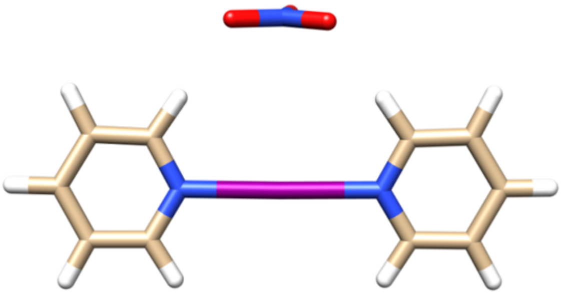

Point group: C1

Charge: 0 Multiplicity: 1

C	4.327722	0.474144	0.187685
C	2.940255	0.427123	0.181794
C	5.048444	-0.704264	0.019350
C	4.359417	-1.902918	-0.151938
C	2.973670	-1.885188	-0.149761

N	2.290949	-0.738841	0.015079
H	2.331023	1.317661	0.310730
H	4.824231	1.427699	0.322975
H	6.132994	-0.690481	0.020933
H	4.881074	-2.842919	-0.286748
H	2.387226	-2.787639	-0.280515
I	-0.011764	-0.747878	0.011914
N	-2.314315	-0.717387	0.013636
C	-3.011300	-1.864406	-0.064798
C	-4.397199	-1.864997	-0.064514
C	-2.949229	0.465409	0.094733
C	-4.335954	0.530047	0.098188
C	-5.071306	-0.648559	0.017884
H	-2.436704	-2.781624	-0.128032
H	-4.930614	-2.805877	-0.128566
H	-6.155606	-0.621295	0.019301
H	-2.328332	1.354767	0.157934
H	-4.820457	1.497051	0.163509
N	0.051967	3.183450	-0.067639
O	1.046557	3.047069	0.693072
O	0.219659	3.369592	-1.295589
O	-1.112301	3.123503	0.409734

E(e)	-788.4924568160
ZPE	0.195209
E(T)	-788.280141
H(T)	-788.279197
G(T)	-788.348962

Frequencies:

Stretch (sym)	167.92
Stretch (asym)	164.89
Twist	28.39

[(Py-I-Py)CF_3COO] (8-I)

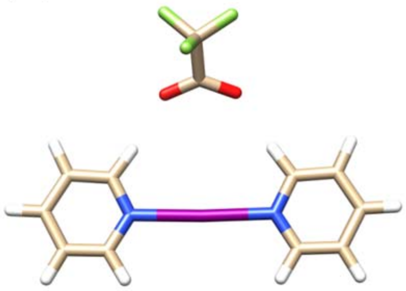

Point group: C1
Charge: 0 Multiplicity: 1

C	-3.657876	-3.512780	0.259279
C	-2.302820	-3.222311	0.271205
C	-4.562232	-2.494391	-0.035067
C	-4.081292	-1.217809	-0.309484
C	-2.712096	-0.987301	-0.283183
N	-1.854047	-1.983312	0.004814
H	-1.554906	-3.975198	0.493062
H	-3.990223	-4.520551	0.477992

H	-5.628055	-2.695356	-0.049935
H	-4.750612	-0.398291	-0.543074
H	-2.282196	-0.009089	-0.490364
I	0.390533	-1.489027	0.022685
N	2.614016	-0.894803	0.011940
C	2.928203	0.383201	-0.268054
C	4.253357	0.797073	-0.298509
C	3.576016	-1.798029	0.268165
C	4.916096	-1.444984	0.252198
C	5.260939	-0.126199	-0.035900
H	2.101804	1.061922	-0.464567
H	4.479037	1.832147	-0.526079
H	6.302447	0.176189	-0.055137
H	3.250431	-2.809015	0.485936
H	5.668863	-2.195449	0.462146
C	-1.058075	3.976912	0.254394
F	-2.212543	4.534897	-0.175585
F	-0.120001	4.946253	0.202603
F	-1.237147	3.678403	1.570302
C	-0.672756	2.683405	-0.542721
O	-1.646742	1.962365	-0.835569
O	0.545224	2.530285	-0.749354

E(e)	-1034.4611934400
ZPE	0.207284
E(T)	-1034.233982
H(T)	-1034.233038
G(T)	-1034.309829

Frequencies:

Stretch (sym)	168.64
Stretch (asym)	167.03
Twist	41.93

[Py-I-Py]$^+$ (refers to 9-I)

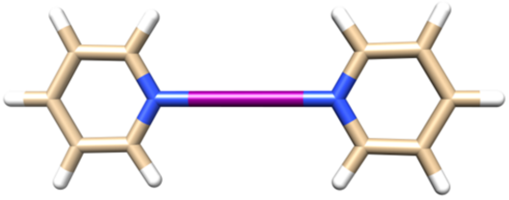

Point group: D2

Charge: 1 Multiplicity: 1

I	0.000000	0.000000	0.000000
N	0.000000	0.000000	2.303636
C	-0.798422	0.850204	2.972273
C	-0.822068	0.875281	4.358227

C	0.000000	0.000000	5.063163
C	0.822068	-0.875281	4.358227
C	0.798422	-0.850204	2.972273
N	0.000000	0.000000	-2.303636
C	0.798422	0.850204	-2.972273
C	0.822068	0.875281	-4.358227
C	0.000000	0.000000	-5.063163
C	-0.822068	-0.875281	-4.358227
C	-0.798422	-0.850204	-2.972273
H	-1.417644	1.509766	2.374876
H	-1.476845	1.572376	4.867416
H	0.000000	0.000000	6.147749
H	1.476845	-1.572376	4.867416
H	1.417644	-1.509766	2.374876
H	1.417644	1.509766	-2.374876
H	1.476845	1.572376	-4.867416
H	0.000000	0.000000	-6.147749
H	-1.476845	-1.572376	-4.867416
H	-1.417644	-1.509766	-2.374876

E(e)	-507.9339526920
ZPE	0.180408
E(T)	-507.741647
H(T)	-507.740703
G(T)	-507.793976

Frequencies:

Stretch(sym)	165.78
Stretch (asym)	165.36
Twist:	20.84

[(Py-Ag-Py)BF_4] (1-Ag),. DFT,. CH_2Cl_2

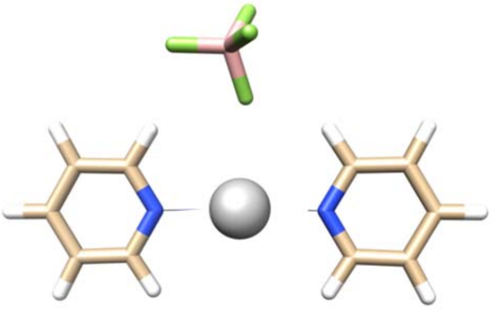

C	4.307237	0.402228	-0.107096
C	2.921964	0.298711	-0.102503
C	5.071550	-0.756556	-0.014300
C	4.420352	-1.983495	0.079748
C	3.032662	-2.007361	0.078365
N	2.290245	-0.887219	-0.010528
H	2.287845	1.173897	-0.173729
H	4.769005	1.379692	-0.182371
H	6.155144	-0.705543	-0.015129
H	4.972578	-2.912941	0.153920
H	2.490814	-2.943350	0.150550

Ag	0.105519	-0.951168	-0.005972
N	-2.072729	-1.123559	-0.006347
C	-2.686324	-2.321940	0.031896
C	-4.067955	-2.452361	0.025989
C	-2.832091	-0.011520	-0.051361
C	-4.220546	-0.063355	-0.059928
C	-4.851577	-1.302451	-0.021094
H	-2.043798	-3.194139	0.068073
H	-4.513443	-3.439693	0.057996
H	-5.934138	-1.372176	-0.026891
H	-2.308634	0.936229	-0.077699
H	-4.787186	0.859687	-0.096245
B	-0.344727	3.187095	0.036551
F	-1.751974	3.162231	0.167490
F	0.245720	3.482435	1.280263
F	0.095308	1.901176	-0.395612
F	0.031146	4.148805	-0.918889

E(e)	=	-1067.1639118
ZPE		0.194345
E(298)		-1066.950620
H(298)		-1066.949676
G(298)		-1067.024787

Frequencies:

Stretch (sym)	136.87
Stretch (asym)	227.72
Twist	22.12

[(Py-Ag-Py)ClO$_4$] (2-Ag),. DFT,. CD$_2$Cl$_2$

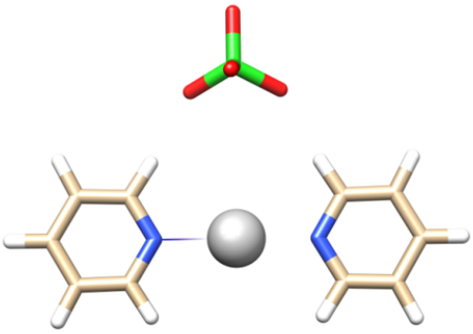

Charge = 0 Multiplicity = 0

C	4.299897	-2.405552	0.121506
C	2.912400	-2.382670	0.106218
C	4.993157	-1.204789	-0.005241
C	4.269200	-0.024871	-0.142653
C	2.880889	-0.080752	-0.149293
N	2.209420	-1.242061	-0.026916
H	2.338526	-3.297247	0.203006
H	4.819578	-3.350069	0.231585
H	6.077817	-1.190169	0.003755
H	4.763966	0.934049	-0.242953
H	2.283169	0.817464	-0.250968
Ag	0.020805	-1.252065	-0.033342

N	-2.168437	-1.291128	-0.021283
C	-2.844100	-2.442064	0.156739
C	-4.230763	-2.498017	0.168374
C	-2.867407	-0.152178	-0.192307
C	-4.256622	-0.129957	-0.192531
C	-4.952330	-1.320720	-0.009992
H	-2.248711	-3.337702	0.292712
H	-4.727979	-3.449589	0.315270
H	-6.037057	-1.332248	-0.005747
H	-2.290629	0.755328	-0.327124
H	-4.774013	0.811807	-0.333165
Cl	-0.055142	3.371847	0.060192
O	-0.075563	4.826339	-0.279816
O	-0.124141	3.197460	1.544478
O	-1.231002	2.691125	-0.578460
O	1.210971	2.753299	-0.457460

E(e)	-1403.4127457
ZPE	0.195071
E(298)	-1403.199054
H(298)	-1403.198110
G(298)	-1403.272611

Frequencies:

Stretch (sym)	138.25
Stretch (asym)	231.28
Twist	43.93

[(Py-Ag-Py)PF_6] (3-Ag)

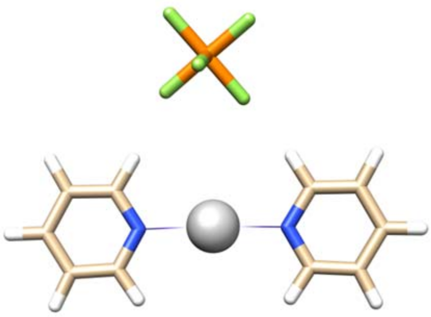

Charge = 0 Multiplicity = 0

C	0.435106	4.286980	-0.049103
C	0.098001	2.939574	-0.073263
C	-0.579074	5.235592	0.033423
C	-1.900359	4.800474	0.088543
C	-2.158832	3.437199	0.059853
N	-1.179462	2.516261	-0.019715
H	0.861341	2.174572	-0.137451
H	1.478528	4.576127	-0.094179
H	-0.345100	6.294627	0.054513
H	-2.724439	5.501169	0.153391
H	-3.174845	3.061599	0.101689
Ag	-1.646938	0.373672	-0.037358
N	-2.218874	-1.741777	-0.009987

C	-1.282931	-2.708991	-0.053343
C	-1.609518	-4.059104	-0.036475
C	-3.514505	-2.103348	0.052656
C	-3.918348	-3.430931	0.073419
C	-2.949045	-4.429149	0.027802
H	-0.251889	-2.382940	-0.102121
H	-0.819037	-4.799447	-0.073208
H	-3.233396	-5.475889	0.042340
H	-4.240822	-1.299328	0.086608
H	-4.974214	-3.669373	0.124372
P	3.237484	-0.737669	0.016436
F	1.763413	-1.449275	-0.200257
F	2.905119	-0.554469	1.614121
F	2.555158	0.733421	-0.286602
F	3.551190	-0.916254	-1.583587
F	3.901081	-2.205861	0.317448
F	4.694381	-0.019767	0.230004

E(e)	-1583.3707465
ZPE	0.198800
E(298)	-1583.151040
H(298)	-1583.150096
G(298)	-1583.230303

Frequencies:

Stretch (sym)	140.26
Stretch (asym)	234.40
Twist	29.41

[(Py-Ag-Py)SbF$_6$] (4-Ag)

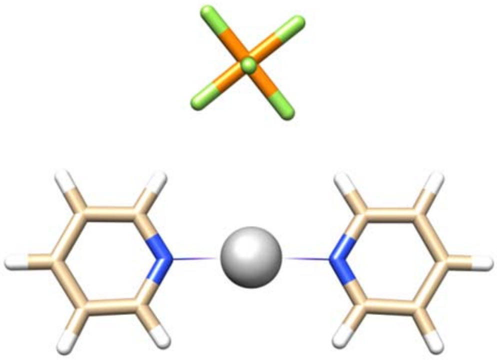

Charge = 0 Multiplicity = 0

C	1.110356	-4.288230	-0.268942
C	1.111499	-2.898933	-0.262376
C	2.300846	-4.966980	-0.029771
C	3.456663	-4.228550	0.209145
C	3.380119	-2.842929	0.199859
N	2.229256	-2.183140	-0.031964
H	0.202379	-2.338508	-0.441961
H	0.184714	-4.818931	-0.458433

H	2.328489	-6.051410	-0.028683
H	4.407377	-4.711861	0.401116
H	4.258898	-2.234959	0.382182
Ag	2.178896	0.008445	-0.032181
N	2.206884	2.200925	-0.031870
C	1.083266	2.904022	-0.272654
C	1.066341	4.293185	-0.279781
C	3.348003	2.873846	0.210038
C	3.408712	4.260268	0.219497
C	2.246828	4.985487	-0.030112
H	0.182240	2.333137	-0.459949
H	0.136447	4.813239	-0.477742
H	2.262121	6.070161	-0.029102
H	4.231925	2.275964	0.400645
H	4.352093	4.754368	0.419950
Sb	-2.780310	-0.011050	0.042508
F	-3.920105	-0.027606	-1.498421
F	-3.852353	-1.369518	0.868153
F	-3.855749	1.360316	0.841965
F	-1.682150	1.330387	-0.792358
F	-1.678159	-1.365316	-0.765722
F	-1.599015	0.005500	1.559049

E(e)	-1247.3757758
ZPE	0.1983259
E(298)	-1247.159247
H(298)	-1247.158303
G(298)	-1247.244743

Frequencies:

Stretch (sym)	140.10
Stretch (asym)	233.10
Twist	47.13

[(Py-Ag-Py)OTf] (5-Ag)

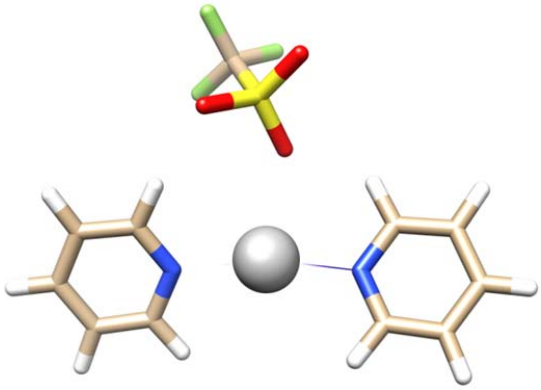

Charge = 0 Multiplicity = 0

C	-4.199623	-1.952463	-0.092861
C	-2.982595	-1.281329	-0.086569
C	-5.378122	-1.213743	-0.061103
C	-5.297071	0.175764	-0.024100
C	-4.043694	0.773178	-0.020038

N	-2.902161	0.061312	-0.050842
H	-2.037571	-1.810453	-0.110121
H	-4.212658	-3.035758	-0.121431
H	-6.342544	-1.710485	-0.064679
H	-6.187024	0.793639	0.001892
H	-3.941153	1.852082	0.009035
Ag	-0.908624	1.048784	-0.030909
N	0.803747	2.463593	0.010219
C	1.994639	2.083897	0.510815
C	3.086974	2.942524	0.557335
C	0.675447	3.718329	-0.459963
C	1.720400	4.632245	-0.448683
C	2.950866	4.238341	0.070169
H	2.062948	1.066127	0.878859
H	4.024976	2.589983	0.970215
H	3.787000	4.929151	0.093518
H	-0.296599	3.989728	-0.855953
H	1.564965	5.630636	-0.840476
16	1.257418	-2.024380	0.636987
O	1.978132	-1.082368	1.518875
O	0.839115	-3.288697	1.268005
O	0.221604	-1.385053	-0.215234
C	2.561171	-2.546403	-0.611218
F	3.595954	-3.135115	0.003561
F	2.049131	-3.411769	-1.497385
F	3.022920	-1.480320	-1.283000

E(e)	-1604.1982237
ZPE	0.207270
E(298)	-1603.969245
H(298)	-1603.968301
G(298)	-1603.050675

Frequencies:

Stretch (sym)	131.90
Stretch (asym)	218.59
Twist	34.31

[(Py-Ag-Py)OTs] (6-Ag); DFT; CD_2Cl_2

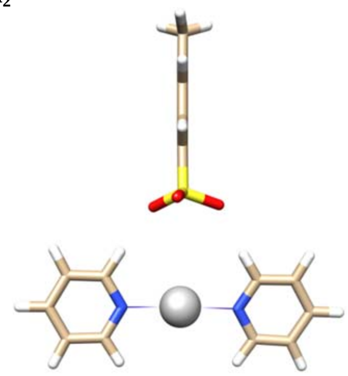

Charge = 0 Multiplicity = 0

C	6.354297	-0.049476	-0.470955

C	5.368110	-0.101424	-1.467035
C	4.017409	-0.089572	-1.142521
C	3.629189	-0.023943	0.197369
C	4.589544	0.029897	1.201453
C	5.943220	0.017202	0.862116
C	7.817688	-0.071854	-0.840072
H	5.663616	-0.149301	-2.511172
H	3.267996	-0.125088	-1.925268
H	4.278587	0.086961	2.237388
H	6.688266	0.062552	1.650682
H	8.054477	0.709953	-1.567659
H	8.093912	-1.028857	-1.294455
H	8.451757	0.077574	0.035719
S	1.867173	-0.028926	0.613498
O	1.366636	-1.391354	0.256400
O	1.249416	1.035795	-0.234054
O	1.782869	0.259262	2.070495
C	-1.315808	4.279468	-0.173614
C	-1.358323	2.890180	-0.144943
C	-2.507673	4.996098	-0.157775
C	-3.710127	4.295215	-0.113096
C	-3.675341	2.908290	-0.086101
N	-2.522559	2.212889	-0.102207
H	-0.451186	2.293709	-0.152931
H	-0.355560	4.780864	-0.206262
H	-2.501753	6.080739	-0.178902
H	-4.664386	4.808554	-0.098271
H	-4.591019	2.329015	-0.050096
Ag	-2.515780	0.025549	-0.060614
N	-2.607014	-2.162853	-0.092869
C	-1.483519	-2.898237	0.023873
C	-1.508416	-4.288162	0.004328
C	-3.785048	-2.799961	-0.233755
C	-3.886371	-4.183528	-0.262033
C	-2.726282	-4.944051	-0.140339
H	-0.550918	-2.353141	0.133570
H	-0.579157	-4.837133	0.102600
H	-2.772620	-6.027778	-0.158147
H	-4.666190	-2.175068	-0.325256
H	-4.858536	-4.648262	-0.377265

E(e)	-1537.5011958
ZPE	0.310821
E(298)	-1537.165849
H(298)	-1537.164905
G(298)	-1537.253765

Frequencies:

Stretch (sym)	136.84
Stretch (asym)	227.39
Twist	45.22

[(Py-Ag-Py)CF_3COO] (7-Ag); DFT; CH_2Cl_2

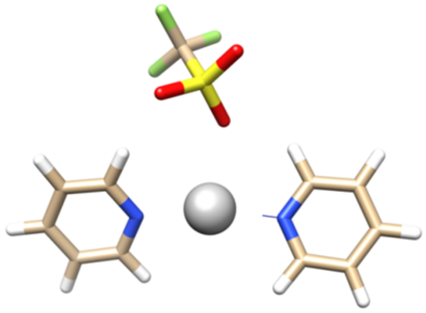

C	-4.255727	-2.576659	-0.300169
C	-2.872922	-2.444958	-0.305001
C	-5.032033	-1.492662	0.099925
C	-4.393777	-0.315800	0.479613
C	-3.005825	-0.263496	0.445110
N	-2.252187	-1.309295	0.060715
H	-2.235993	-3.267463	-0.610019
H	-4.707538	-3.513171	-0.605693
H	-6.114400	-1.564004	0.114554
H	-4.955651	0.554758	0.797307
H	-2.466608	0.633343	0.728316
Ag	-0.030860	-1.062100	0.038664
N	2.174936	-1.414508	0.057922
C	2.979990	-0.388332	0.387424
C	4.364408	-0.504101	0.411937
C	2.738243	-2.594096	-0.258593
C	4.113381	-2.790048	-0.259172
C	4.943303	-1.725838	0.082234
H	2.484790	0.543827	0.634501
H	4.969569	0.352882	0.683760
H	6.021313	-1.846949	0.090528
H	2.060557	-3.399117	-0.519216
H	4.518038	-3.760028	-0.523623
C	0.123379	3.693227	-0.036404
F	-0.854453	4.064784	0.829662
F	0.035891	4.524061	-1.095168
F	1.306450	3.967073	0.573872
C	0.014557	2.172166	-0.413960
O	0.064739	1.421283	0.589278
O	-0.099207	1.907663	-1.615656
E(e)		-1168.9300326	
ZPE		0.206093	
E(298)		-1168.703398	
H(298)	=	-1168.702454	
G(298)		-1168.782246	

Frequencies:

Stretch (sym)	123.19
Stretch (asym)	196.45
Twist	19.63

[(Py-Ag-Py)NO_3] (8-Ag), DFT, CH_2Cl_2

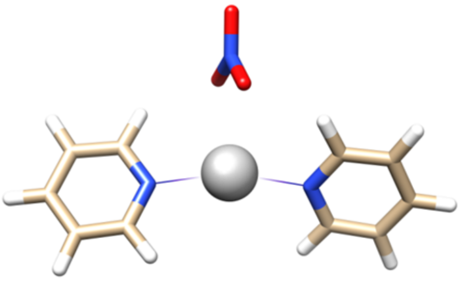

Charge = 0 Multiplicity = 0

N	0.000462	2.824494	0.000004
O	0.378922	2.183473	1.020824
O	0.000769	4.070661	-0.000300
O	-0.378322	2.183176	-1.020536
C	4.420144	0.146281	0.472570
C	3.036180	0.273875	0.466909
C	4.991813	-1.011760	-0.045438
C	4.155223	-2.002838	-0.551415
C	2.781399	-1.799849	-0.519659
N	2.224941	-0.682276	-0.019735
H	2.545959	1.159031	0.855917
H	5.030532	0.945780	0.876048
H	6.068987	-1.139991	-0.055748
H	4.553695	-2.921454	-0.966053
H	2.098961	-2.549365	-0.904637
Ag	-0.000075	-0.340227	0.000068
N	-2.225172	-0.682058	0.019904
C	-3.036213	0.274191	-0.466870
C	-4.420193	0.146759	-0.472782
C	-2.781849	-1.799563	0.519725
C	-4.155703	-2.002392	0.551242
C	-4.992090	-1.011218	0.045121
H	-2.545825	1.159296	-0.855774
H	-5.030410	0.946331	-0.876373
H	-6.069281	-1.139326	0.055236
H	-2.099568	-2.549165	0.904815
H	-4.554345	-2.920966	0.965813

E(e)	-922.9494194
ZPE	0.194279
E(298)	-922.737532
H(298)	-922.736588
G(298)	-922.808679

Frequencies:

Stretch (sym)	126.09
Stretch (asym)	199.77
Twist	23.66

4.2 Thermochemistry

In the tables below, absolute energies are given in Hartree units, relative energies in kJ/mol.

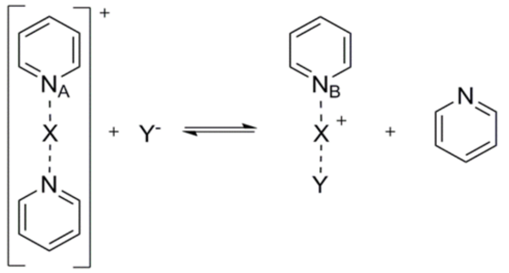

(1)

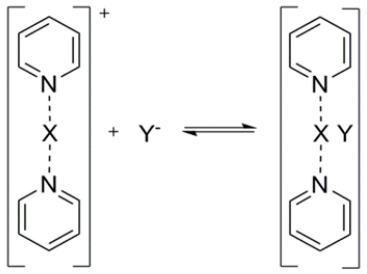

(2)

TABLE S22. The estimated energies for the (1) hypothetical reaction of a pyridine-counterion exchange, and for (2) formation of the [bis(pyridine)iodine] – counterion ion pair.

	H (Ha)	*G* (Ha)
Py	-248.26136	-248.29333
$[PyI]^+$	-259.44036	-259.47839
$[PyIPy]^+$	-507.74070	-507.79398

	X		PyI-X		PyIPy-X		**Reaction**			
							(1)		(2)	
	H	*G*	*H*	*G*	*H*	*G*	Δ*H*	Δ***G***	Δ*H*	Δ***G***
BF_4^-	-424.74371	-424.77444	-684.20614	-684.26480	-932.49389	-932.56773	44.3	**28.9**	-25.0	**5.4**
ClO_4^-	-760.99125	-761.02171	-1020.45963	-1020.51605	-1268.74191	-1268.81468	28.7	**18.5**	-26.2	**6.2**
PF_6^-	-940.94616	-940.98084	-1200.40307	-1200.46536	-1448.69544	-1448.77213	58.8	**44.2**	-22.6	**10.7**
SbF_6^-	-604.98665	-605.02713	-864.44432	-864.51339	-1112.73712	-1112.82099	56.8	**39.7**	-25.8	**3.9**
Otf^-	-961.76051	-961.80138	-1221.23372	-1221.29796	-1469.51282	-1469.59314	16.0	**12.6**	-30.6	**9.4**
OTs^-	-894.95687	-895.00644	-1154.44083	-1154.51013	-1402.70043	-1402.78743	-12.2	**-6.1**	-7.6	**37.7**
NO_3^-	-280.52758	-280.55549	-540.00966	-540.06310	-788.27920	-788.34896	-7.3	**-16.4**	-28.8	**4.9**
CF_3COO^-	-526.49203	-526.52988	-785.97990	-786.04102	-1034.23304	-1034.30983	-22.5	**-25.7**	-0.9	**40.4**

4.3 Isotropic NMR chemical shieldings

$[Py-I-Py]^+$

E(e) = -7416.14147388

I	1	818.07880
N	2	-28.36140
C	3	21.07560
C	4	42.65760
C	5	26.56170
C	6	42.65760
C	7	21.07560
N	8	-28.36140
C	9	21.07560
C	10	42.65760
C	11	26.56170
C	12	42.65760
C	13	21.07560
H	14	22.99030
H	15	23.78260
H	16	23.25610
H	17	23.78260
H	18	22.99030
H	19	22.99030
H	20	23.78260
H	21	23.25610
H	22	23.78260
H	23	22.99030

$[(Py-I)BF_4]$

E(e) = -7592.56580945

C	1	41.24830
C	2	16.48850
C	3	24.45250
C	4	41.22050
C	5	16.34400
N	6	18.77370
H	7	22.99300
H	8	23.73460
H	9	23.18450
H	10	23.76130
H	11	22.96450
I	12	-157.68070
B	13	98.24960
F	14	345.56670
F	15	343.97980
F	16	342.59080
F	17	345.30830

[(Py-I)ClO_4]

E(e) = -7928.86392029

C	1	42.04080
C	2	18.50740
C	3	25.69530
C	4	41.94010
C	5	18.75050
N	6	-2.31430
H	7	23.02270
H	8	23.79380
H	9	23.25500
H	10	23.79600
H	11	22.95250
I	12	171.88770
Cl	13	-151.04110
O	14	-156.05490
O	15	-121.20850
O	16	-121.07530
O	17	-121.07530

[(Py-I)PF_6]

E(e) = -8108.82677323

C	1	40.67300
C	2	15.31110
C	3	23.75030
C	4	40.59330
C	5	15.24220
N	6	29.78780
H	7	22.99250
H	8	23.70380
H	9	23.13760
H	10	23.71690
H	11	22.93930
I	12	-356.21290
P	13	416.77920
F	14	233.71660
F	15	245.96630
F	16	234.54190
F	17	245.34410
F	18	234.54190
F	19	233.39780

[(Py-I)SbF_6]

E(e) = -7772.85525599

C	1	40.53830
C	2	15.31500
C	3	23.69410
C	4	40.59960

C	5	15.35060
N	6	30.02810
H	7	22.95370
H	8	23.71140
H	9	23.14480
H	10	23.71800
H	11	23.00040
I	12	-343.28130
F	13	308.16060
Sb	14	8.84380
F	15	285.89240
F	16	280.26370
F	17	278.35420
F	18	277.64730
F	19	279.03310

[(Py-I)OTf]

E(e) = -8129.67651251

C	1	42.27010
C	2	18.71220
C	3	25.97890
C	4	42.15020
C	5	18.82670
N	6	-5.17340
H	7	23.02780
H	8	23.83530
H	9	23.28880
H	10	23.82270
H	11	22.97970
I	12	237.38950
S	13	138.49750
O	14	94.67210
O	15	89.02500
O	16	111.04560
C	17	43.69220
F	18	252.38180
F	19	251.52010
F	20	250.57180

[(Py-I)OTs]

E(e) = -8062.96648315

S	1	121.23470
O	2	89.70170
C	3	25.76790
O	4	91.98200
O	5	91.98200
C	6	44.05770
C	7	44.05770
C	8	41.45200
C	9	41.45200

C	10	24.04530
C	11	156.83720
I	12	600.06350
N	13	-23.52120
C	14	19.99950
C	15	19.99950
C	16	43.05660
C	17	43.05660
C	18	27.16860
H	19	23.71180
H	20	23.71180
H	21	24.15400
H	22	24.15400
H	23	29.06310
H	24	29.35320
H	25	29.35320
H	26	22.99310
H	27	22.99310
H	28	23.86810
H	29	23.86810
H	30	23.33750

[(Py-I)NO_3]

E(e) = -7448.33664628

C	1	42.93290
C	2	19.73140
C	3	27.03610
C	4	42.89310
C	5	20.11470
N	6	-21.69260
H	7	22.99560
H	8	23.85920
H	9	23.34200
H	10	23.83590
H	11	22.93520
I	12	381.33070
N	13	-152.03860
O	14	-111.17710
O	15	-187.37280
O	16	-193.07170

[(Py-I)CF_3COO]

E(e) = -7694.35956019

I	1	672.61160
N	2	-27.73550
C	3	20.57170
C	4	43.22660
C	5	20.38080
C	6	43.18740

C	7	27.46090
H	8	23.01120
H	9	23.87080
H	10	23.36510
H	11	22.97970
H	12	23.87960
C	13	48.48940
F	14	244.63490
F	15	249.26360
F	16	244.04770
C	17	9.10590
O	18	78.50830
O	19	-58.78800

[(Py-I-Py)BF_4] (1-I)

E(e) = -7840.98186182

C	1	43.92190
C	2	21.85160
C	3	27.59990
C	4	42.84240
C	5	18.26020
N	6	-30.13890
H	7	23.05190
H	8	23.88970
H	9	23.35010
H	10	23.79410
H	11	21.90500
I	12	861.43770
N	13	-30.16650
C	14	18.24540
C	15	42.83570
C	16	21.84510
C	17	43.92180
C	18	27.60040
H	19	21.90220
H	20	23.79600
H	21	23.34890
H	22	23.05310
H	23	23.88670
B	24	97.67760
F	25	343.85920
F	26	335.64380
F	27	335.78080
F	28	344.60440

[(Py-I-Py)ClO_4] (1-I)

E(e) = -8177.27415758

C	1	43.88700
C	2	21.84280
C	3	27.56460

C	4	42.90010
C	5	18.31020
N	6	-29.92610
H	7	23.04850
H	8	23.88230
H	9	23.35300
H	10	23.82650
H	11	21.97190
I	12	878.56750
N	13	-29.94680
C	14	18.31610
C	15	42.89930
C	16	21.86750
C	17	43.87880
C	18	27.56640
H	19	21.95920
H	20	23.81680
H	21	23.35350
H	22	23.04530
H	23	23.88880
Cl	24	-156.99840
O	25	-126.50320
O	26	-125.67120
O	27	-129.73750
O	28	-129.30550

[(Py-I-Py)PF_6] (3-I)

E(e) = -8357.24696177

C	1	43.57210
C	2	21.71430
C	3	27.37690
C	4	42.73660
C	5	19.44230
N	6	-29.57500
H	7	23.02260
H	8	23.88360
H	9	23.35040
H	10	23.78410
H	11	22.42640
I	12	844.97270
N	13	-29.86360
C	14	19.47640
C	15	42.76510
C	16	21.75800
C	17	43.59440
C	18	27.36100
H	19	22.49600
H	20	23.78140
H	21	23.34290
H	22	23.01170
H	23	23.87010
P	24	417.16500

F	25	238.21350
F	26	237.01590
F	27	230.86020
F	28	237.01610
F	29	238.48860
F	30	232.10180

[(Py-I-Py)SbF$_6$] (4-I)

E(e) = -8021.27662948

C	1	43.64930
C	2	21.65520
C	3	27.37560
C	4	42.68000
C	5	19.22920
N	6	-29.28680
H	7	23.01230
H	8	23.87120
H	9	23.32750
H	10	23.78710
H	11	22.36120
I	12	844.31100
N	13	-29.28890
C	14	19.21730
C	15	42.70070
C	16	21.66140
C	17	43.64500
C	18	27.37320
H	19	22.36110
H	20	23.78440
H	21	23.32720
H	22	23.01150
H	23	23.87390
Sb	24	8.37100
F	25	283.63350
F	26	283.93240
F	27	282.45000
F	28	282.70370
F	29	284.03000
F	30	283.06980

[(Py-I-Py)OTf] (4-I)

E(e) = -8378.08457992

C	1	44.04140
C	2	21.93400
C	3	27.66620
C	4	42.90410
C	5	17.64100
N	6	-30.28020
H	7	23.05250

H	8	23.90090
H	9	23.37830
H	10	23.81120
H	11	21.63230
I	12	898.30140
N	13	-30.33640
C	14	17.63570
C	15	42.91110
C	16	21.93200
C	17	43.98930
C	18	27.69420
H	19	21.63890
H	20	23.80200
H	21	23.37100
H	22	23.05420
H	23	23.92010
S	24	138.27130
O	25	88.55210
O	26	84.60980
O	27	84.08670
C	28	42.26460
F	29	254.39600
F	30	253.91750
F	31	254.41620

[(Py-I-Py)OTs] (6-I)

E(e) = -8311.36623542

C	1	44.54340
C	2	21.89860
C	3	28.08830
C	4	43.10870
C	5	15.77420
N	6	-31.19610
H	7	23.05090
H	8	23.93040
H	9	23.40800
H	10	23.88970
H	11	20.76520
I	12	913.50480
N	13	-31.39860
C	14	16.01620
C	15	43.08570
C	16	21.82980
C	17	44.48030
C	18	28.05340
H	19	20.81990
H	20	23.86580
H	21	23.39430
H	22	23.03530
H	23	23.93120
C	24	28.29980
C	25	41.76370

C	26	44.49340
C	27	18.30180
C	28	45.84630
C	29	43.15960
C	30	157.00550
H	31	24.10790
H	32	23.58490
H	33	23.56390
H	34	24.26010
H	35	29.11710
H	36	29.07570
H	37	29.55210
S	38	130.72330
O	39	56.63930
O	40	95.47500
O	41	67.29450

[(Py-I-Py)NO_3] (7-I)

E(e) = -7696.73360899

C	1	42.89770
C	2	18.34280
C	3	27.75310
C	4	44.11850
C	5	21.82020
N	6	-29.00870
H	7	21.69150
H	8	23.75600
H	9	23.35400
H	10	23.90600
H	11	23.03880
I	12	859.48820
N	13	-28.62380
C	14	21.84840
C	15	44.09850
C	16	18.32220
C	17	42.95470
C	18	27.74530
H	19	23.04030
H	20	23.89970
H	21	23.35090
H	22	21.74480
H	23	23.77580
N	24	-166.12380
O	25	-164.51430
O	26	-170.26650
O	27	-163.12390

[(Py-I-Py)CF_3COO] (7-I)

E(e) = -7942.75084097

C	1	44.35570
C	2	22.08050
C	3	27.89640
C	4	42.99290
C	5	16.23420
N	6	-30.69830
H	7	23.07790
H	8	23.92190
H	9	23.38760
H	10	23.78010
H	11	21.07000
I	12	910.37180
N	13	-31.36420
C	14	16.87130
C	15	43.02210
C	16	21.95660
C	17	44.25650
C	18	27.91000
H	19	21.25260
H	20	23.76630
H	21	23.36740
H	22	23.06690
H	23	23.92990
C	24	47.28520
F	25	250.58680
F	26	252.17900
F	27	246.69610
C	28	8.97170
O	29	-0.12460
O	30	-6.16740

4.4 Estimation of the electrolytic stabilization energy

An exact determination of the electrostatic stabilization energy for the electrolytes consisting of $[Py\text{-}I\text{-}Py]^+$ and the counterions Y^- would require extended simulations and is beyond the scope of the present work. We estimated the stabilization energy within the framework of Debye-Hückel theory. The electrostatic stabilization energy per ion pair for monovalent cations and anions amounts to (in Hartree units)

$$\Delta E = -\frac{1}{\varepsilon_r(\lambda_D + R)}$$

where ε_r denotes the relative dielectricity constant of the solvent, R is a characteristic size of the solute molecules, and λ_D is the Debye length

$$\lambda_D = \sqrt{\frac{\varepsilon_r k_B T}{8\pi\varrho}}$$

where ϱ is the (number) density per ion species.

In the experiments, 20 mg of **1-I** to **9-I** each were dissolved in 0.6 ml CD_2Cl_2, corresponding to a mass density of 33.3 g dm^{-3} of the complexes. The molecular masses of the complexes vary between 372 Da (**1-I**) and 520 Da (**4-I**). The compound with the lowest molar concentration (and thus, the largest λ_D and the smallest Debye-Hückel stabilization energy) is thus **4-I**. For this compound, $c = 6.42 \cdot 10^{-2}$ mol dm^{-3}, corresponding to $\rho = 5.72 \cdot 10^{-6}$ (particles per cubic bohr radius). For CD_2Cl_2, $\varepsilon_r = 8.93$. Furthermore, $k_BT = 9.443 \cdot 10^{-4}$ Hartree for $T = 298.15$ K. This leads to $\lambda_D = 7.659$ a.u. For the characteristic size of the solute molecules, we choose a value of 6 a.u., reflecting the fact that the centers of the opposite charges may approach each other quite closely. The corresponding stabilization energy becomes then $\Delta E = 8.19 \cdot 10^{-2}$ Hartree = 21.5 kJ mol^{-1}. Given that the linearization in standard Debye-Hückel theory tends to underestimate the electrostatic interactions in electrolytes and that we have used the case with the lowest ρ for our estimation, a reasonable estimate for ΔE is 20 - 30 kJ mol^{-1}, as used in the main text of the manuscript.

5. CRYSTAL STRUCTURE DETERMINATIONS

Single crystals of **2-I** - **8-I**, and **2-Ag** - **7-Ag** were obtained by slow diffusion of hexane into a dichloroethane solution (2.0 mL) of the complex (50-150 mg). Thereafter, the vial was cooled gradually from 5 °C to -8 °C over 24 hours. The crystals were kept at -20 °C ahead of their X-ray diffractometric study.

The X-ray data for **4-I**, **5-I**, **6-I**, **7-I**, **4-Ag**, **6-Ag** and **7-Ag_2** were collected on an *Agilent SuperNova Dual* diffractometer with *Atlas* detector using mirror-monochromatized Cu-Kα (λ = 1.54184 Å, only in case of **5-I** and **12-Ag**) or Mo-Kα radiation (λ = 0.71073 Å). The X-ray data for **2-I**, **3-I**, **1-Ag**, **2-Ag** and **7-Ag** were collected on an *Agilent SuperNova* diffractometer with *Eos* detector using mirror-monochromatized Mo-Kα (λ = 0.71073 Å) radiation, while those for **3-Ag** and **5-Ag** were collected on a *Bruker-Nonius KappaCCD* diffractometer with an *APEX-II* detector using graphite-monochromatized Mo-Kα (λ = 0.71073 Å) radiation.
The data collection were performed at T = 123.0(1) K for **5-I**, **6-I** and **12-Ag**, T = 170.0(1) K for **2-I**, **3-I**, **1-Ag**, **2-Ag** and **7-Ag** or T = 173.0(1) K for **4-I**, **7-I**, **3-Ag**, **4-Ag**, **5-Ag**, **6-Ag** and **7-Ag_2**.
CrysAlisPro[1] software was used for data collection, integration and reduction as well as applying the semi-empirical absorption correction for **2-I**, **3-I**, **4-I**, **6-I**, **1-Ag**, **2-Ag**, **4-Ag**, **6-Ag**, **7-Ag** and **7-Ag_2**, numerical absorption correction based on gaussian integration in case of **5-I** and **7-I** and analytical numeric absorption correction in case of **12-Ag**. For **3-Ag** and **5-Ag**, the data collection and reduction were performed using the program *COLLECT*[2] and *HKL DENZO AND SCALEPACK*,[3] respectively, and the intensities were corrected for absorption using *SADABS*.[4]
The structures were solved by direct methods (**2-I**, **5-I**, **3-Ag**, **5-Ag**, **12-Ag**) using *SHELXS-97*,[5] *SIR-2002*[6] (**3-I**, **1-Ag**, **2-Ag**, **7-Ag**) or *SIR-97*[7] (**4-Ag**, **7-Ag_2**, **7-I**) or charge flipping with *Superflip*[8] (**4-I**, **6-I**, **6-Ag**) and refined by full-matrix least-squares using *SHELXL-2013*[5] (or *SHELXL-2014/7* in case of **7-I**) within *WinGX*[9] or *OLEX2*[10] package.
All non-hydrogen atoms were refined anisotropically. All carbon-bound hydrogen atoms were calculated to their optimal positions and treated as riding atoms using isotropic displacement parameters U_H = 1.2 U_C (or U_H = 1.5 U_C in case of methyl groups). Hydrogen atoms of a water molecule in **7-Ag_2** were found from the difference Fourier map and modelled using geometrical restraints and isotropic displacement parameters U_H = 1.5 U_O.
The structure **5-I** is a twin and the two components of the twin crystal were analyzed by *PLATON*[11] with the final refined[9] ratio for the two components at 0.72:0.28. Fluorine atoms F1-F3 of tetrafluoroborate ion in **1-Ag** were disordered and were refined with fixed occupancy ratio of 85/15 %. Restraints on anisotropic thermal parameters and some geometric restraints were applied in the refinement of the anion. In addition, some restraints were applied on anisotropic thermal parameters of C12 atom and geometric restraint on C11−C12 bond distance of dichloroethane molecule. Geometric restraint was also applied in the refinement of C11−C12 bond distance of dichloroethane molecule in **2-Ag**.

5.1 Crystal data:

2-I: 0.18×0.35×0.66 mm, $C_{10}H_{10}ClIN_2O_4$, M = 384.55 gmol^{-1}, monoclinic, space group $P2_1/n$, a = 12.2849(4) Å, b = 16.0628(3) Å, c = 14.7939(4) Å, α = 90°, β = 113.273(3)°, γ = 90°, V = 2681.74(14) Å^3, Z = 8, ρ = 1.905 g cm^{-3}, μ = 2.595 mm^{-1}, F(000) = 1488, 8696 reflections (θ_{max} = 25.499°) measured (4960 unique, R_{int} = 0.0161, completeness = 99.3%), Final R indices ($I > 2\sigma(I)$): R_1= 0.0278, wR_2 = 0.0790, R indices (all data): R_1= 0.0384, wR_2 = 0.0872. GOF = 1.020 for 328 parameters and 0 restraints, largest diff. peak and hole 0.551/–0.715 eÅ^{-3}. CCDC-1045981 contains the supplementary data for this structure.

3-I: 0.11×0.24×0.30 mm, $C_{10}H_{10}F_6IN_2P$, M = 430.07 gmol^{-1}, monoclinic, space group $C2/m$, a = 13.6827(6) Å, b = 6.7491(3) Å, c = 8.4045(3) Å, α = 90°, β = 106.045(4)°, γ = 90°, V = 745.89(6) Å^3, Z = 2, ρ = 1.915 g cm^{-3}, μ = 2.309 mm^{-1}, F(000) = 412, 1310 reflections (θ_{max} = 25.488°) measured (754 unique, R_{int} = 0.0136, completeness = 98.9%), Final

1 *CrysAlisPro*, 1.171.36.28 ed. and 1.171.37.31 ed., Agilent Technologies, Ltd., Yarton, UK, 2009–2013
2 Bruker AXS BV, Madison, WI, USA; 1997–2004
3 Z. Otwinowski and W. Minor, *Methods Enzymol.* **1997**, *276*, 307
4 Bruker (2001). *SADABS*. Bruker AXS Inc., Madison, Wisconsin, USA
5 G. Sheldrick, *Acta Crystallogr.* **2008**, *A64*, 112–122
6 M. C. Burla, M. Camalli, B. Carrozzini, G. L. Cascarano, C. Giacovazzo, G. Polidori, & R. Spagna, *J. Appl. Cryst.* **2003**, *36*, 1103.
7 A. Altomare, M. C. Burla, M. Camalli, G. L. Cascarano, C. Giacovazzo, A. Guagliardi, A. G. G. Moliterni, G. Polidori, & R. Spagna, *J. Appl. Cryst.* **1999**, *32*, 115.
8 L. Palatinus, G. Chapuis, *J. Appl. Cryst.* **2007**, *40*, 786.
9 L. J. Farrugia, *J. Appl. Cryst.* **2012**, *45*, 849.
10 O. V. Dolomanov, L. J. Bourhis, R. J. Gildea, J. A. K. Howard and H. Puschmann, *J. Appl. Cryst.* **2009**, *42*, 339–341
11 A. L. Spek, *Acta Crystallogr.* **2009**, *D65*, 148.

R indices ($I > 2\sigma(I)$): R_1= 0.0172, wR_2 = 0.0427, R indices (all data): R_1= 0.0172, wR_2 = 0.0427. GOF = 1.020 for 60 parameters and 0 restraints, largest diff. peak and hole 0.282/–0.373 eÅ^{-3}. CCDC-1045982 contains the supplementary data for this structure.

4-I: 0.094×0.262×0.295 mm, $C_{10}H_{10}F_6IN_2Sb$, M = 520.85 gmol^{-1}, triclinic, space group $P\bar{1}$, a = 6.9674(4) Å, b = 7.1797(3) Å, c = 7.7358(4) Å, α = 91.775(4)°, β = 101.402(5)°, γ = 96.682(4)°, V = 376.18(3) Å^3, Z = 1, ρ = 2.299 g cm^{-3}, μ = 3.939 mm^{-1}, F(000) = 242, 2255 reflections (θ_{max} = 25.244°) measured (1357 unique, R_{int} = 0.0205, completeness = 98.9%), Final R indices ($I > 2\sigma(I)$): R_1= 0.0218, wR_2 = 0.0562, R indices (all data): R_1= 0.0268, wR_2 = 0.0638. GOF = 1.093 for 94 parameters and 0 restraints, largest diff. peak and hole 0.913/–0.613 eÅ^{-3}. CCDC- 1045983 contains the supplementary data for this structure.

5-I: 0.061×0.099×0.1843 mm, $C_{11}H_{10}F_3N_2O_3SI$, M = 434.17 gmol^{-1}, monoclinic, space group $P\bar{1}$, a = 7.0091(4)Å, b = 10.6722(6)Å, c = 10.7542(8)Å, α = 93.455 (5)°, β = 105.677(6)°, γ = 104.138(5)°, V = 744.17(9)Å^3, Z = 2, ρ = 1.938 g cm^{-3}, μ = 18.656 mm^{-1}, F(000) = 420, 4365 reflections (θ_{max} = 66.75°) measured (2630 unique, R_{int} = 0.0481, completeness = 99.2%), Final R indices ($I > 2\sigma(I)$): R_1= 0.0502, wR_2 = 0.1616, R indices (all data): R_1= 0.0526, wR_2 = 0.1635. GOF = 1.144 for 191 parameters and 0 restraints, largest diff. peak and hole 1.578/–0.995 eÅ^{-3}. CCDC-1045984 contains the supplementary data for this structure.

6-I: 0.056×0.131×0.292 mm, $C_{17}H_{17}IN_2O_3S$, M = 456.28 gmol^{-1}, monoclinic, space group $P2_1/n$, a = 9.0892(4) Å, b = 21.1577(13) Å, c = 10.0924(5) Å, α = 90°, β = 115.781(6)°, γ = 90°, V = 1747.65(18) Å^3, Z = 4, ρ = 1.734 g cm^{-3}, μ = 1.969 mm^{-1}, F(000) = 904, 6586 reflections (θ_{max} = 25.244°) measured (3142 unique, R_{int} = 0.0271, completeness = 99.5%), Final R indices ($I > 2\sigma(I)$): R_1= 0.0297, wR_2 = 0.0565, R indices (all data): R_1= 0.0360, wR_2 = 0.0588. GOF = 1.084 for 218 parameters and 0 restraints, largest diff. peak and hole 0.617/–0.676 eÅ^{-3}. CCDC-1045985 contains the supplementary data for this structure.

7-I: 0.152×0.232×0.288 mm, $C_{10}H_{10}IN_3O_3$, M = 347.11 gmol^{-1}, triclinic, space group $P\bar{1}$, a = 7.0864(3) Å, b = 7.4613(3) Å, c = 13.5366(6) Å, α = 99.491(4)°, β = 94.725(4)°, γ = 117.637(4)°, V = 614.97(5) Å^3, Z = 2, ρ = 1.875 g cm^{-3}, μ = 2.609 mm^{-1}, F(000) = 336, 7724 reflections (θ_{max} = 29.410°) measured (5116 unique, R_{int} = 0.020, completeness = 99.9%), Final R indices ($I > 2\sigma(I)$): R_1= 0.0332, wR_2 = 0.0956, R indices (all data): R_1= 0.0527, wR_2 = 0.1043. GOF = 1.000 for 186 parameters and 142 restraints, largest diff. peak and hole 0.789/–0.796 eÅ^{-3}. CCDC-1045986 contains the supplementary data for this structure.

1-Ag: 0.07×0.16×0.43 mm, $C_{12}H_{14}AgBCl_2F_4N_2$, M = 451.83 gmol^{-1}, monoclinic, space group $P2_1/n$, a = 7.2894(2) Å, b = 13.3581(3) Å, c = 16.9874(5) Å, α = 90°, β = 101.989(3)°, γ = 90°, V = 1618.03(8) Å^3, Z = 4, ρ = 1.855 g cm^{-3}, μ = 1.610 mm^{-1}, F(000) = 888, 5386 reflections (θ_{max} = 25.5°) measured (2984 unique, R_{int} = 0.0167, completeness = 99.2%), Final R indices ($I > 2\sigma(I)$): R_1= 0.0308, wR_2 = 0.0795, R indices (all data): R_1= 0.0363, wR_2 = 0.0834. GOF = 1.186 for 226 parameters and 28 restraints, largest diff. peak and hole 0.673/–0.630 eÅ^{-3}. CCDC-1045987 contains the supplementary data for this structure.

2-Ag: 0.08×0.11×0.62 mm, $C_{12}H_{14}AgCl_3N_2O_4$, M = 464.47 gmol^{-1}, monoclinic, space group $P2_1/n$, a = 7.2442(2) Å, b = 13.3604(3) Å, c = 16.9657(4) Å, α = 90°, β = 101.394(3)°, γ = 90°, V = 1609.67(7) Å^3, Z = 4, ρ = 1.917 g cm^{-3}, μ = 1.767 mm^{-1}, F(000) = 920, 5258 reflections (θ_{max} = 25.498°) measured (2984 unique, R_{int} = 0.0163, completeness = 99.4%), Final R indices ($I > 2\sigma(I)$): R_1= 0.0294, wR_2 = 0.0737, R indices (all data): R_1= 0.0339, wR_2 = 0.0765. GOF = 1.171 for 199 parameters and 1 restraint, largest diff. peak and hole 0.761/–0.547 eÅ^{-3}. CCDC-1045988 contains the supplementary data for this structure.

3-Ag: 0.04×0.05×0.38 mm, $C_{12}H_{14}AgCl_2F_6N_2P$, M = 509.99 gmol^{-1}, triclinic, space group $P\bar{1}$, a = 9.968(2) Å, b = 10.512(2) Å, c = 10.679(2)Å, α = 111.37(3)°, β = 116.82(3)°, γ = 97.43(3)°, V = 869.9(3)Å^3, Z = 2, ρ = 1.947 g cm^{-3}, μ = 1.614 mm^{-1}, F(000) = 500, 7991 reflections (θ_{max} = 28.51°) measured (4325 unique, R_{int} = 0.0372, completeness = 97.8%), Final R indices ($I > 2\sigma(I)$): R_1= 0.0535, wR_2 = 0.0915, R indices (all data): R_1= 0.0818, wR_2 = 0.1009. GOF = 1.050 for 217 parameters and 0 restraints, largest diff. peak and hole 0.618/–0.592 eÅ^{-3}. CCDC-1045989 contains the supplementary data for this structure.

4-Ag: 0.112×0.185×0.308 mm, $C_{12}H_{14}AgCl_2F_6N_2Sb$, M = 600.77 gmol^{-1}, monoclinic, space group $P2_1/c$, a = 12.6882(3) Å, b = 13.8467(3) Å, c = 11.1581(3) Å, α = 90°, β = 112.120(3)°, γ = 90°, V = 1816.07(8) Å^3, Z = 4, ρ = 2.197 g cm^{-3}, μ = 2.914 mm^{-1}, F(000) = 1144, 6577 reflections (θ_{max} = 25.247°) measured (3267 unique, R_{int} = 0.0179, completeness = 99.3%), Final R indices ($I > 2\sigma(I)$): R_1= 0.0203, wR_2 = 0.0424, R indices (all data): R_1= 0.0234, wR_2 = 0.0445. GOF =

1.046 for 217 parameters and 0 restraints, largest diff. peak and hole 0.322/–0.643 eÅ^{-3}. CCDC-1045990 contains the supplementary data for this structure.

5-Ag: 0.20×0.23×0.31 mm, $C_{11}H_{10}AgF_3N_2O_3S$, M = 415.14 gmol^{-1}, monoclinic, space group $P2_1/c$, a = 7.9520(4)Å, b = 17.2940(6)Å, c = 10.7947(6)Å, α = 90°, β = 95.624(2)°, γ = 90°, V = 1477.36(12)Å^3, Z = 4, ρ = 1.866 g cm^{-3}, μ = 1.548 mm^{-1}, F(000) = 816, 8464 reflections (θ_{max} = 28.525°) measured (3698 unique, R_{int} = 0.0293, completeness = 98.2%), Final R indices ($I > 2\sigma(I)$): R_1= 0.0508, wR_2 = 0.0980, R indices (all data): R_1= 0.0970, wR_2 = 0.1141. GOF = 1.021 for 190 parameters and 0 restraints, largest diff. peak and hole 0.413/–0.390 eÅ^{-3}. CCDC-1045991 contains the supplementary data for this structure.

6-Ag: 0.113×0.238×0.270 mm, $C_{17}H_{17}AgN_2O_3S$, M = 437.25 gmol^{-1}, monoclinic, space group $P2_1/c$, a = 9.8088(2) Å, b = 15.4242(3) Å, c = 11.5973(3) Å, α = 90°, β = 100.033(2)°, γ = 90°, V = 1727.76(7) Å^3, Z = 4, ρ = 1.681 g cm^{-3}, μ = 1.305 mm^{-1}, F(000) = 880, 6508 reflections (θ_{max} = 25.245°) measured (3090 unique, R_{int} = 0.0175, completeness = 99.2%), Final R indices ($I > 2\sigma(I)$): R_1= 0.0214, wR_2 = 0.0488, R indices (all data): R_1= 0.0243, wR_2 = 0.0504. GOF = 1.048 for 218 parameters and 0 restraints, largest diff. peak and hole 0.416/–0.433 eÅ^{-3}. CCDC-1045992 contains the supplementary data for this structure.

7-Ag: 0.06×0.17×0.25 mm, $C_{15}H_{15}Ag_2N_5O_6$, M = 577.06 gmol^{-1}, triclinic, space group $P\bar{1}$, a = 7.4958(3) Å, b = 10.0405(5) Å, c = 12.7988(6) Å, α = 82.991(4)°, β = 83.259(4)°, γ = 76.743(4)°, V = 926.59(8) Å^3, Z = 2, ρ = 2.068 g cm^{-3}, μ = 2.157 mm^{-1}, F(000) = 564, 5220 reflections (θ_{max} = 25.497°) measured (3424 unique, R_{int} = 0.0153, completeness = 99.3%), Final R indices ($I > 2\sigma(I)$): R_1= 0.0280, wR_2 = 0.0607, R indices (all data): R_1= 0.0334, wR_2 = 0.0657. GOF = 1.023 for 253 parameters and 0 restraints, largest diff. peak and hole 0.603/–0.501 eÅ^{-3}. CCDC-1045993 contains the supplementary data for this structure.

7-Ag_2: 0.140×0.159×0.505 mm, $C_{10}H_{12}AgN_3O_4$, M = 346.10 gmol^{-1}, monoclinic, space group $I2/a$, a = 5.7265(2) Å, b = 14.7681(6) Å, c = 14.6482(6) Å, α = 90°, β = 96.590(4)°, γ = 90°, V = 1230.61(8) Å^3, Z = 4, ρ = 1.868 g cm^{-3}, μ = 1.648 mm^{-1}, F(000) = 688, 2263 reflections (θ_{max} = 25.239°) measured (1111 unique, R_{int} = 0.0145, completeness = 99.0%), Final R indices ($I > 2\sigma(I)$): R_1= 0.0179, wR_2 = 0.0404, R indices (all data): R_1= 0.0195, wR_2 = 0.0415. GOF = 1.097 for 88 parameters and 3 restraints, largest diff. peak and hole 0.217/–0.357 eÅ^{-3}. CCDC-1045994 contains the supplementary data for this structure.

12-Ag: 0.170×0.257×0.286 mm, $C_{22.5}H_{17}AgCl_5N_3O_3$, M = 662.51 gmol^{-1}, triclinic, space group $P\bar{1}$, a = 7.2198(3) Å, b = 13.3083(5) Å, c = 14.6862(5) Å, α = 116.693(4)°, β = 90.960(3)°, γ = 90.319(3)°, V = 1260.39(9) Å^3, Z = 2, ρ = 1.746 g cm^{-3}, μ = 11.562 mm^{-1}, F(000) = 658, 9133 reflections (θ_{max} = 66.747°) measured (4436 unique, R_{int} = 0.0357, completeness = 99.1%), Final R indices ($I > 2\sigma(I)$): R_1= 0.0406, wR_2 = 0.1067, R indices (all data): R_1= 0.0419, wR_2 = 0.1081. GOF = 1.065 for 316 parameters and 0 restraints, largest diff. peak and hole 2.263/–1.046 eÅ^{-3}. CCDC-1045995 contains the supplementary data for this structure.

These data can be obtained free of charge via www.ccdc.cam.ac.uk/data_request/cif, or by emailing data_request@ccdc.cam.ac.uk, or by contacting The Cambridge Crystallographic Data Centre, 12, Union Road, Cambridge CB2 1EZ, UK; fax: +44 1223 336033.

6. ^{1}H AND ^{13}C NMR SPECTRA OF THE STUDIED COMPOUNDS

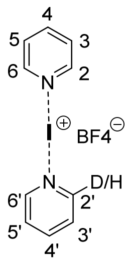

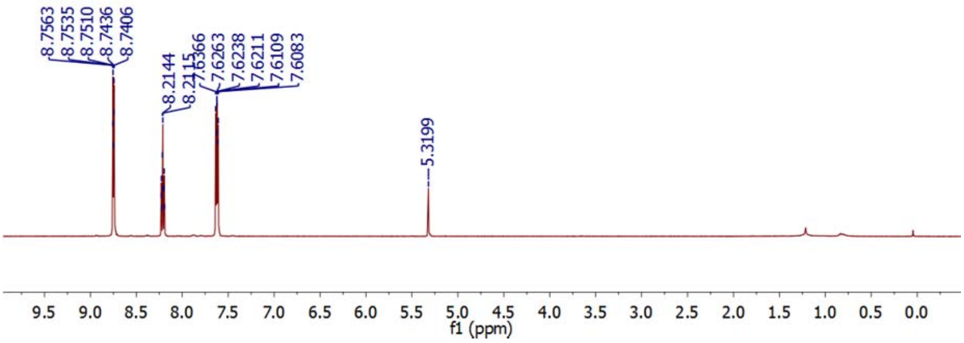

Figure S15. The ^{1}H NMR spectrum of [bis(pyridine)iodine] tetrafluoroborate (**1-I/1-I-*d***) acquired at 20°C in CD_2Cl_2 at 499.89 MHz.

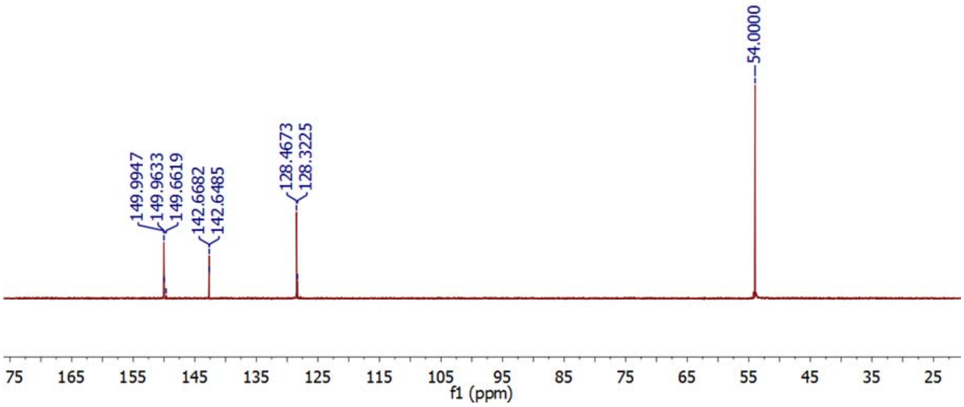

Figure S16. The ^{13}C NMR spectrum of [bis(pyridine)iodine] tetrafluoroborate (**1-I/1-I-*d***) acquired at 20°C in CD_2Cl_2 at 125.61 MHz.

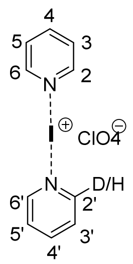

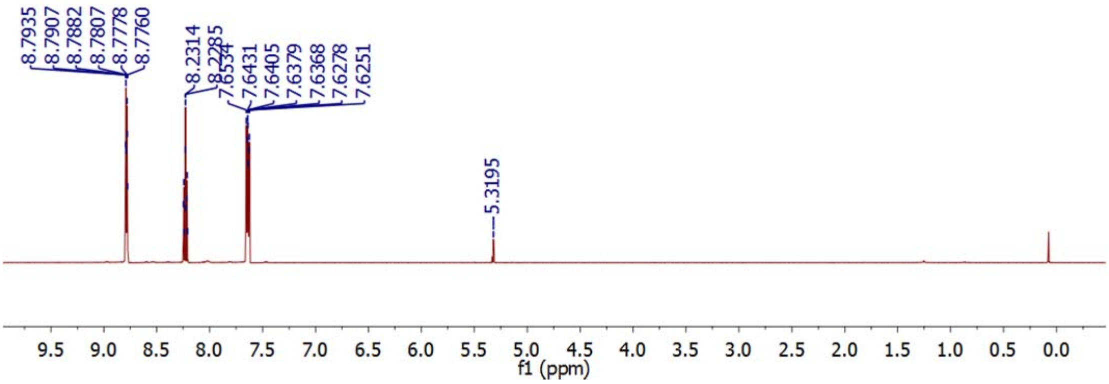

Figure S17. The ^{1}H NMR spectrum of [bis(pyridine)iodine] perchlorate (**2-I/2-I-*d***) acquired at 25°C in CD_2Cl_2 at 499.89 MHz.

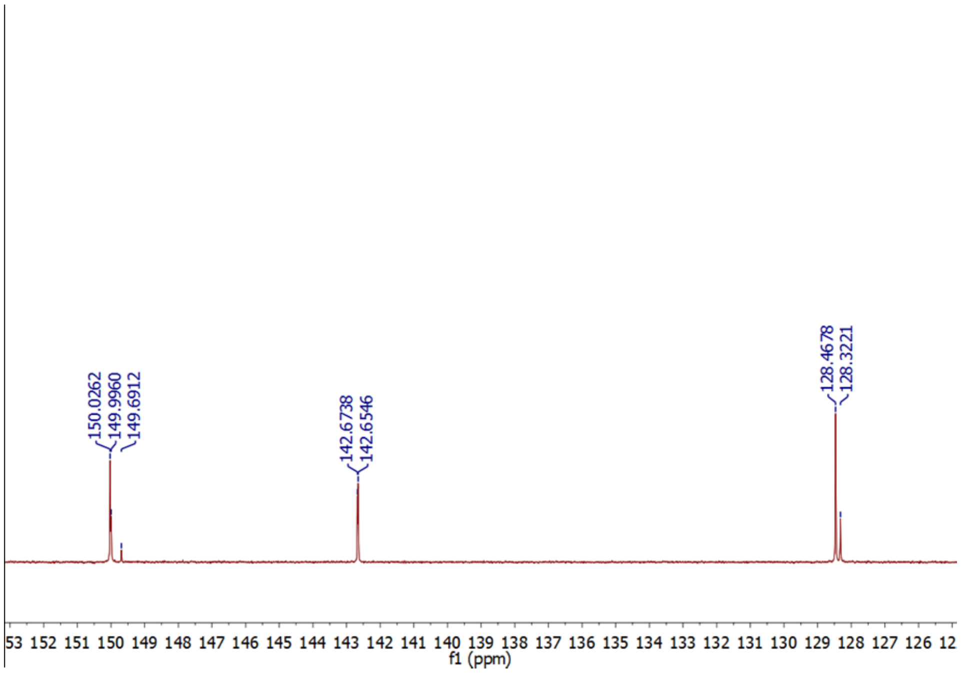

Figure S18. The ^{13}C NMR spectrum of [bis(pyridine)iodine] perchlorate (**2-I/2-I-*d***) acquired at 25 °C in CD_2Cl_2 at 125.61 MHz.

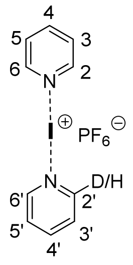

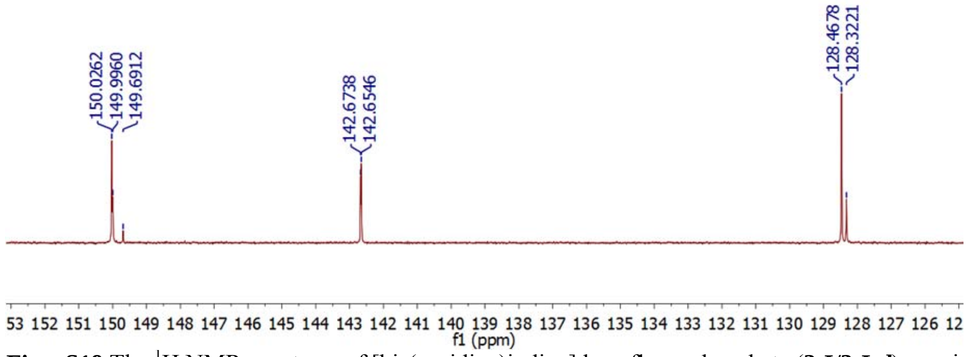

FigurS19.The ^{1}H NMR spectrum of [bis(pyridine)iodine] hexafluorophosphate (**3-I/3-I-*d***) acquired at 25 °C in CD_2Cl_2 at 499.89 MHz

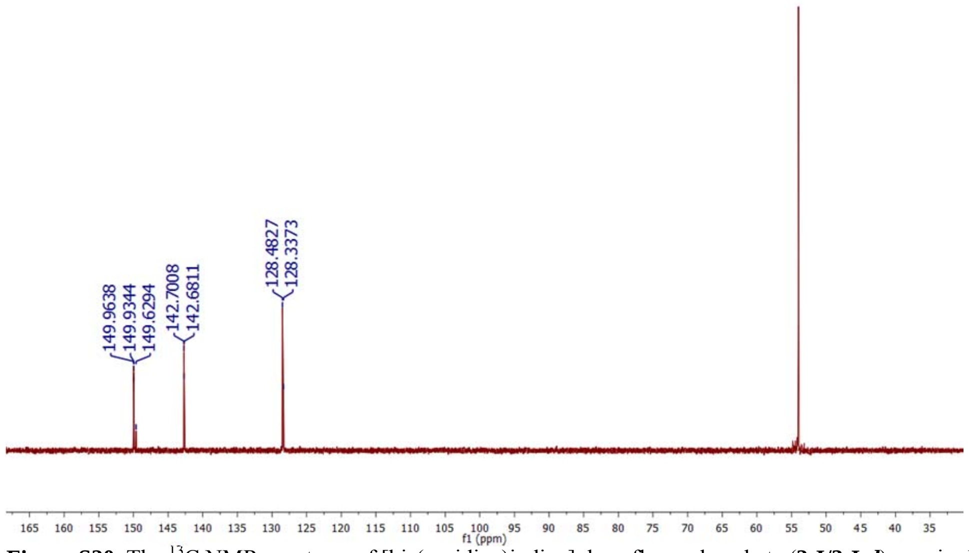

Figure S20. The ^{13}C NMR spectrum of [bis(pyridine)iodine] hexafluorophosphate (**3-I/3-I-*d***) acquired at 25°C in CD_2Cl_2 at 125.61 MHz.

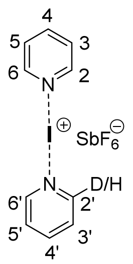

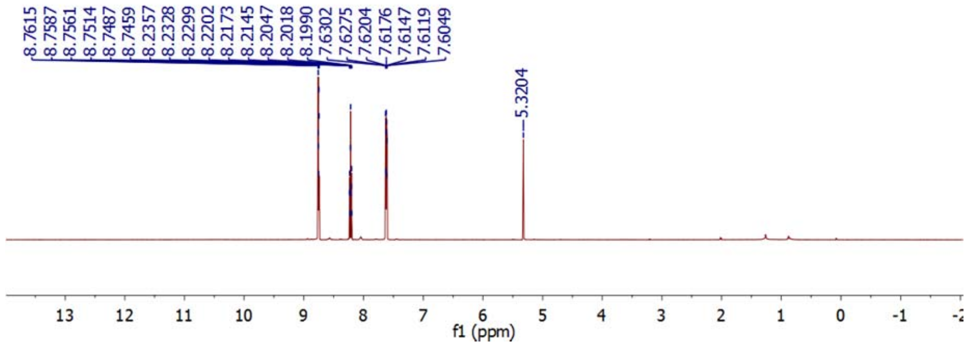

FigureS21.The ^{1}H NMR spectrum of [bis(pyridine)iodine] hexafluoroantimonate (**4-I/4-I-*d***) acquired at 25 °C in CD_2Cl_2 at 499.89 MHz.

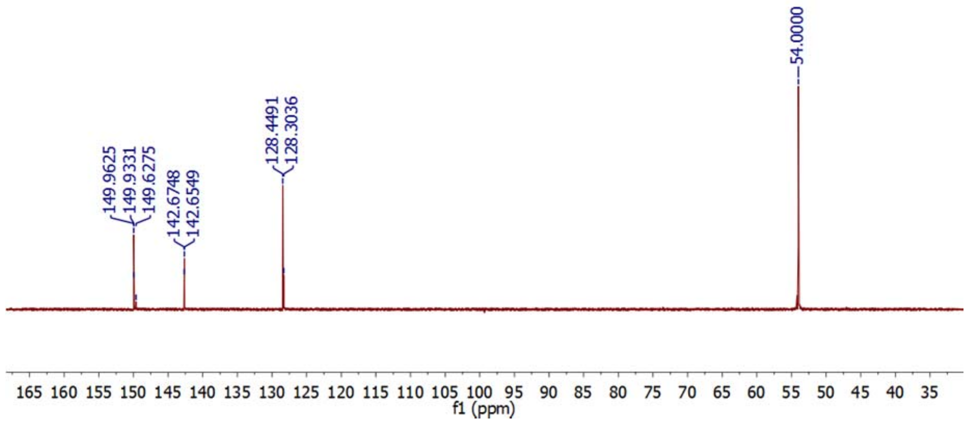

Figure S22. The ^{13}C NMR spectrum of [bis(pyridine)iodine] hexafluoroantimonate (**4-I/4-I-*d***) acquired at 25°C in CD_2Cl_2 at 125.61 MHz.

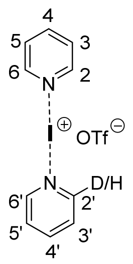

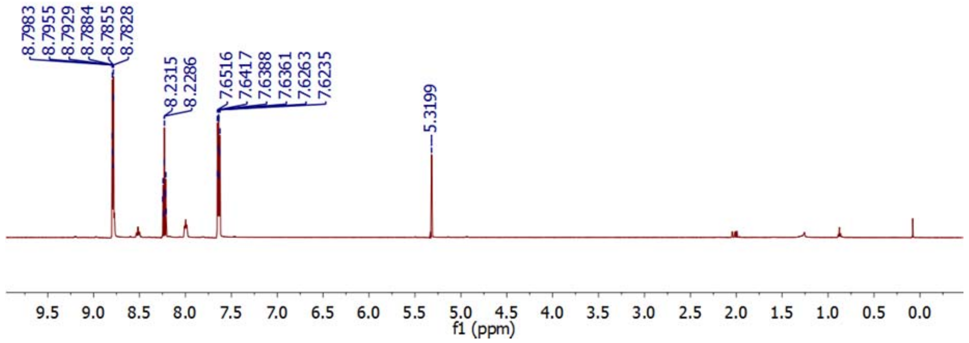

Figure S23. The ^{1}H NMR spectrum of [bis(pyridine)iodine] triflate (**5-I/5-I-*d***) acquired at 25°C in CD_2Cl_2 at 499.89 MHz. The additional peaks in the spectrum originate from decomposition due to humidity

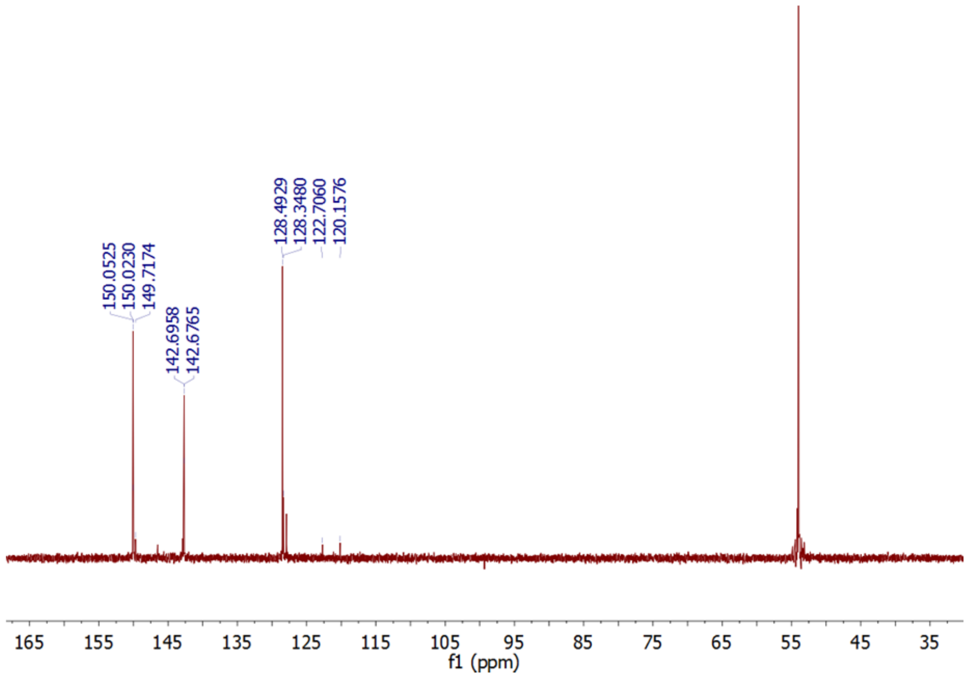

Figure S24. The ^{13}C NMR spectrum of [bis(pyridine)iodine] triflate (**5-I/5-I-*d***) acquired at 25°C in CD_2Cl_2 at 125.61 MHz.

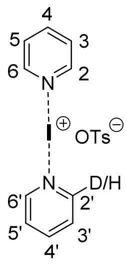

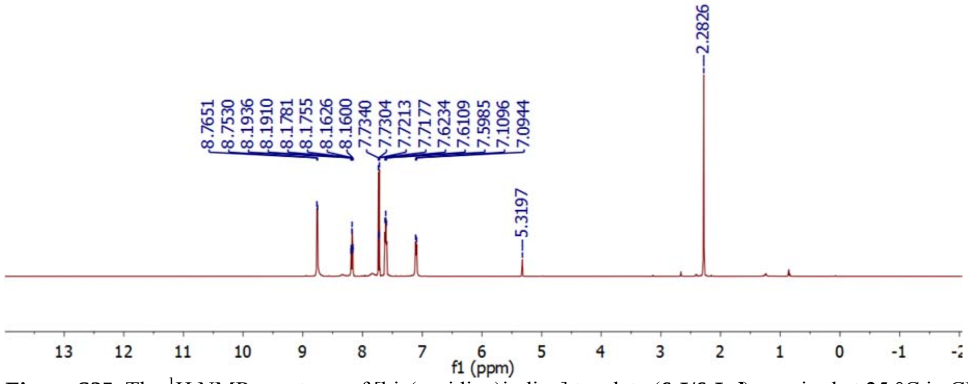

Figure S25. The ^{1}H NMR spectrum of [bis(pyridine)iodine] tosylate (**6-I/6-I-*d***) acquired at 25 °C in CD_2Cl_2 at 499.89 MHz

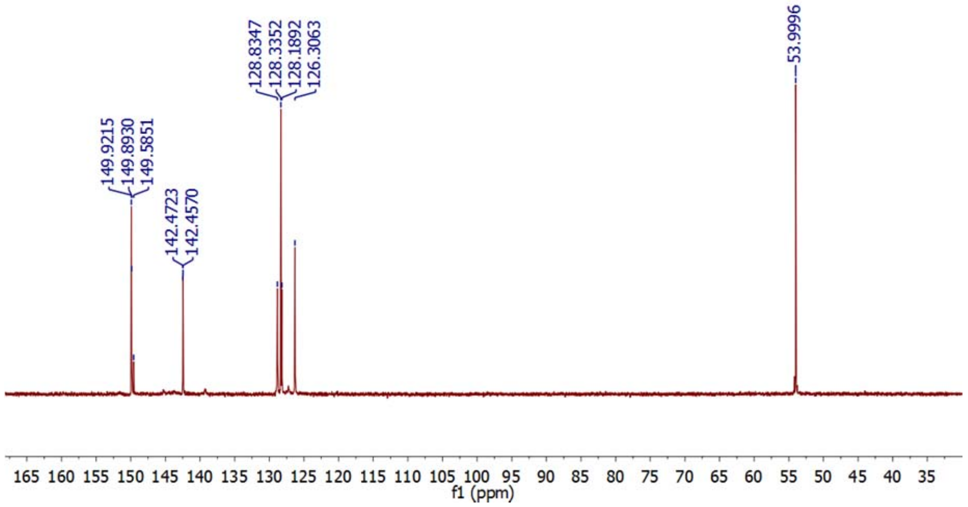

Figure S26. The ^{13}C NMR spectrum of [bis(pyridine)iodine] tosylate (**6-I/6-I-*d***) acquired at 25 °C in CD_2Cl_2 at 125.61 MHz.

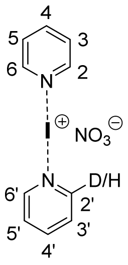

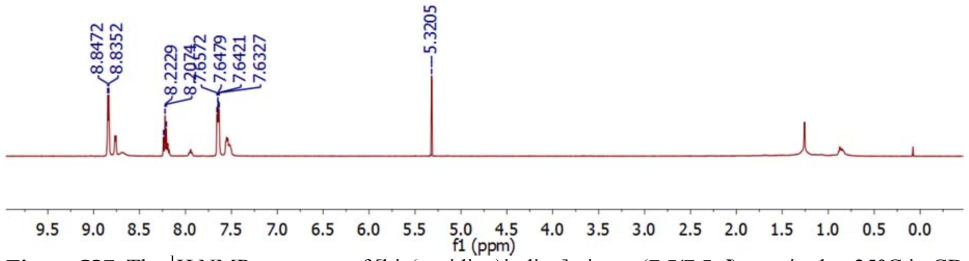

Figure S27. The ^{1}H NMR spectrum of [bis(pyridine)iodine] nitrate (**7-I/7-I-*d***) acquired at 25°C in CD_2Cl_2 at 499.89 MHz. The additional peaks in the spectrum may originate from byproducts due to the redox reaction discussed in *J. Inorg. Nucl. Chem.* **1967**, 29, 407-412.

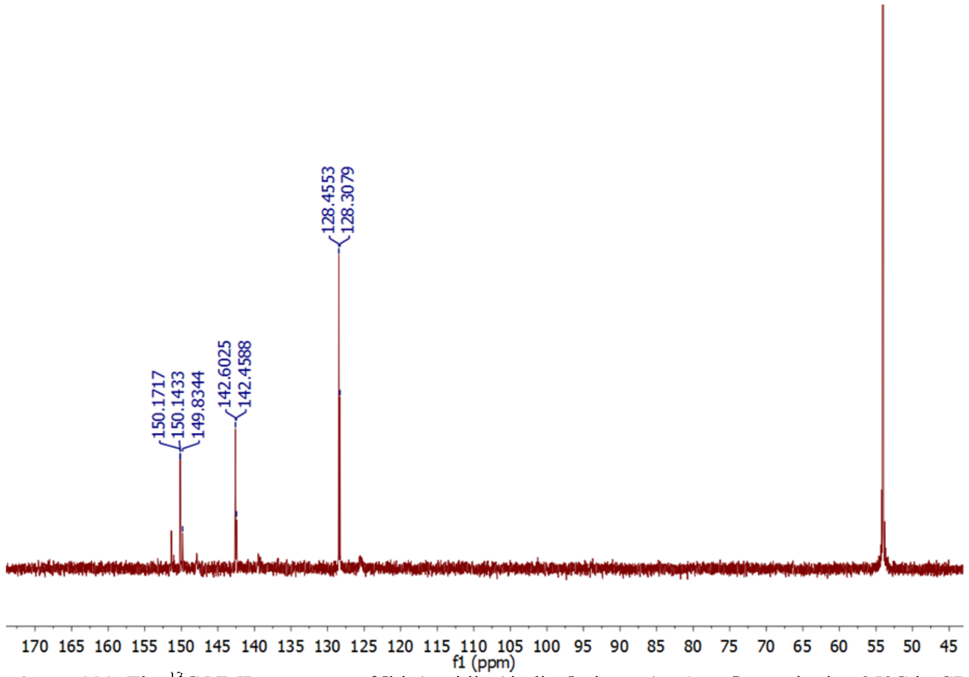

Figure S28. The ^{13}C NMR spectrum of [bis(pyridine)iodine] nitrate (**7-I/7-I-*d***) acquired at 25°C in CD_2Cl_2 at 125.61 MHz.

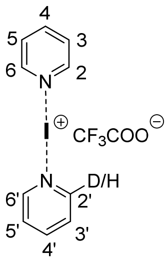

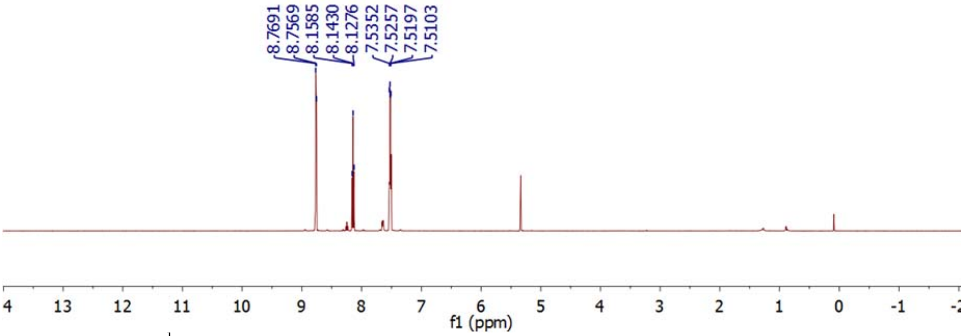

Figure S29. The ^{1}H NMR spectrum of [bis(pyridine)iodine] trifluoroacetate (**8-I/8-I-*d***) acquired at 25°C in CD_2Cl_2 at 499.89 MHz.

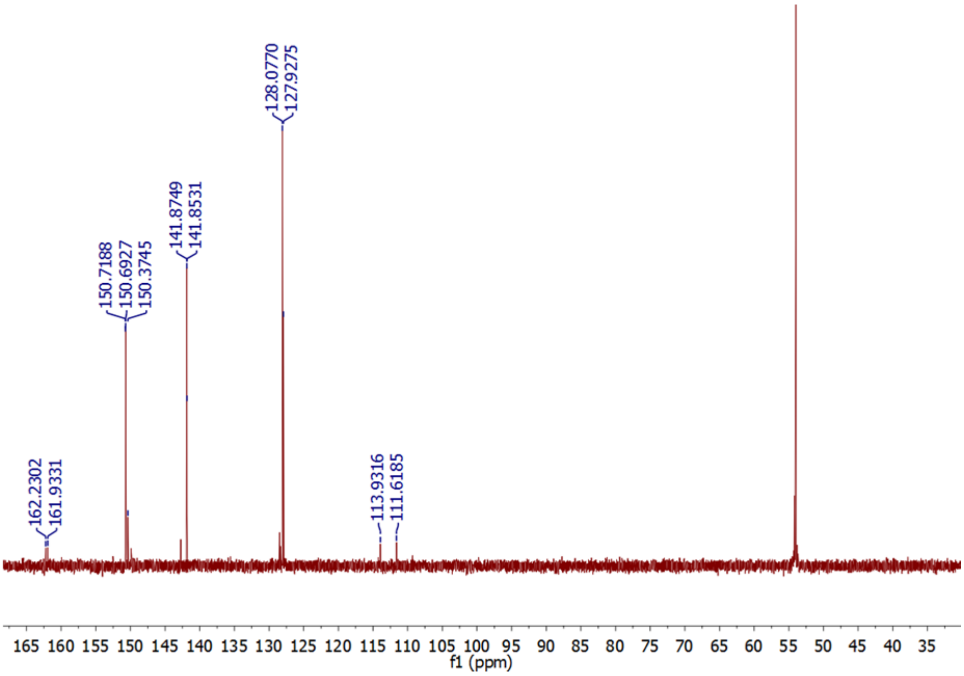

Figure S30. The ^{13}C NMR spectrum of [bis(pyridine)iodine] trifluoroacetate (**8-I/8-I-*d***) acquired at 25°C in CD_2Cl_2at 125.61 MHz

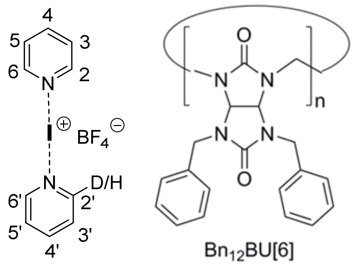

Bn$_{12}$BU[6]

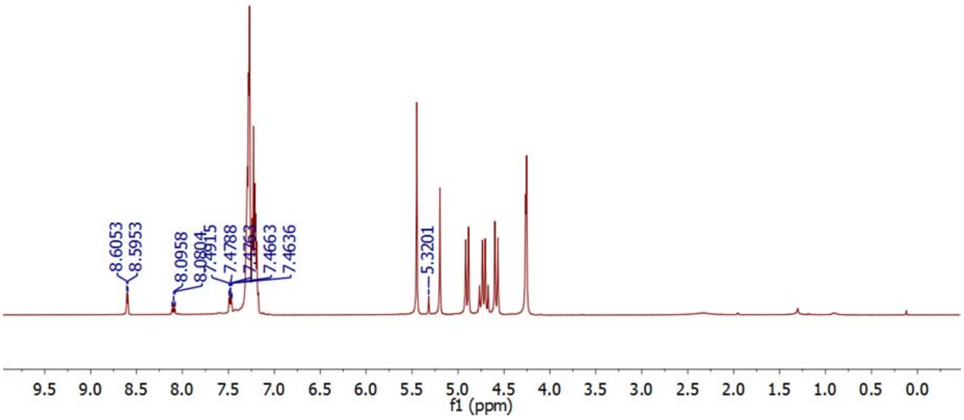

Figure S3 1.The ^{1}H NMR spectrum of [bis(pyridine)iodine] tetrafluoroborate (**9-I/9-I-*d***) in the presence of 1.8 eq of bambusuril acquired at 25°C in CD_2Cl_2 at 499.89 MHz.

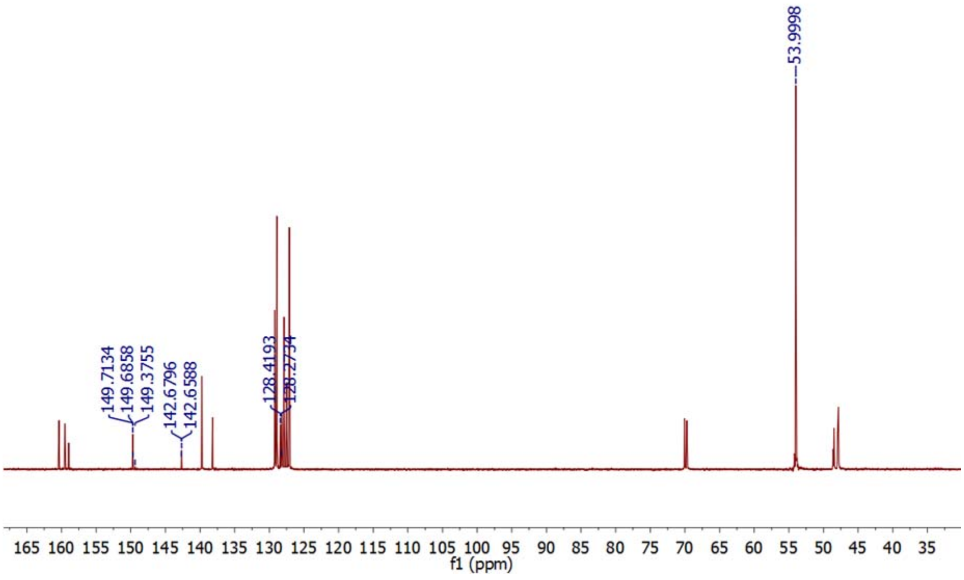

Figure S32. The ^{13}C NMR spectrum of [bis(pyridine)iodine] tetrafluoroborate (**9-I/9-I-*d***) in the presence of 1.8eq of bambusuril acquired at 25°C in CD_2Cl_2 at 125.61 MHz.

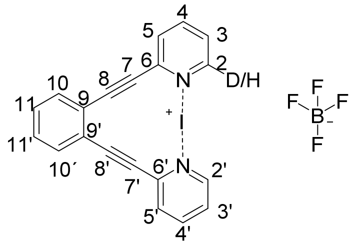

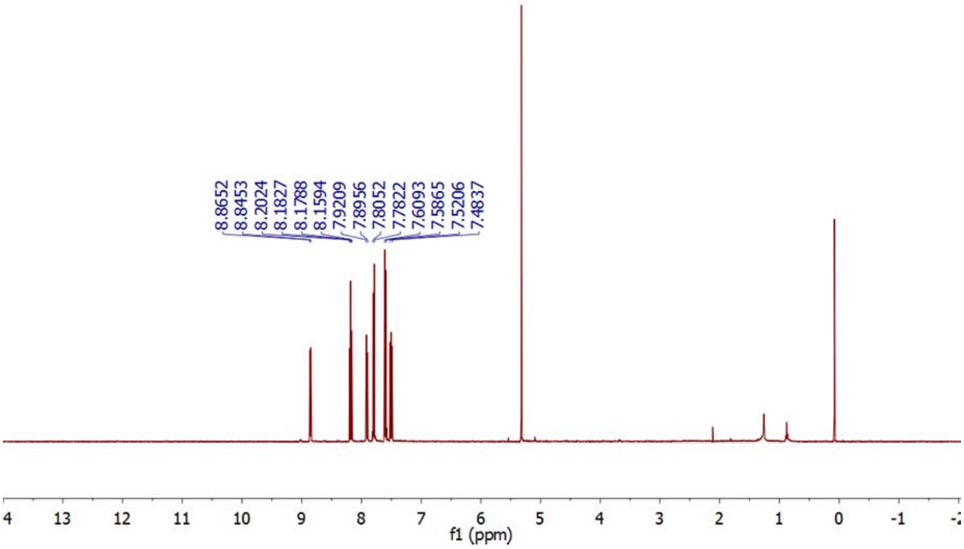

Figur S33. The ^{1}H NMR spectrum of [(0.-bis(pyridine-2-ylethynyl)benzene)iodine] tetrafluoroborate (**10-I/10-I-*d***) acquired at 25°C in CD_2Cl_2 at 499.89 MHz.

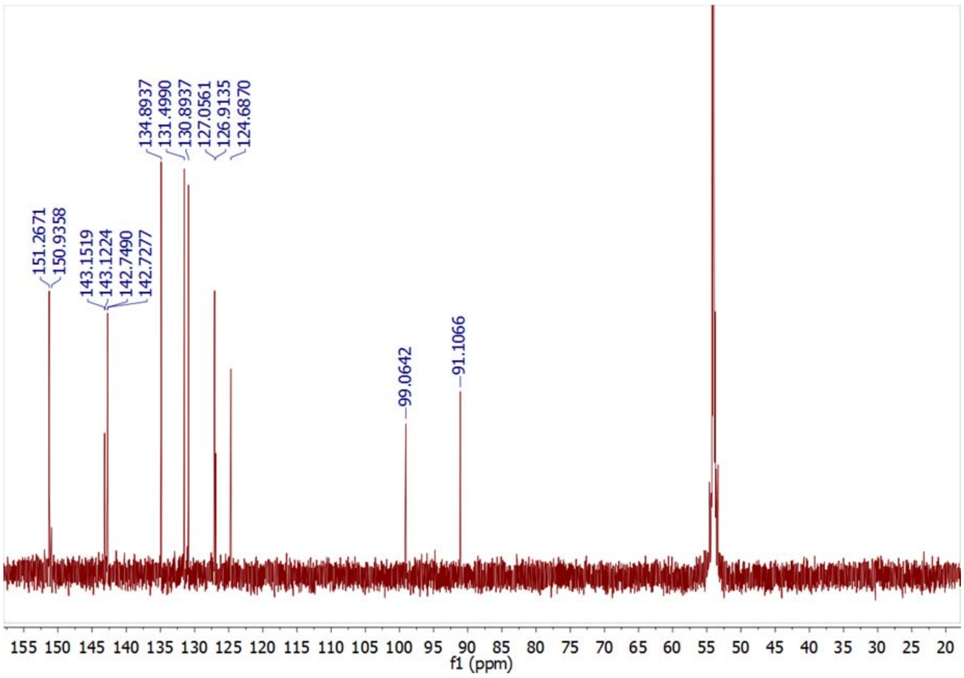

Figur S34. The ^{13}C NMR spectrum of [(0.-bis(pyridine-2-ylethynyl)benzene)iodine] tetrafluoroborate (**10-I/10-I-*d***) acquired at 25°C in CD_2Cl_2 at 125.61 MHz.

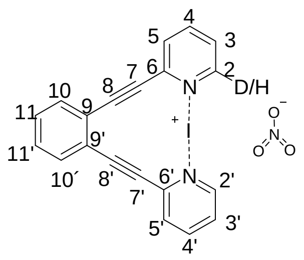

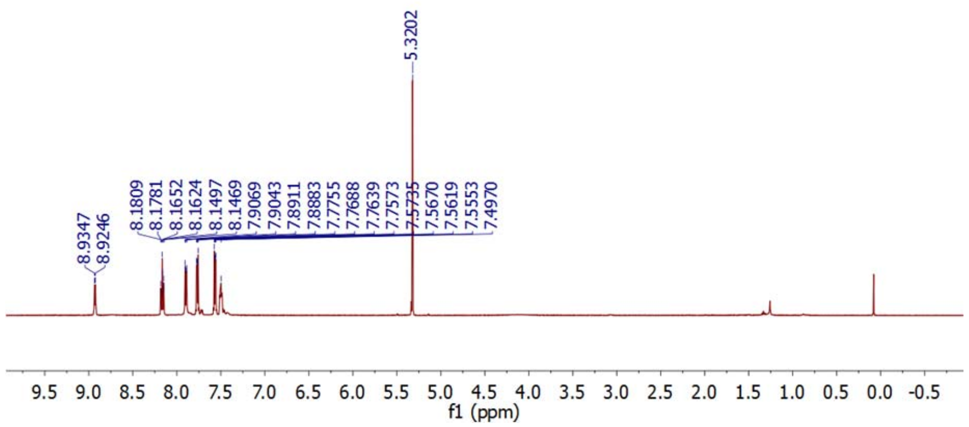

Figure S31. The ^{1}H NMR spectrum of [(0.-bis(pyridine-2-ylethynyl)benzene)iodine] nitrate (**12-I/12-I-*d***) acquired at 24°C in CD2Cl2 at 499.89 MHz.

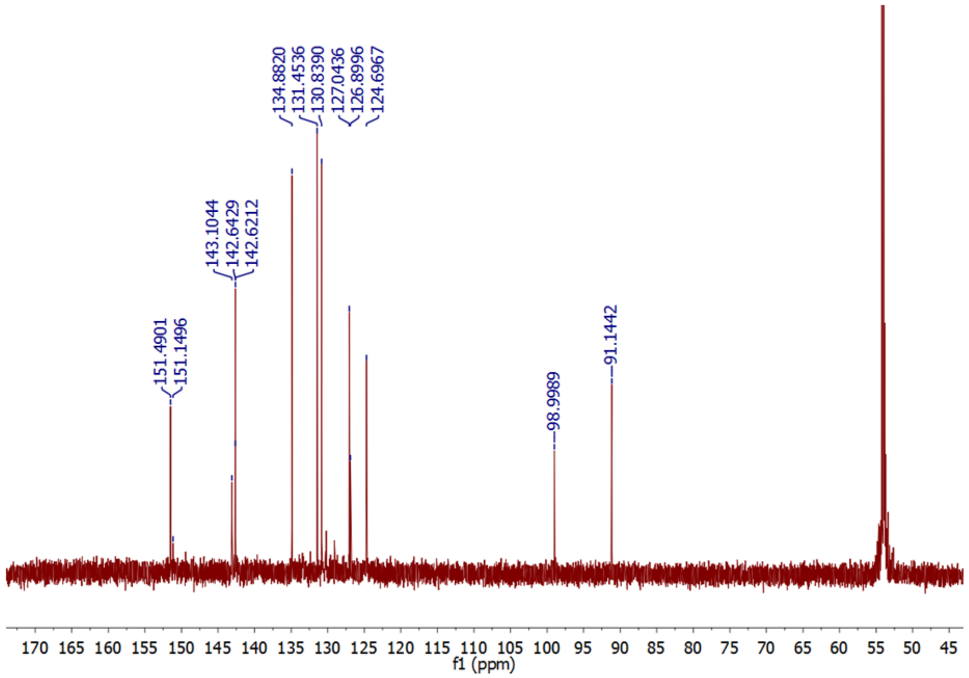

Figure S32. The ^{13}C NMR spectrum of [(0.-bis(pyridine-2-ylethynyl)benzene)iodine] nitrate (**12-I/12-I-*d***) acquired at 25°C in CD2Cl2 at 125.61 MHz.

Spectra for compound **11-I/11-I-*d*, 11-H/11-H-*d*** and **5-H/5-H-*d*** are given in references 1.

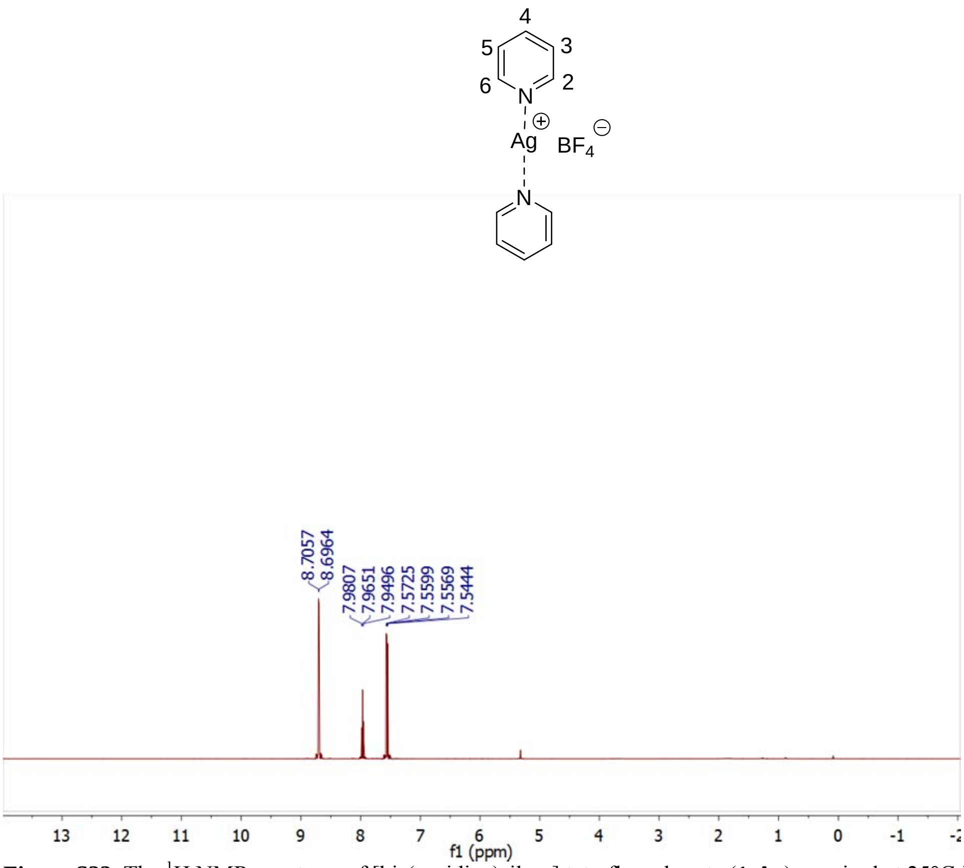

Figure S33. The ^{1}H NMR spectrum of [bis(pyridine)silver] tetrafluoroborate (**1-Ag**) acquired at 25°C in CD_2Cl_2 at 499.89 MHz.

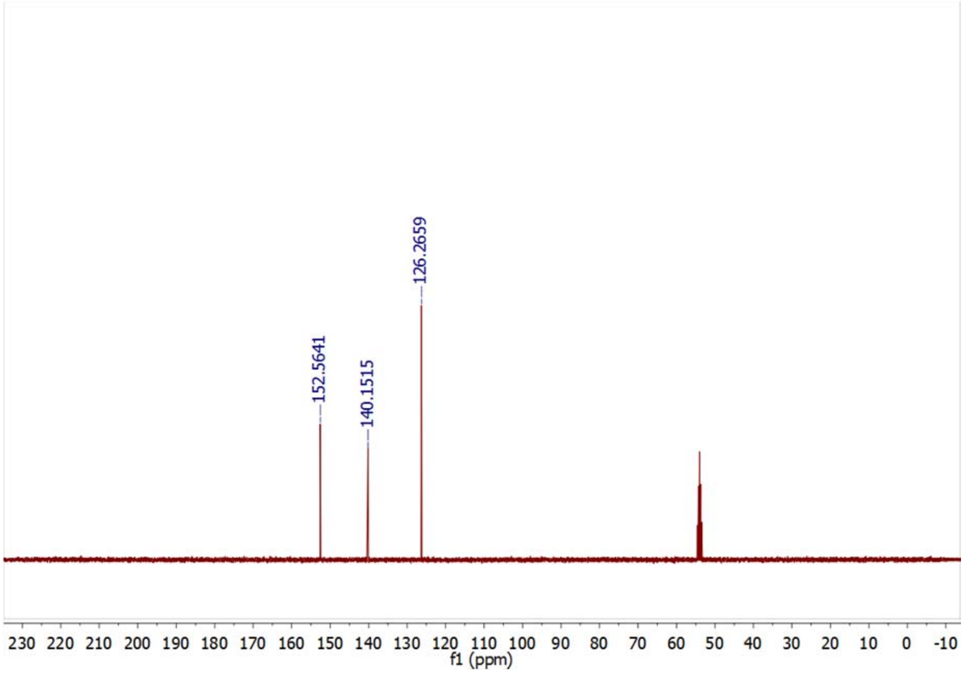

Figur S34. The ^{13}C NMR spectrum of [bis(pyridine)silver] tetrafluoroborate (**1-Ag**) acquired at 25°C in CD_2Cl_2 at 125.61 MHz.

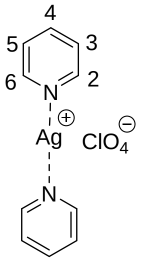

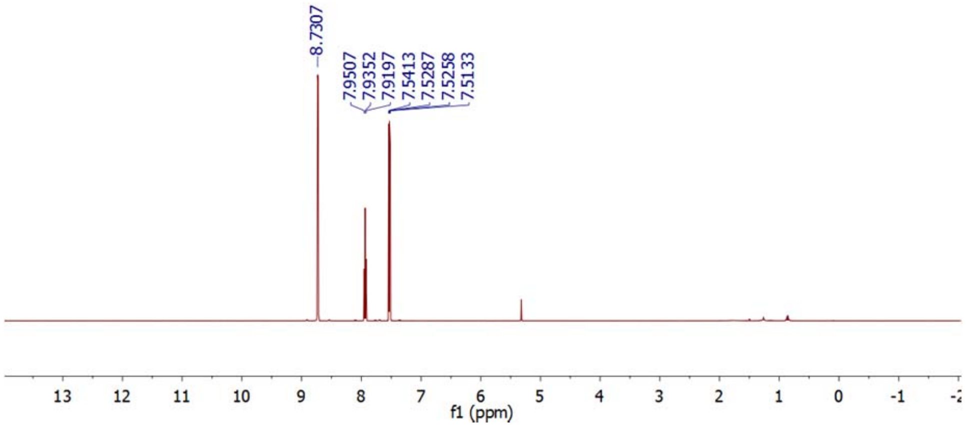

Figure S35.The ^{1}H NMR spectrum of [bis(pyridine)silver] perchlorate (**2-Ag**) acquired at 25°C in CD_2Cl_2 at 499.89 MHz

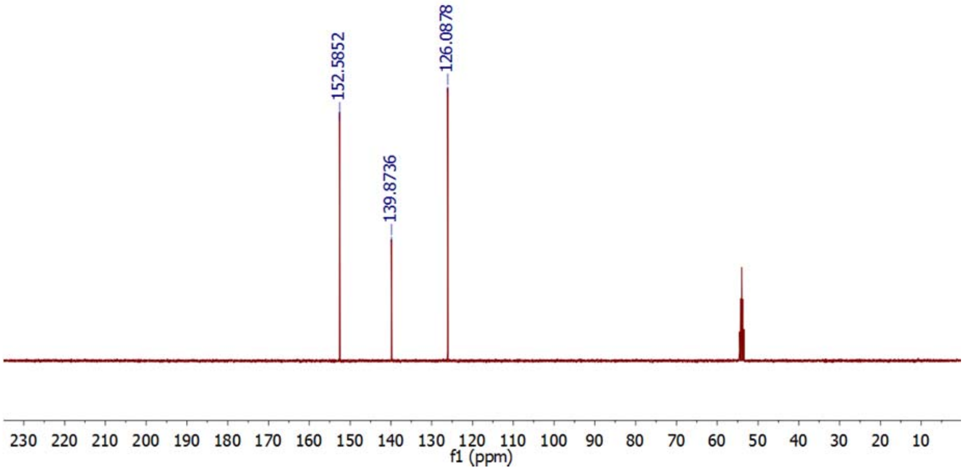

Figure S36. The ^{13}C NMR spectrum of [bis(pyridine)silver] perchlorate (**2-Ag**) acquired at 25°C in CD_2Cl_2 at 125.61 MHz.

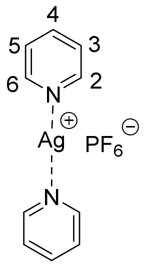

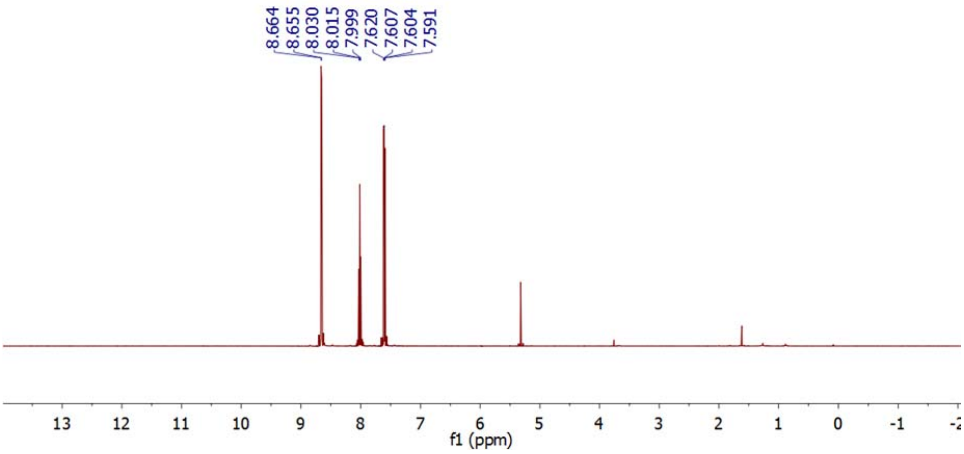

Figure S37. The ^{1}H NMR spectrum of [bis(pyridine)silver] hexafluorophosphate (**3-Ag**) acquired at 25°C in CD_2Cl_2 at 499.89 MHz.

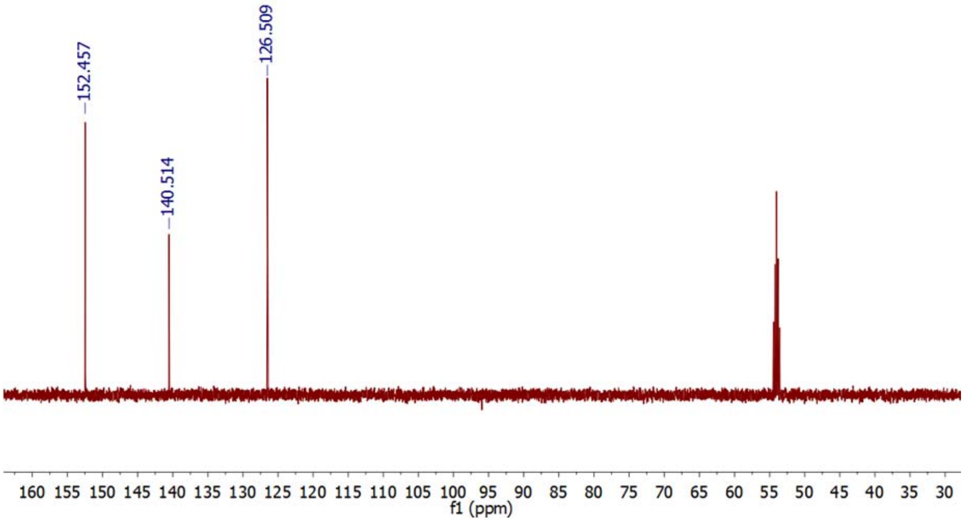

Figure S38. The ^{13}C NMR spectrum of [bis(pyridine)silver] hexafluorophosphate (**3-Ag**) acquired at 25°C in CD_2Cl_2 at 125.61 MHz.

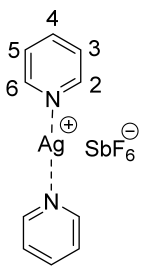

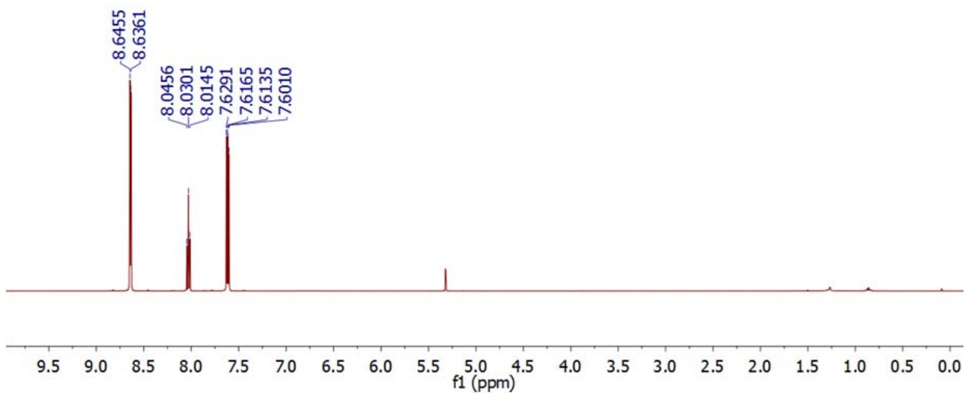

Figure S39. The ^{1}H NMR spectrum of [bis(pyridine)silver] hexafluoroantimonate (**4-Ag**) acquired at 25°C in CD_2Cl_2 at 499.89 MHz**.**

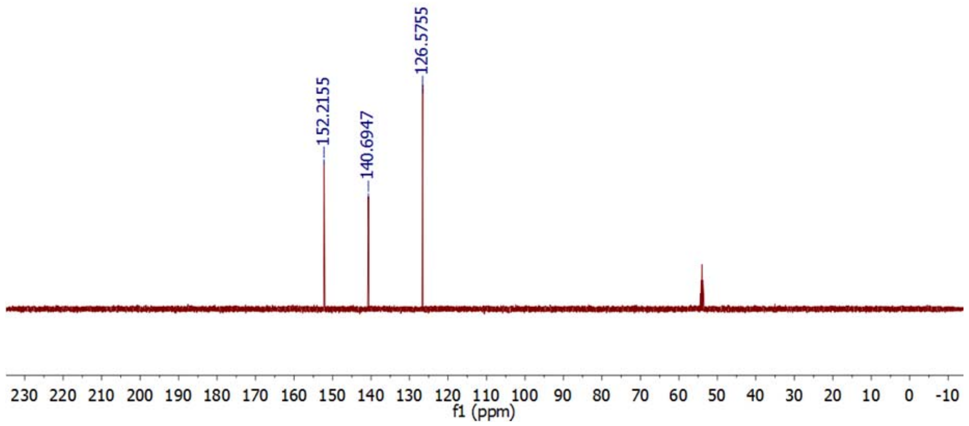

Figure S40. The ^{13}C NMR spectrum of [bis(pyridine)silver] hexafluoroantimonate (**4-Ag**) acquired at 25°C in CD_2Cl_2 at 125.61 MHz.

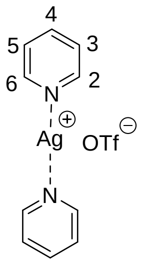

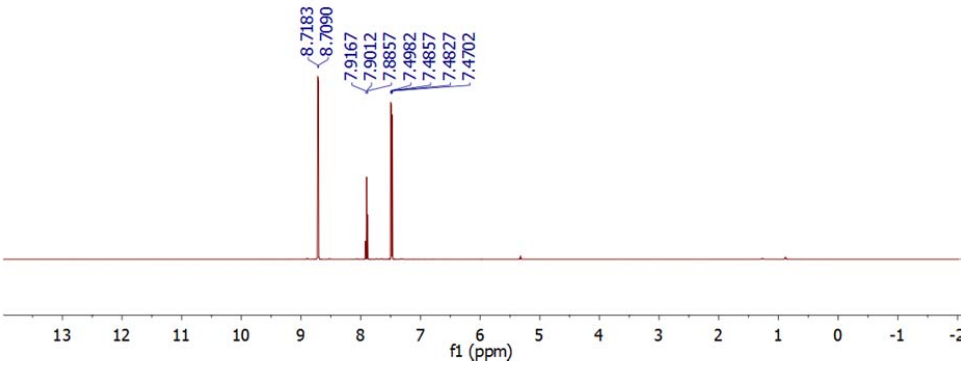

FigureS41. The ^{1}H NMR spectrum of [bis(pyridine)silver] triflate (**5-Ag**) acquired at 25°C in CD_2Cl_2 at 499.89 MHz.

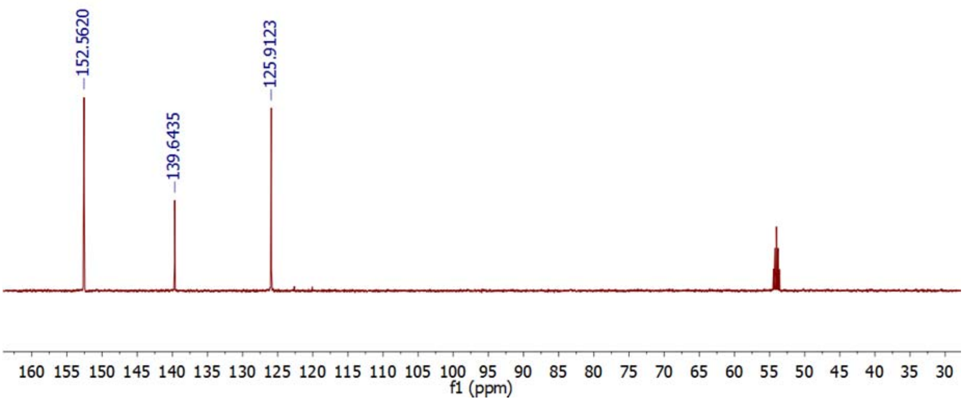

Figure S42. The ^{13}C NMR spectrum of [bis(pyridine)silver] triflate (**5-Ag**) acquired at 25°C in CD_2Cl_2 at 125.61 MHz.

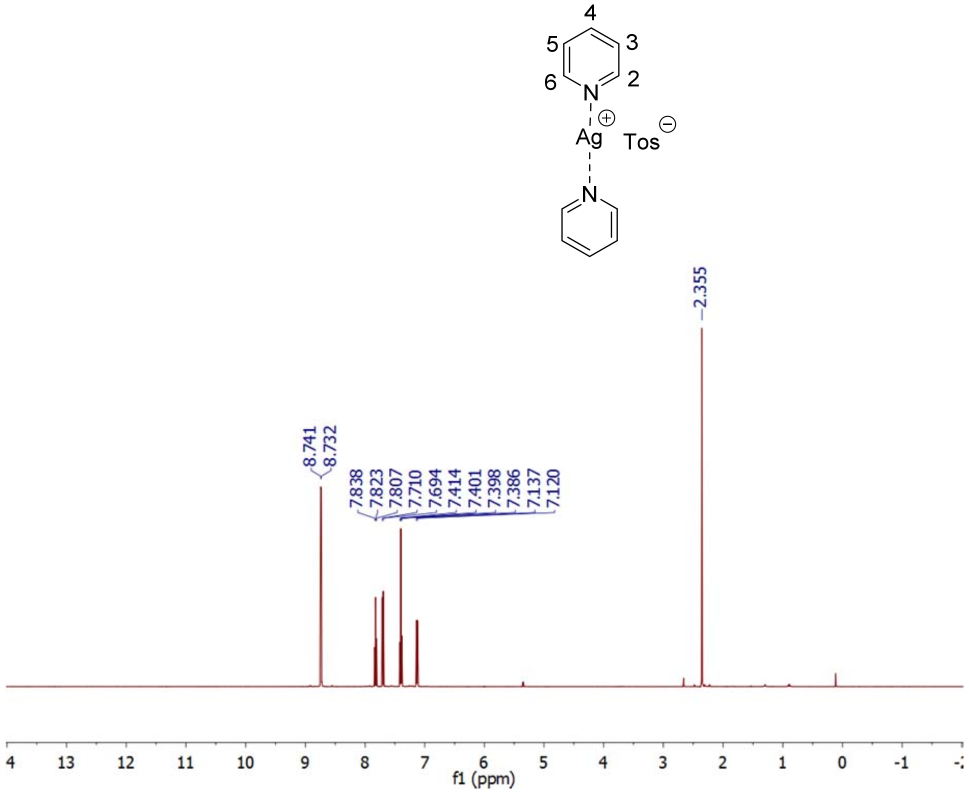

FigureS43. The ^{1}H NMR spectrum of [bis(pyridine)silver] tosylate (**6-Ag**) acquired at 25°C in CD_2Cl_2 at 499.89 MHz.

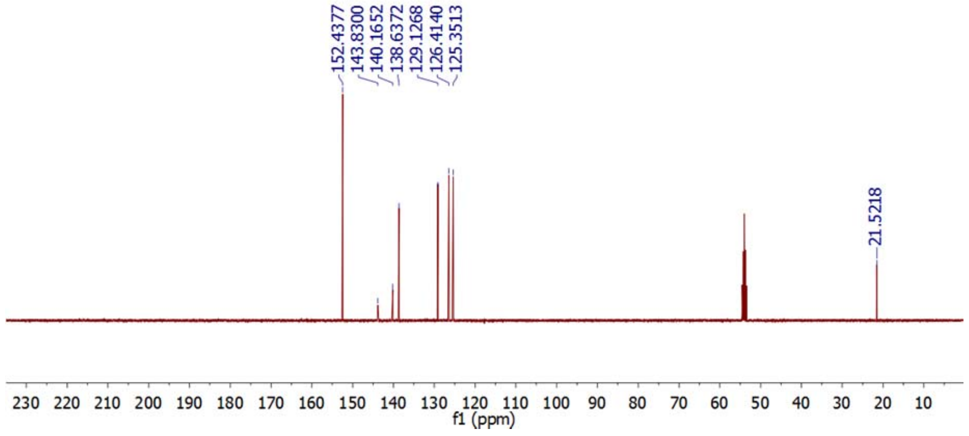

Figure S44. The ^{13}C NMR spectrum of [bis(pyridine)silver] tosylate (**6-Ag**) acquired at 25°C in CD_2Cl_2 at 125.61 MHz.

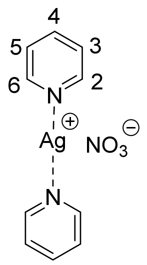

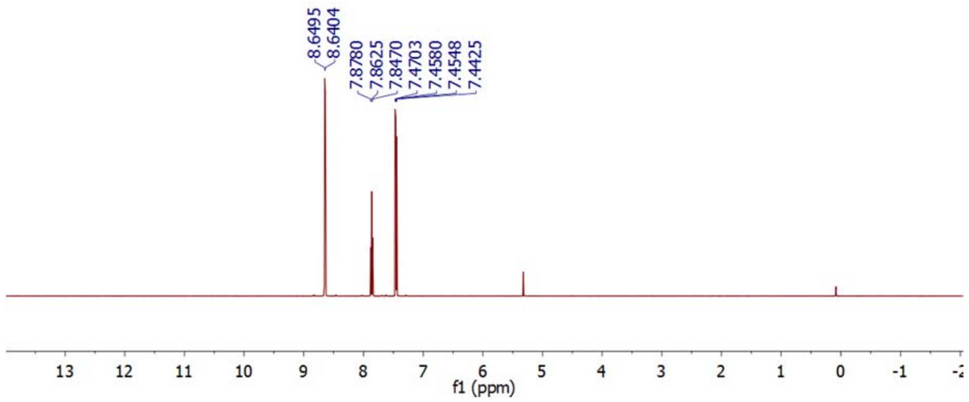

FigureS45. The ¹H NMR spectrum of [bis(pyridine)silver] nitrate (**7-Ag**) acquired at 25°C in CD_2Cl_2 at 499.89 MHz.

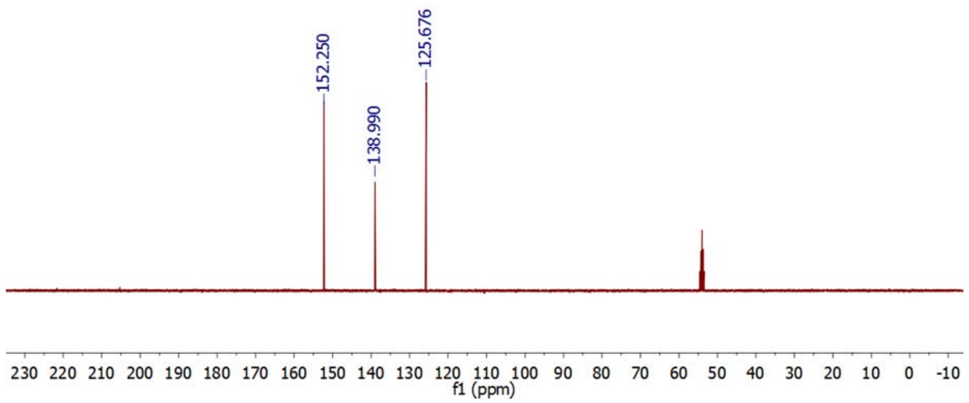

Figure S46. The ¹³C NMR spectrum of [bis(pyridine)silver] nitrate(**7-Ag**) acquired at 25°C in CD_2Cl_2 at 125.61 MHz.

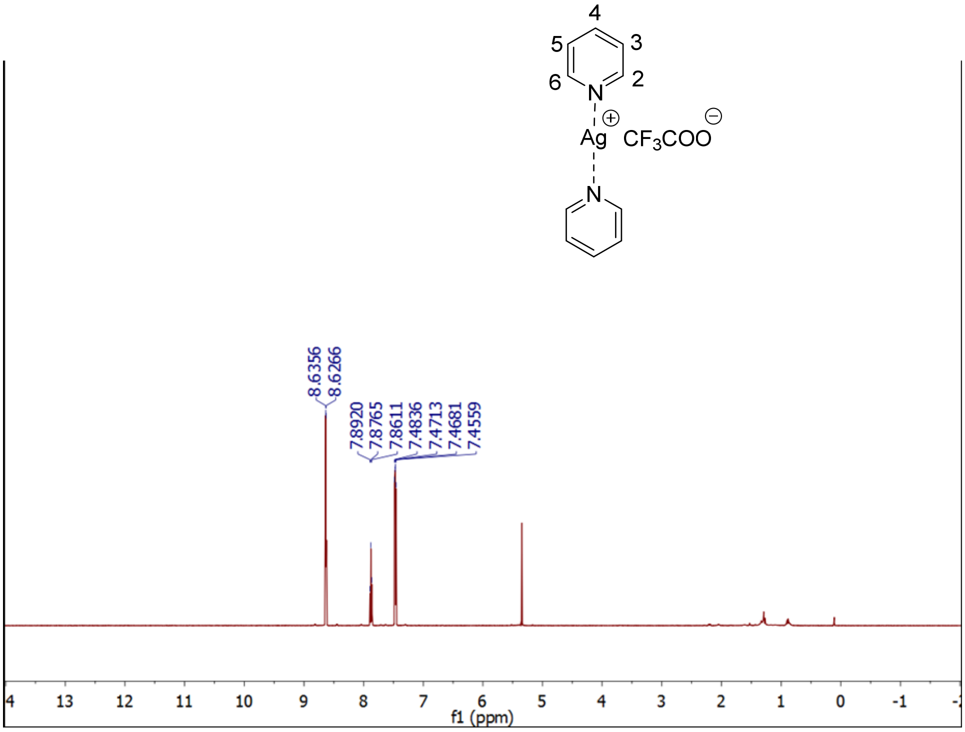

FigureS47. The ^{1}H NMR spectrum of [bis(pyridine)silver] trifluoroacetate (**8-Ag**) acquired at 25°C in CD_2Cl_2 at 499.89 MHz

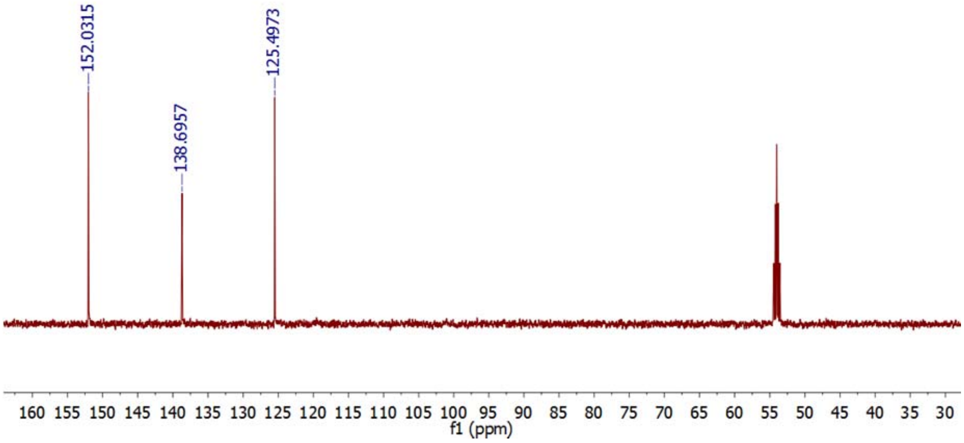

Figure S48. The ^{13}C NMR spectrum of [bis(pyridine)silver] trifluoroacetate(**8-Ag**) acquired at 25°C in CD_2Cl_2 at 125.61 MHz.

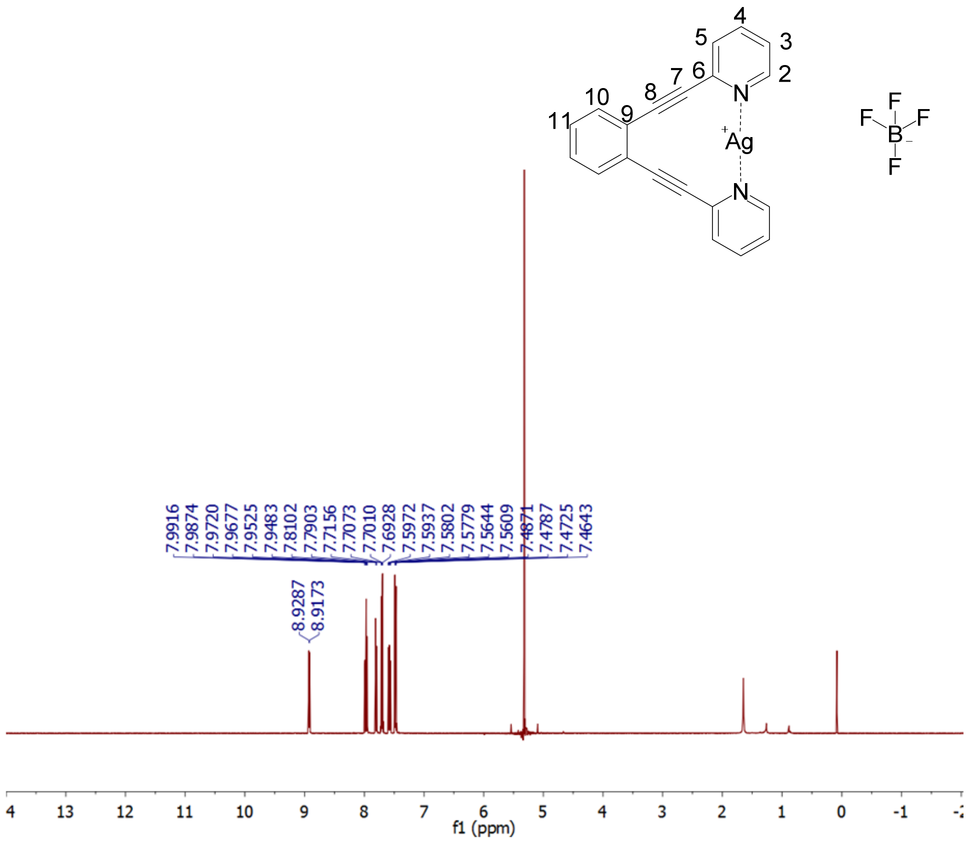

FigureS49. The ^{1}H NMR spectrum of [(1.2-bis(pyridin-2-ylethynyl)benzene)silver] tetrafluoroborate (**10-Ag**) acquired at 25°C in CD_2Cl_2 at 499.89 MHz.

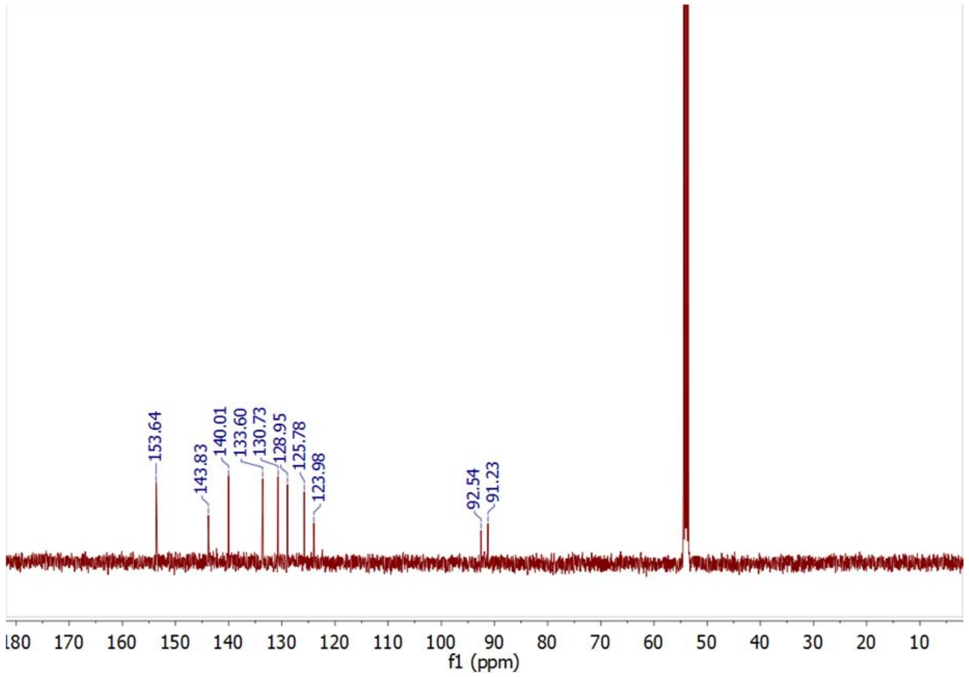

Figure S50. The ^{13}C spectrum of [(1.2-bis(pyridine-2-ylethynyl)benzene)silver] tetrafluoroborate (10-Ag) acquired at 25 ◦C in CD2Cl2 at 125.71 MHz.

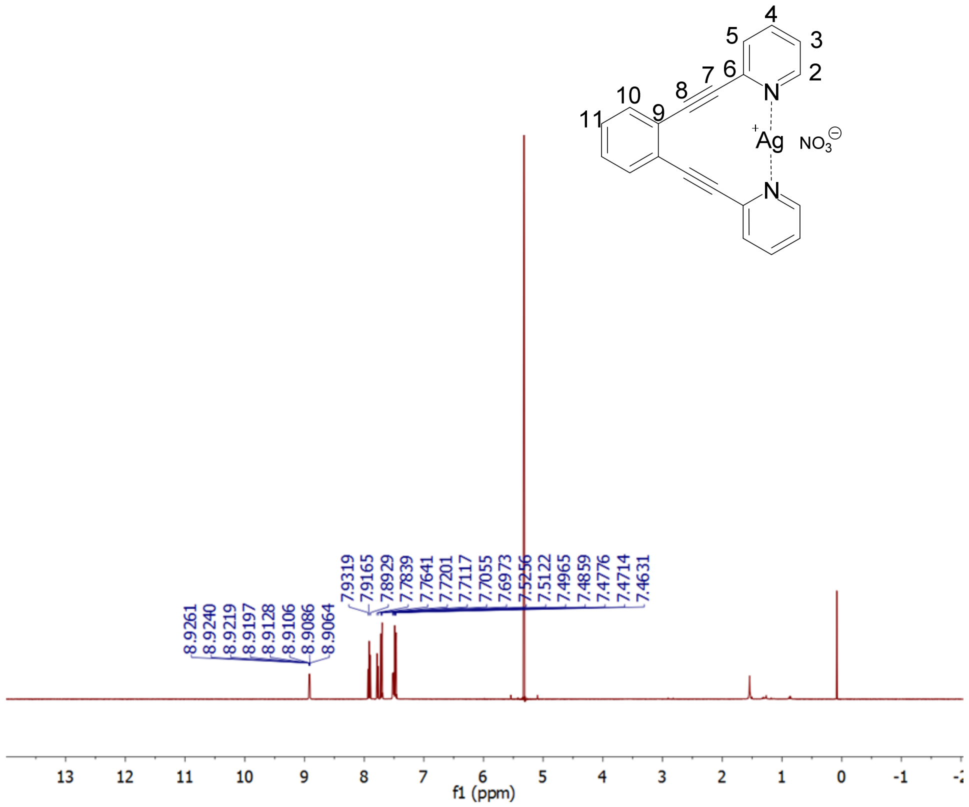

FigureS51. The ^{1}H NMR spectrum of [(1.2-bis(pyridin-2-ylethynyl)benzene)silver] nitrate (**12-Ag**) acquired at 25°C in CD_2Cl_2 at 499.89 MHz

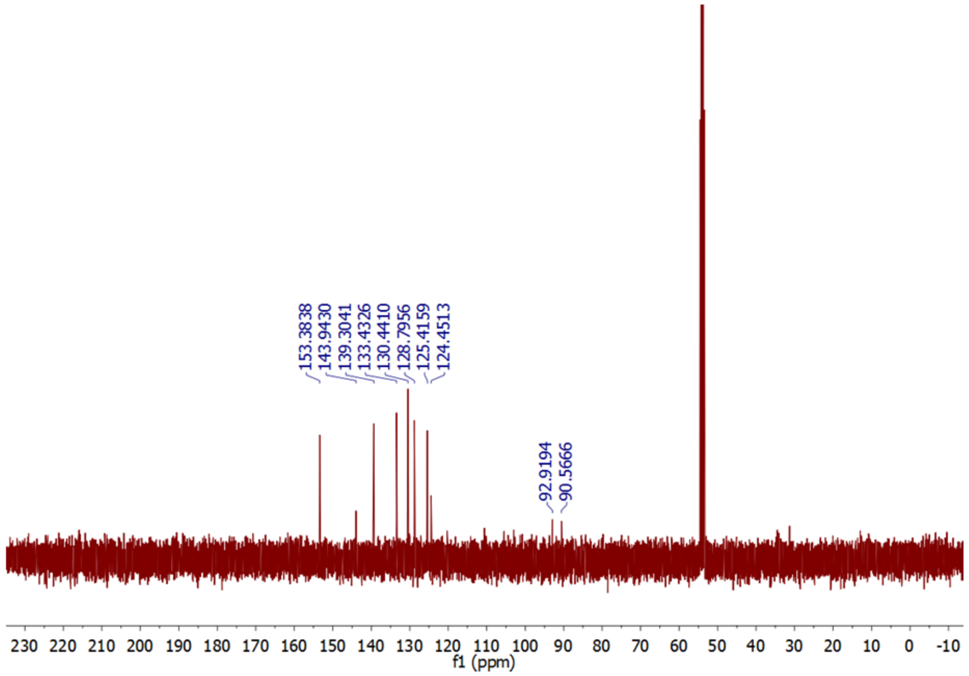

FigureS52. The ^{1}H NMR spectrum of [(-1.2-bis(pyridin-2-ylethynyl)benzene)silver] nitrate (12-Ag) acquired at 25°C in CD_2Cl_2 at 499.89 MHz.

7. References

1. A. C. Carlsson, J. Grafenstein, A. Budnjo, J. L. Laurila, J. Bergquist, A. Karim, R. Kleinmaier, U. Brath and M. Erdelyi, *J. Am. Chem. Soc.* **2012**, *134*, 5706.
2. A. C. Carlsson, J. Grafenstein, J. L. Laurila, J. Bergquist and M. Erdelyi, *Chem Commun* **2012**, *48*, 1458.
3. J. Svec, M. Necas and V. Sindelar, *Angew. Chem. Int. Ed. Engl.* **2010**, *49*, 2378.
4. J. Svec, M. Dusek, K. Fejfarova, P. Stacko, P. Klan, A. E. Kaifer, W. Li, E. Hudeckova and V. Sindelar, *Chem. Eur. J.* **2011**, *17*, 5605.
5. V. Havel, J. Svec, M. Wimmerova, M. Dusek, M. Pojarova and V. Sindelar, *Org. Lett.* **2011**, *13*, 4000.
6. A. D. Becke, *J. Chem. Phys.* **1993**, *98*, 5648.
7. C. T. Lee, W. T. Yang and R. G. Parr, *Phys.Rev. B* **1988**, *37*, 785.
8. S. H. Vosko, L. Wilk and M. Nusair, *Can. J. Phys.* **1980**, *58*, 1200.
9. P. J. Stephens, F. J. Devlin, C. F. Chabalowski and M. J. Frisch, *J. Phys. Chem.* **1994**, *98*, 11623.
10. A. D. Becke, *Physical Review A*, 1988, 38, 3098.
11. L. E. Roy, P. J. Hay and R. L. Martin, *J. Chem. Theor. Comput.* **2008**, *4*, 1029.
12. W. R. Wadt and P. J. Hay, *J. Chem. Phys.*, **1985**, *82*, 284.
13. P. J. Hay and W. R. Wadt, *J. Chem. Phys.*, **1985**, *82*, 270.
14. P. J. Hay and W. R. Wadt, *J. Chem. Phys.* **1985**, *82*, 299.
15. T. Clark, J. Chandrasekhar, G. W. Spitznagel and P. V. Schleyer, *J. Comput., Chem.* **1983**, *4*, 294.
16. M. M. Francl, W. J. Pietro, W. J. Hehre, J. S. Binkley, M. S. Gordon, D. J. Defrees and J. A. Pople, *J. Chem. Phys.***1982**, *77*, 3654.
17. R. Krishnan, J. S. Binkley, R. Seeger and J. A. Pople, *J. Chem. Phys.* **1980**, *72*, 650.
18. M. N. Glukhovtsev, A. Pross, M. P. Mcgrath and L. Radom, *J. Chem. Phys.* **1995**, *103*, 1878.
19. W. Kutzelnigg, U. Fleischer and M. Schindler, *Al - Cl: The IGLO-Method: Ab Initio Calculation and Interpretation of NMR Chemical Shifts and Magnetic Susceptibilities*, Springer-Verlag, Heidelberg, 1990.
20. M. Cossi, G. Scalmani, N. Rega and V. Barone, *J. Chem. Phys.* **2002**, *117*, 43.
21. B. Mennucci and J. Tomasi, *J. Chem. Phys.* **1997**, *106*, 5151.
22. A. D. Becke, *J. Chem. Phys.* **2003**, *119*, 2972.
23. J. Grafenstein and D. Cremer, *Theor. Chem. Account.* **2009**, *123*, 171.
24. D. C. Georgiou, P. Butler, E. C. Browne, D. J. D. Wilson and J. L. Dutton, *Aust. J. Chem.* **2013**, *66*, 1179.
25. A. Karim, M. Reitti, A. C. C. Carlsson, J. Grafenstein and M. Erdelyi, *Chem. Sci.*, **2014**, *5*, 3226.
26. C. Moller and M. S. Plesset, *Physical Review*, 1934, 46, 0618.
27. G. W. T. M. J. Frisch, H. B. Schlegel, G. E. Scuseria, M. A. Robb, J. R. Cheeseman, G. Scalmani, V. Barone, B. Mennucci, G. A. Petersson, H. Nakatsuji, M. Caricato, X. Li, H. P. Hratchian, A. F. Izmaylov, J. Bloino, G. Zheng, J. L. Sonnenberg, M. Hada, M. Ehara, K. Toyota, R. Fukuda, J. Hasegawa, M. Ishida, T. Nakajima, Y. Honda, O. Kitao, H. Nakai, T. Vreven, J. A. Montgomery, Jr., J. E. Peralta, F. Ogliaro, M. Bearpark, J. J. Heyd, E. Brothers, K. N. Kudin, V. N. Staroverov, R. Kobayashi, J. Normand, K. Raghavachari, A. Rendell, J. C. Burant, S. S. Iyengar, J. Tomasi, M. Cossi, N. Rega, J. M. Millam, M. Klene, J. E. Knox, J. B. Cross, V. Bakken, C. Adamo, J. Jaramillo, R. Gomperts, R. E. Stratmann, O. Yazyev, A. J. Austin, R. Cammi, C. Pomelli, J. W. Ochterski, R. L. Martin, K. Morokuma, V. G. Zakrzewski, G. A. Voth, P. Salvador, J. J. Dannenberg, S. Dapprich, A. D. Daniels, Ö. Farkas, J. B. Foresman, J. V. Ortiz, J. Cioslowski, and D. J. Fox, Gaussian, Inc., Walliongford CT, 2009.
